# RNase P/MRP subunits chaperone telomerase holoenzyme assembly in fission yeast

**DOI:** 10.1038/s44319-026-00782-9

**Published:** 2026-04-28

**Authors:** Lili Pan, Valentine Patterson, Martin M Möckel, Rachel M Helston, Raymund J Wellinger, David C Zappulla, Peter Baumann

**Affiliations:** 1 Department of Biology, Johannes Gutenberg University, Mainz, Germany; 2 Institute of Molecular Biology, Mainz, Germany; 3 Stowers Institute for Medical Research, Kansas City, MO USA; 4 Department of Microbiology and Infectious Diseases, Faculty of Medicine and Sciences, Université de Sherbrooke, Sherbrooke, QC Canada; 5 Department of Biological Sciences, Lehigh University, Bethlehem, PA USA; 6 Institute for Quantitative and Computational Biosciences, Johannes Gutenberg University, Mainz, Germany

**Keywords:** RNA Biology, Translation & Protein Quality

## Abstract

Telomerase biogenesis is a multistep process requiring the coordinated action of several accessory factors. In the fission yeast *Schizosaccharomyces pombe*, the telomerase RNA TER1 undergoes spliceosome-mediated 3′-end processing, followed by association with the Pof8/Bmc1/Thc1 complex, which facilitates binding of the Lsm2–8 complex. Lsm2–8 protects TER1 from nucleolytic degradation and promotes recruitment of the catalytic subunit Trt1. Here, we identify Pop6, Pop7, and Pop100, three subunits of the RNase P/MRP complex, as components of the active telomerase holoenzyme. These proteins associate with a stem–loop–stem structure near the TER1 pseudoknot that resembles the P3 domain found in RNase P/MRP RNAs. A single-nucleotide change within this P3-like loop disrupts Pop protein binding, resulting in reduced telomerase activity and severe telomere shortening. This mutation also impairs the assembly of key telomerase subunits and alters the folding of the template–pseudoknot region of TER1. Our findings reveal a critical role for Pop6, Pop7, and Pop100 in chaperoning TER1 into a conformation that promotes functional telomerase assembly and underscore the remarkable evolutionary plasticity of telomerase biogenesis.

## Synopsis

RNase P/MRP subunits Pop6/7/100 chaperone folding of the telomerase RNA core via a P3-like element, enabling holoenzyme assembly and function. This highlights the reuse of ancient RNA-binding modules across distinct RNP biogenesis pathways.

- RNase P/MRP subunits Pop6, Pop7, and Pop100 are integral components of active telomerase and bind a P3-like element in TER1.
- A single nucleotide change in the P3-like loop disrupts Pop binding, impairing telomerase assembly and activity and causing severe telomere shortening.
- Pop proteins chaperone folding of the TER1 template–pseudoknot core, enabling subunit assembly and establishing reciprocal stabilization within the holoenzyme.

## Abstract (web summary)

RNase P/MRP subunits Pop6/7/100 chaperone folding of the telomerase RNA core via a P3-like element, enabling holoenzyme assembly and function. This highlights the reuse of ancient RNA-binding modules across distinct RNP biogenesis pathways.

## Graphical abstract

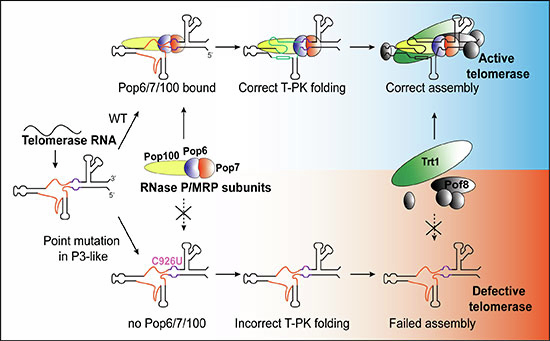

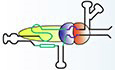

## Introduction

Telomerase is an RNA-dependent DNA polymerase that extends the telomeric repeats at the ends of eukaryotic chromosomes. The core components of the enzyme are the protein subunit Telomerase Reverse Transcriptase (TERT) and the Telomerase RNA (TR), which provides the template and acts as a scaffold for holoenzyme assembly (Qin and Autexier, 2021). Additional protein subunits are involved in the biogenesis, regulation, and recruitment of the enzyme. Mutations in genes related to telomerase and telomeres are associated with a range of premature aging disorders with diverse clinical manifestations collectively referred to as Telomere Biology Disorders (TBDs) (Revy et al, 2023). Unlike the TERT protein, which is highly conserved in sequence and structure, the telomerase RNA subunit varies substantially in length and sequence among species (Podlevsky and Chen, 2016). Despite this variability, several conserved structural elements have been identified, such as a pseudoknot (PK), a template boundary element (TBE), and a functional module involved in catalytic core assembly. This module takes the form of a stem-terminus element (STE) in ciliates, the conserved regions 4 and 5 (CR4/5) in vertebrates, and structurally distinct but functionally analogous elements in fungi, including three-way junctions (TWJ). While these elements differ in sequence and secondary structure, they share the critical roles in facilitating TERT binding and holoenzyme assembly (Podlevsky and Chen, 2016).

In the fission yeast *Schizosaccharomyces pombe*, the Telomerase RNA (TER1) is transcribed as a precursor containing two exons and one intron (Box et al, 2008). The evolutionarily ancient Sm complex associates with the unspliced precursor of TER1 and is required for its processing and stability (Box et al, 2008; Leonardi et al, 2008; Webb and Zakian, 2008). Processing by the spliceosome deviates from a canonical splicing reaction in that it only involves the first transesterification reaction, followed by the release of the first exon as the processed form of TER1 (Box et al, 2008). Uncoupling of the first and second steps of splicing is promoted by elements within the intron that favor release of the splicing intermediates (Kannan et al, 2013). This alternate role of the spliceosome as a 3’-end processing machinery appears to be conserved among several fungal species, but the underlying mechanisms that uncouple cleavage at a 5’ splice site from the remainder of a splicing reaction are remarkably diverse (Gunisova et al, 2009; Kannan et al, 2015; Kuprys et al, 2013; Qi et al, 2015; Smekalova et al, 2013; Volanakis et al, 2013). Spliceosomal cleavage causes the Sm complex to dissociate from TER1, followed by the binding of the related Lsm2-8 complex, which is required for 3’-end protection and recruitment of the TERT protein (Trt1 in *S. pombe*) to TER1 (Tang et al, 2012).

Additional proteins are part of the holoenzyme and are required for the biogenesis and function of telomerase. Pof8 (Pombe F-box 8), a La-related protein (LARP), binds the pseudoknot region of TER1 and promotes the association of Lsm2-8 complex with TER1 (Collopy et al, 2018; Hu et al, 2020; Mennie et al, 2018; Paez-Moscoso et al, 2018). Pof8 functions together with Bmc1 (Bin3/MePCE 1), the ortholog of MePCE (Methyl phosphate capping enzyme) and Thc1 (Telomerase Holoenzyme Component 1), which contains a domain of structural similarity with human PARN (Poly-adenosine ribonuclease) (Paez-Moscoso et al, 2022; Porat et al, 2022). Interestingly, the metazoan orthologs of Pof8 and Bmc1 are both components of the 7SK ribonucleoprotein (RNP) complex, which functions in transcription regulation in metazoans (Hasler et al, 2021). A 7SK RNA and associated RNP have not been identified in fission yeast. Crosstalk between telomerase and various other RNPs including the spliceosome, snRNPs, and 7SK has been a recurring theme in the study of telomerase biogenesis across various species (Fu and Collins, 2006, 2007; Gunisova et al, 2009; Hass and Zappulla, 2020; Kao et al, 2024; Seto et al, 1999). The Sm complex also binds telomerase RNA in budding yeast, where it is essential for processing and stability (Gunisova et al, 2009; Hass and Zappulla, 2020; Seto et al, 1999). In addition, a subset of the Sm proteins has been reported to associate with human telomerase RNA, although their role in telomerase biogenesis is less clear (Fu and Collins, 2006, 2007). The human Pof8 ortholog LARP7, along with the related factor LARP3, as well as the Bmc1 ortholog MePCE, also function in the early stages of human telomerase biogenesis (Kao et al, 2024).

Ribonuclease P (RNase P) and the closely related RNase MRP are essential RNPs that each consist of a unique catalytic RNA and a largely overlapping set of around 10 protein subunits (Esakova and Krasilnikov, 2010). RNase P and MRP RNAs are found across eukaryotes and archaea and carry out fundamental roles in tRNA, rRNA, and mRNA processing. Among the protein subunits, Pop6 (processing of precursor RNAs 6) and Pop7 bind as a heterodimer to a stem–loop–stem structure called the P3 domain in the respective RNA subunits, while Pop1 contacts multiple regions of the RNA, including the P3 domain (Fagerlund et al, 2015; Hands-Taylor et al, 2010; Perederina et al, 2007). Surprisingly, Pop1, Pop6 and Pop7, but not the other subunits of RNase P/MRP, also associate with a P3-like domain in TLC1, the telomerase RNA subunit in the budding yeast *Saccharomyces cerevisiae* (Lemieux et al, 2016; Lin et al, 2015), where it promotes the recruitment of Est1 and Est2 (Garcia et al, 2020; Laterreur et al, 2018; Lemieux et al, 2016).

Here, we show that the Pop1 ortholog Pop100, and the Pop6 and Pop7 proteins are an integral part of active telomerase in fission yeast. Unlike TLC1, where the P3-like domain is near the terminus of the long stem IVc, the binding site in TER1 is an internal stem–loop–stem region near the pseudoknot (PK) of the RNA. Mutations in the P3-like loop sequence reduced Pop6/7/100 binding, diminished telomerase activity, and caused severe telomere shortening in vivo. This phenotype is caused by a general assembly defect affecting multiple telomerase subunits, including Pof8, Trt1, and Est1, when binding of Pop6/7/100 is impaired. Further structural probing by in-cell SHAPE-MaP analysis revealed a specific structural change of TER1 in the P3-like and template-pseudoknot (T-PK) region caused by the mutation in the P3-like loop, indicating that Pop binding is important for the correct folding of the core T-PK region of the RNA. Intriguingly, deletion of *pof8* or *trt1* showed similar structural changes in the T-PK and P3-like region, and reciprocally reduced binding of Pop6/7/100 to TER1. Together, our results reveal critical roles of Pop6/7/100 in chaperoning TER1 into correct folding and support a model of reciprocal stabilization in RNP complex formation.

## Results

### Identification of Pop6 ortholog in *S. pombe*

The involvement of Pop1, Pop6, and Pop7 in telomerase assembly in *Saccharomyces cerevisiae* prompted us to investigate whether the association of RNaseP/MRP components with telomerase is conserved in the evolutionarily distant fission yeast *Schizosaccharomyces pombe*. We first sought to identify Pop6 orthologs in *S. pombe*, since none were annotated in Pombase at the time. A proteomic study aimed at identifying components of *S. pombe* RNase MRP using epitope-tagged Rmp1 had previously detected orthologs of most subunits shared with *S. cerevisiae* and human RNase MRP, with the exception of Pop6 (Saito et al, 2014).

A database search using human Pop6 ortholog RPP25 against the *S. pombe* proteome via the HHpred server (Hildebrand et al, 2009) returned two candidates, Pop7 and a previously uncharacterized sequence orphan named New2 (SPAC1805.18). This 95-amino-acid sequence was originally identified computationally during a screen for putative novel proteins in *S. pombe* (Bitton et al, 2011). Structural comparisons of New2 to human RPP25 and *S. cerevisiae* Pop6 using HHpred revealed similarities between the N-terminal two-thirds of New2 and human RPP25 and almost the entire New2 and *S.c* Pop6 (Appendix Fig. S1A,B). Overlay of the AlphaFold-predicted structure of New2 (Varadi et al, 2024) (Appendix Fig. S1C) with the crystal structure of *H.s* RPP25 (Chan et al, 2018) showed strong concordance, with a root-mean-square deviation (RMSD) of 1.004 Å across α-carbon pairs of 52 residues (Appendix Fig. S1D). Comparable results were obtained when overlaying New2 with *S.c* Pop6 (Perederina et al, 2010), yielding an RMSD of 1.008 Å across α-carbon pairs of 42 residues (Appendix Fig. S1E). Multimeric modeling by AlphaFold predicted interactions between New2 and Pop7, and superimposition of this complex with human RPP25–RPP20 revealed an RMSD of 1.116 Å across α-carbon pairs of 94 residues (Appendix Fig. S1F). Taken together, these sequence and structural similarities strongly support the identification of New2 (SPAC1805.18) as the *S. pombe* ortholog of Pop6. Attempts to knock out *pop6*^*+*^ in a haploid WT cell did not yield colonies, while making a heterozygous diploid *pop6*^+^/*pop6*∆ strain was successful (Appendix Fig. S2A,B). We then performed random spore analysis for two independent isolates. For 230 and 93 spores that formed colonies on non-selective media, zero colonies were observed on plates containing G418 to select for cells harboring the *pop6* deletion (Appendix Fig. S2C). These results are consistent with *pop6*^+^ being an essential gene in *S. pombe*, similar to its ortholog in *S. cerevisiae* (Chamberlain et al, 1998) and consistent with the essential functions of RNase P/MRP.

**Figure 1:**
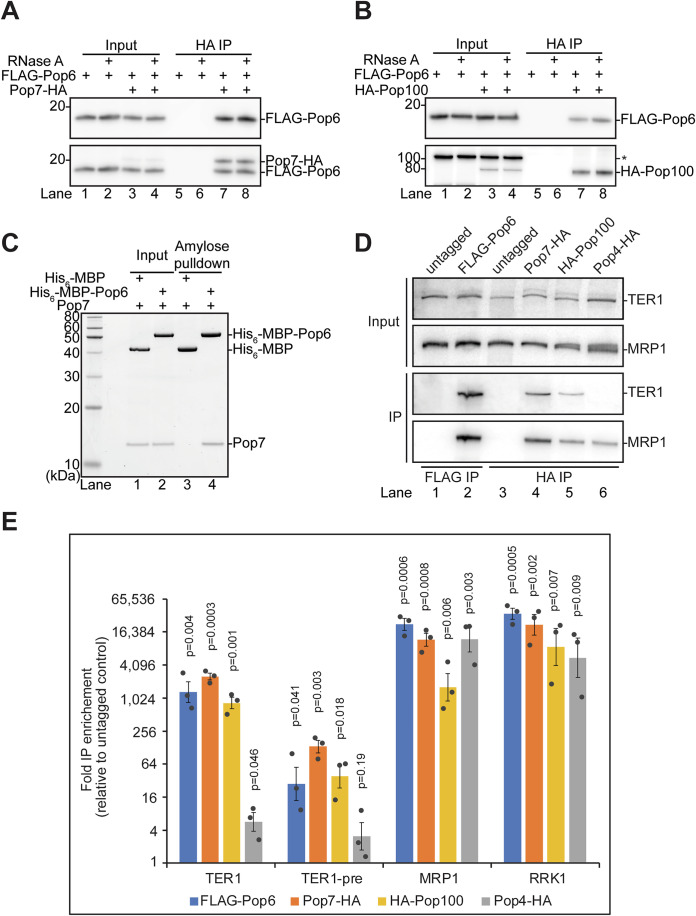
Pop6, Pop7, and Pop100 bind to TER1. (**A**) Co-immunoprecipitation (Co-IP) of FLAG-tagged Pop6 with HA-tagged Pop7. Extracts with or without RNase A treatment were immunoprecipitated using anti-HA antibody. Western blot of input and IP fractions was probed with anti-FLAG (top) to detect FLAG–Pop6 and reprobed with anti-HA (bottom) to detect Pop7-HA. (**B**) Co-IP of FLAG-tagged Pop6 with HA-tagged Pop100, performed as in (**A**). Western blot was probed with anti-FLAG (top) to detect FLAG–Pop6 and reprobed with anti-HA (bottom) to detect HA-Pop100. The asterisk labels a non-specific band recognized by the anti-FLAG antibody from the first probing that is present in all input samples. (**C**) Pulldown of recombinant Pop7 by His₆-MBP-Pop6 or control His₆-MBP (negative control) using amylose resin, followed by SDS-PAGE and Coomassie staining. (**D**) Northern blot detection of TER1 and MRP1 recovered from IPs. FLAG IPs were performed on untagged control or FLAG–Pop6 extracts (lanes 1–2), and HA IPs on untagged control, Pop7-HA, HA-Pop100, or Pop4-HA extracts (lanes 3–6). Blots for input RNA (top two panels) and IP RNA (bottom two panels) were probed with a TER1 probe and reprobed with an MRP1 probe. (**E**) RT–qPCR quantification of TER1, TER1-pre, MRP1, and RRK1 (RNA subunit of RNase P) recovered from FLAG IP on FLAG–Pop6 extracts, compared to untagged control extract and HA IPs on tagged Pop7, Pop100, and Pop4 extracts, compared to untagged control extract. Bars show mean enrichment ( ± SEM, *n* = 3) of IP RNA in the tagged samples compared to the untagged controls after normalization to input and expressed as fold change (with log₂ axis). Dots represent individual biological replicates. Statistical significance was assessed using unpaired *t* tests (*n* = 3) on -∆∆CT values, comparing tagged samples with the corresponding untagged controls. The respective *P* values are shown above each bar. Source data are available online for this figure.

To further verify whether Pop6 is indeed the ortholog, we experimentally tested the predicted interaction between Pop6 and Pop7 by co-immunoprecipitation assays. HA-tagged Pop7 efficiently pulled down FLAG-tagged Pop6 (Fig. 1A). This interaction was not RNA-dependent, as RNase A treatment did not diminish the amount of Pop6 co-precipitating with Pop7 (Fig. 1A, compare lanes 7 and 8). Similarly, HA-tagged Pop100 co-precipitated FLAG-tagged Pop6 in an RNA-independent manner (Fig. 1B). To determine whether the Pop6–Pop7 interaction is direct, we recombinantly expressed both proteins in *E. coli*, and purified them in the RNA-free state (Appendix Fig. S3). Purified His₆-MBP-Pop6, but not His₆-MBP, which served as a background binding control, pulled down untagged Pop7 when co-incubated in vitro, confirming a direct interaction between Pop6 and Pop7 that is independent of RNA and other RNase P/MRP subunits (Fig. 1C).

### Pop6, Pop7, and Pop100 associate with TER1

RNA purified from Pop6, Pop7, and Pop100 immunoprecipitations and subjected to northern blotting revealed the presence of MRP RNA (MRP1), the RNA subunit of RNase MRP (Fig. 1D). In addition, all three proteins also associated with TER1 (Fig. 1D). In contrast, the RNase P/MRP subunit Pop4 co-precipitated MRP1, but did not associate with TER1 (Fig. 1D). The enrichment of TER1 in the immunoprecipitates was further confirmed by RT–qPCR. Immunoprecipitation of Pop6, Pop7, and Pop100 showed an approximately 1000-fold enrichment of TER1 relative to the untagged control (Fig. 1E). Interestingly, using primers specifically targeting the sequence downstream of the mature 3’-end of TER1 showed that Pop6, Pop7 and Pop100 are already associated with the precursor form of TER1 (TER1-pre), indicating that Pop proteins function during the early steps of TER1 biogenesis (Fig. 1E).

### Pop6, Pop7, and Pop100 are components of active telomerase

**Figure 2:**
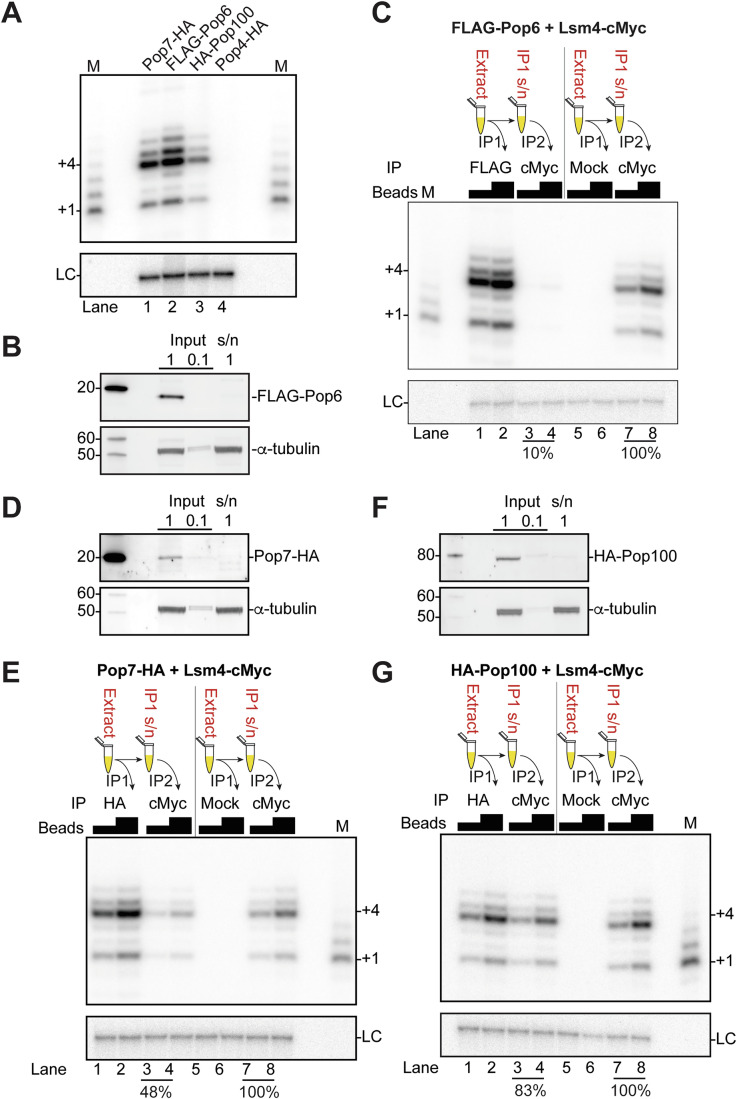
Pop6, Pop7, and Pop100 are components of active telomerase. (**A**) Telomerase activity assays from FLAG–Pop6, Pop7-HA, HA-Pop100, and Pop4-HA IPs. A ladder of extension products generated by terminal transferase served as a size marker (M). A ³²P-labeled 100-mer oligonucleotide was used as a precipitation and loading control (LC). (**B**) Western blot of input (undiluted and 1:10 dilution) and supernatant (s/n) from the first FLAG IP in a sequential IP experiment. The blot was probed with anti-FLAG (top) to detect FLAG–Pop6 and then reprobed with anti-α-tubulin (bottom) as a loading control. (**C**) Telomerase activity assay following sequential IPs to assess Pop6-associated activity. Extracts containing both FLAG–Pop6 and Lsm4-cMyc were first immunoprecipitated with either anti-FLAG or anti-V5 (Mock). The supernatant (s/n) fractions from the first round of IPs were used as input for a second round of IP with anti-c-Myc. Telomerase activity was analyzed using 10 µL and 20 µL of each IP. The quantification of the average activity in lane 3 and 4 normalized to average of lane 7 and 8 are indicated below. (**D**) Western blot of input and s/n from the first HA IP in sequential IP, detecting Pop7-HA (top) and α-tubulin (bottom) as in (**B**). (**E**) Telomerase activity assay following sequential IPs to assess Pop7-associated activity with extracts containing both Pop7-HA and Lsm4-cMyc, performed as in (**C**). (**F**) Western blot of input and s/n from the first HA IP detecting HA-Pop100 and α-tubulin. (**G**) Telomerase activity assay following sequential IPs to access Pop100-associated activity with extracts containing both HA-Pop100 and Lsm4-cMyc, analyzed as in (**C**). Source data are available online for this figure.

Since the three Pop proteins were found to associate with the telomerase RNA precursor, we next tested whether they remain associated throughout RNP maturation and are ultimately components of the mature active enzyme complex. Telomerase activity was associated with immunoprecipitates of Pop6, Pop7, and Pop100, but not with Pop4 (Fig. 2A). Consistent with the relative amounts of TER1 co-precipitated by each protein (Fig. 1D), activity was strongest in FLAG–Pop6 and weakest in HA–Pop100 immunoprecipitates (Fig. 2A).

To further assess what fraction of active telomerase is associated with each Pop protein, we performed sequential immunoprecipitations followed by telomerase activity assays. Protein extracts from cells harboring both FLAG-tagged Pop6 and cMyc-tagged Lsm4 were first subjected to IP with either anti-FLAG antibody to immunoprecipitate Pop6 or with anti-V5 antibody as a negative control. The resulting supernatants from these IPs were then subjected to a second round of immunoprecipitation with anti–cMyc antibody to immunoprecipitate the fraction of telomerase that was not associated with Pop6 and hence remained in the supernatant of the first IP. Prior work had shown that Lsm4 was associated quantitatively with active telomerase (Tang et al, 2012). Western blot analysis confirmed that the initial FLAG IP efficiently depleted FLAG–Pop6 from the IP supernatant (Fig. 2B). Most telomerase activity was associated with the Pop6 IP, whereas the subsequent Lsm4 IP contained only approximately 10% of telomerase activity, consistent with Pop6 associating with the majority of active enzyme (Fig. 2C, compare lanes 3 and 4 to lanes 7 and 8).

**Figure EV1:**
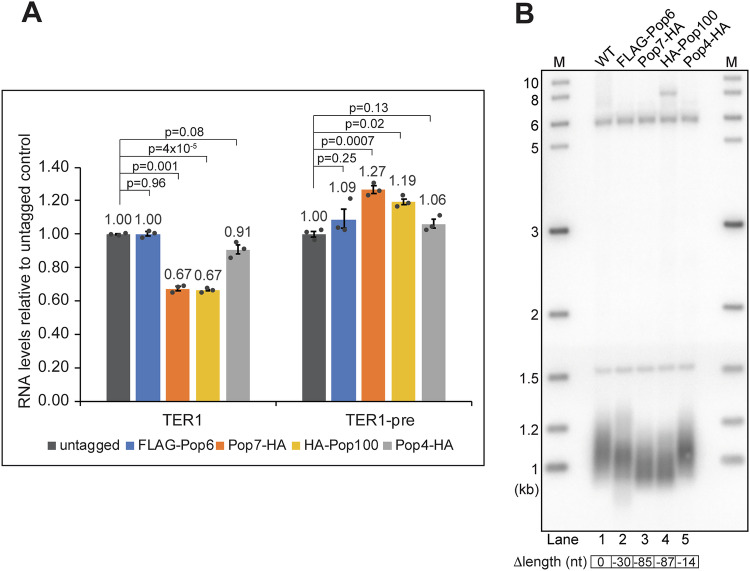
Effect of tagging Pop6, Pop7, Pop100, and Pop4 on telomere maintenance. (**A**) RT–qPCR quantification of TER1 RNA levels in strains expressing tagged Pop proteins under their endogenous promoters. Bars represent mean values (±SEM, *n* = 3) relative to the untagged control, normalized to reference genes. Statistical significance was assessed using unpaired *t* tests (*n* = 3). (**B**) Telomere length analysis by Southern blot in strains expressing tagged versions of Pop6, Pop7, Pop100, or Pop4. A probe against telomeric repeats was used for hybridization. ∆length indicates the difference in fragment length comparing each lane to lane 1 (WT).

In contrast, although Pop7–HA was efficiently depleted by the anti-HA IP (Fig. 2D), a larger amount (~48%) of telomerase activity was recovered in the Lsm4 IP, indicating that a smaller fraction of active telomerase is associated with Pop7 or that the complex is less stable under our assay conditions (Fig. 2E, compare lanes 3 and 4 to lanes 7 and 8). An even weaker association was observed for Pop100. Although the HA antibody immunoprecipitated >90% of HA–Pop100 (Fig. 2F), only ~17% of telomerase activity was associated with the HA IP (Fig. 2G). These differences may reflect reduced association of Pop7 and Pop100 with active telomerase. Alternatively, the presence of epitope tags may partially impair protein function and favor dissociation from TER1. Consistent with the latter possibility, RT–qPCR analysis revealed decreased steady-state levels of TER1 in Pop7–HA and HA–Pop100 strains compared to the untagged wild-type control (Fig. EV1A). In addition, telomere length analysis revealed a reduction in telomere lengths relative to wild-type by ~85nt in strains expressing HA-tagged Pop7 or Pop100 (Fig. EV1B).

### Pop6, Pop7, and Pop100 bind to the core region of TER1

**Figure 3:**
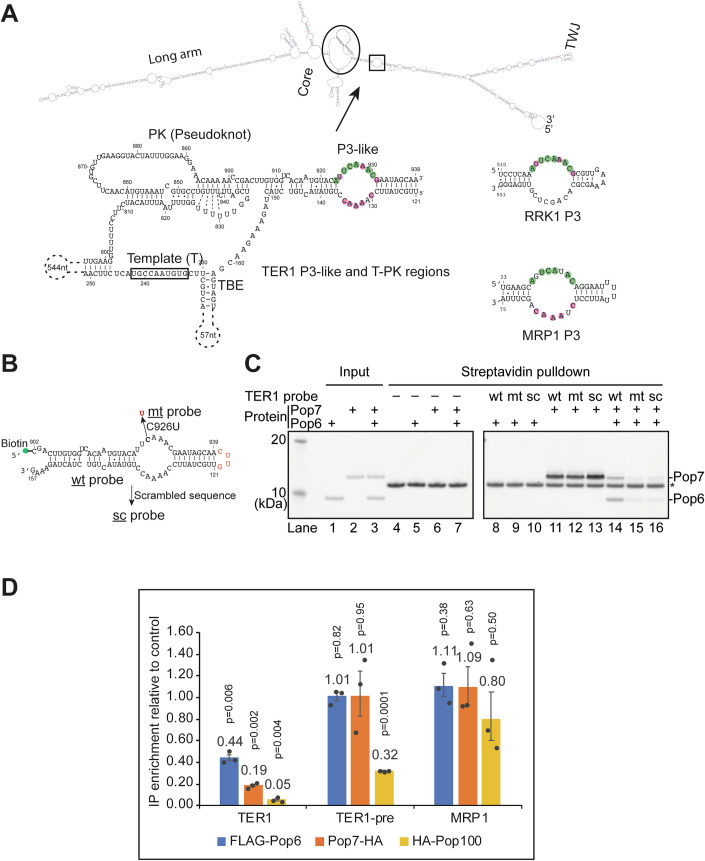
A putative Pop6/Pop7 binding site is located in the core region of TER1 RNA. (**A**) Predicted secondary structure of full-length TER1, with the region spanning nucleotides 802–907 replaced by the conserved pseudoknot structure predicted in (Qi et al, 2013). The Template–Pseudoknot (T-PK) region is highlighted with a circle, and the putative P3-like loop is shown with a square. Bottom left: zoom-in of the P3-like and T-PK regions, with a potential triple-helix structure indicated (dashed lines). Bottom right: P3 domains of RRK1 and MRP1 aligned to TER1; identical sequences are shaded green (all three RNAs) or magenta (TER1 + one homolog). (**B**) Schematic of biotinylated RNA probes used in pulldown assays. The wt probe includes TER1 nts 902–939 and 121–157 connected by a CUUG linker (orange). The mt probe contains a C926U mutation; the sc probe has a scrambled sequence. (**C**) Pulldown of recombinant Pop6, Pop7, or both by Streptavidin beads coated with TER1 RNA probes. Input and pulldown samples were analyzed by SDS-PAGE and Coomassie staining. Asterisk indicates monomeric Streptavidin, which is eluted from the beads under denaturing conditions in all samples. (**D**) The TER1 C926U mutation reduces binding of Pop6, Pop7, and Pop100. Extracts expressing WT or C926U-mutant TER1 with FLAG–Pop6, Pop7-HA, or HA-Pop100 were immunoprecipitated using tag-specific antibodies. RNA recovery from the C926U mutant was quantified by RT–qPCR, normalized to input, and expressed relative to the association with WT TER1. Bars show mean ± SEM (*n* = 3). Statistical significance was assessed using unpaired *t* tests (*n* = 3) comparing C926U-mutant samples to corresponding WT TER1 samples, and the respective *P* values are shown above each bar. Source data are available online for this figure.

In *S. cerevisiae*, the binding motif for Pop6/Pop7 is well conserved between TLC1, NME1, and RPR1 (Lemieux et al, 2016). To identify a potential binding site for Pop6/Pop7 in *S. pombe* TER1, we searched for sequence and structural similarities to the P3 domain of RRK1 (RNA subunit of RNase P in *S. pombe*) and MRP1 (RNA subunit of RNase MRP). Strikingly, nucleotides 923–931 of TER1 are identical in sequence to the 5′ arm of the loop in the P3 domain of RRK1 (positions 517–525), the key region for Pop6/Pop7 contacts (Fig. 3A). RNA secondary structure predictions of full-length TER1 using Mfold with default parameters and no constraints (Zuker, 2003), consistently placed this region in an internal loop for the six lowest free energy structures. In all six structure predictions, nucleotides 129–137 form the 5′ arm of this loop adjacent to the predicted pseudoknot region of TER1 (Qi et al, 2013). Nucleotides 923–931 form the corresponding 3’ arm (Fig. 3A).

To test whether this region corresponds to the Pop6/Pop7 binding site, we designed a biotin-labeled RNA probe consisting of TER1 nucleotide positions 902–939 and 121–157, connected by a CUUG tetraloop (Fig. 3B). Mfold prediction confirmed that this probe adopts the same secondary structure as predicted for the corresponding region in full-length TER1. To assess binding specificity, we introduced a single-nucleotide substitution at position 926, corresponding to a key site for Pop6/7 interaction with NME1 RNA in *S. cerevisiae* (Perederina et al, 2007; Perederina et al, 2010). This single-nucleotide change (mt probe) did not alter the predicted structure of the RNA. In addition, a scrambled RNA sequence was used as an additional control for binding specificity (sc probe).

Streptavidin beads without RNA did not retain Pop6, Pop7, or an equimolar mixture of both proteins, confirming that non-specific binding to the beads is negligible under the conditions of the assay (Fig. 3C, lanes 4–7). When the streptavidin beads were pre-coated with RNA, neither the wt nor the mt or sc probe retained Pop6 alone (Fig. 3C, lanes 8–10). In contrast, Pop7 was efficiently recovered with all three probes, suggesting that Pop7 interacts with RNA in a non-specific manner in the absence of Pop6 (Fig. 3C, lanes 11–13). This observation is consistent with the AlphaFold-predicted structure of Pop7, revealing a prominent positively charged surface, which would favor electrostatic interactions with negatively charged RNA (Appendix Fig. S4A). In contrast, Pop6 lacks a prominent surface charge (Appendix Fig. S4B). Incubation of the wt RNA probe with both Pop6 and Pop7 resulted in the retention of approximately equal amounts of both proteins on the beads (Fig. 3C, lane 14). In contrast, the single-nucleotide mutation (C926U) and the scrambled control probe retained approximately fivefold less protein, consistent with sequence-specific recognition of the P3-like loop in TER1 by a heterodimer of Pop6 and Pop7 (Fig. 3C, lanes 15 and 16).

**Figure EV2:**
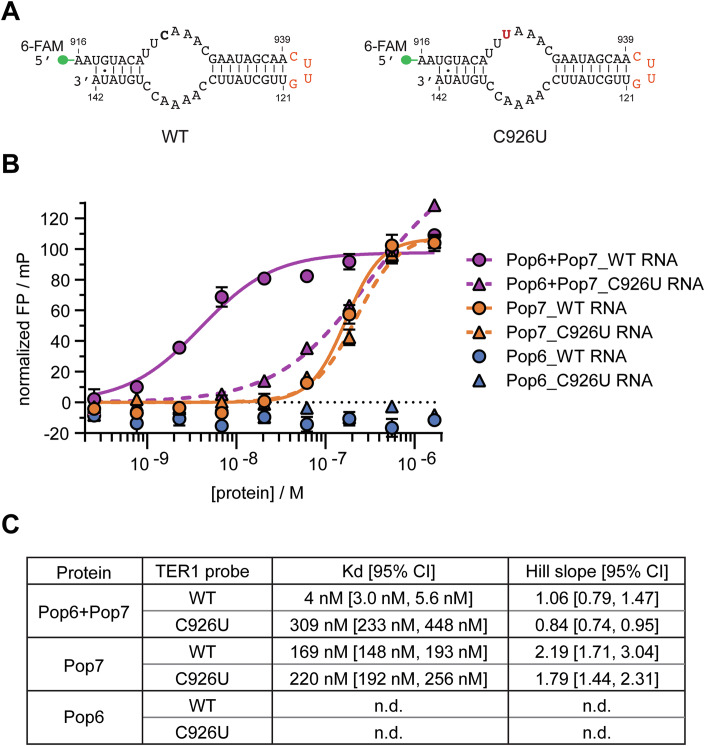
Fluorescence polarization measurements of Pop6, Pop7, or the complex binding to the TER1 P3-like domain in vitro. (**A**) Schematic of 6-FAM-labeled TER1 P3-like domain probes used in the fluorescence polarization assays. The WT probe includes TER1 nts 916–939 and 121–142 connected by a CUUG linker (orange). The C926U-mutant probe contains a C926U mutation (red). (**B**) Fluorescence polarization (FP) assays measuring binding of 6-FAM-labeled RNA by various amounts of Pop6, Pop7, or a 1:1 mixture of both proteins. Mean values from triplicates including error bars representing standard deviations are shown for each measurement. (**C**) Dissociation constant (Kd) and Hill slope derived from nonlinear regression fits of the FP measurements with the upper and lower limits of 95% confidence interval (CI) indicated in the brackets.

To further characterize the interactions with the P3-like domain, 6-carboxyfluorescein (6-FAM) labeled RNA substrates suitable for fluorescence polarization (FP) assays were synthesized (Fig. EV2A). Titrations of Pop6, Pop7, and both proteins in equimolar ratios were incubated with each RNA, and fluorescence polarization was measured (Fig. EV2B). The Pop6-Pop7 complex bound WT RNA with a Kd of 4 nM, while the C926U mutation decreased binding affinity by ~80-fold (Fig. EV2C), confirming the sequence-specific interaction of Pop6/Pop7 with the specific RNA motif and the importance of cytosine 926. A hill slope of close to 1 indicated a 1:1 stoichiometry of protein complex and RNA for the Pop6/Pop7 heterodimer. In contrast, the Pop7 protein alone bound to WT and C926U RNA with similar Kd (169 nM and 220 nM, respectively) and a hill slope of close to 2 (Fig. EV2C), indicating cooperative binding of two protein subunits. Pop6 alone showed no binding to either RNA (Fig. EV2B).

To test the importance of this interaction in vivo, we introduced the C926U mutation into the context of full-length TER1. The point mutation reduced the association of Pop6/Pop7 with mature TER1 by twofold to fivefold (Fig. 3D). In addition, the mutation reduced the binding of Pop100 by 20-fold. While Pop100 binding to the TER1 precursor was also reduced by the C926U mutation, Pop6 and Pop7 association with the precursor form were apparently unaffected (Fig. 3D). As structure predictions indicated that the sequence beyond the mature 3’ end folds largely independently without impacting the folding of sequences present in the mature form (Appendix Fig. S5), it must be considered that Pop6/Pop7 make additional contacts with RNA sequences that are only present in the precursor or interact with proteins that are specifically associated with the precursor form.

### TER1 P3-like loop mutants severely impair telomere maintenance

**Figure EV3:**
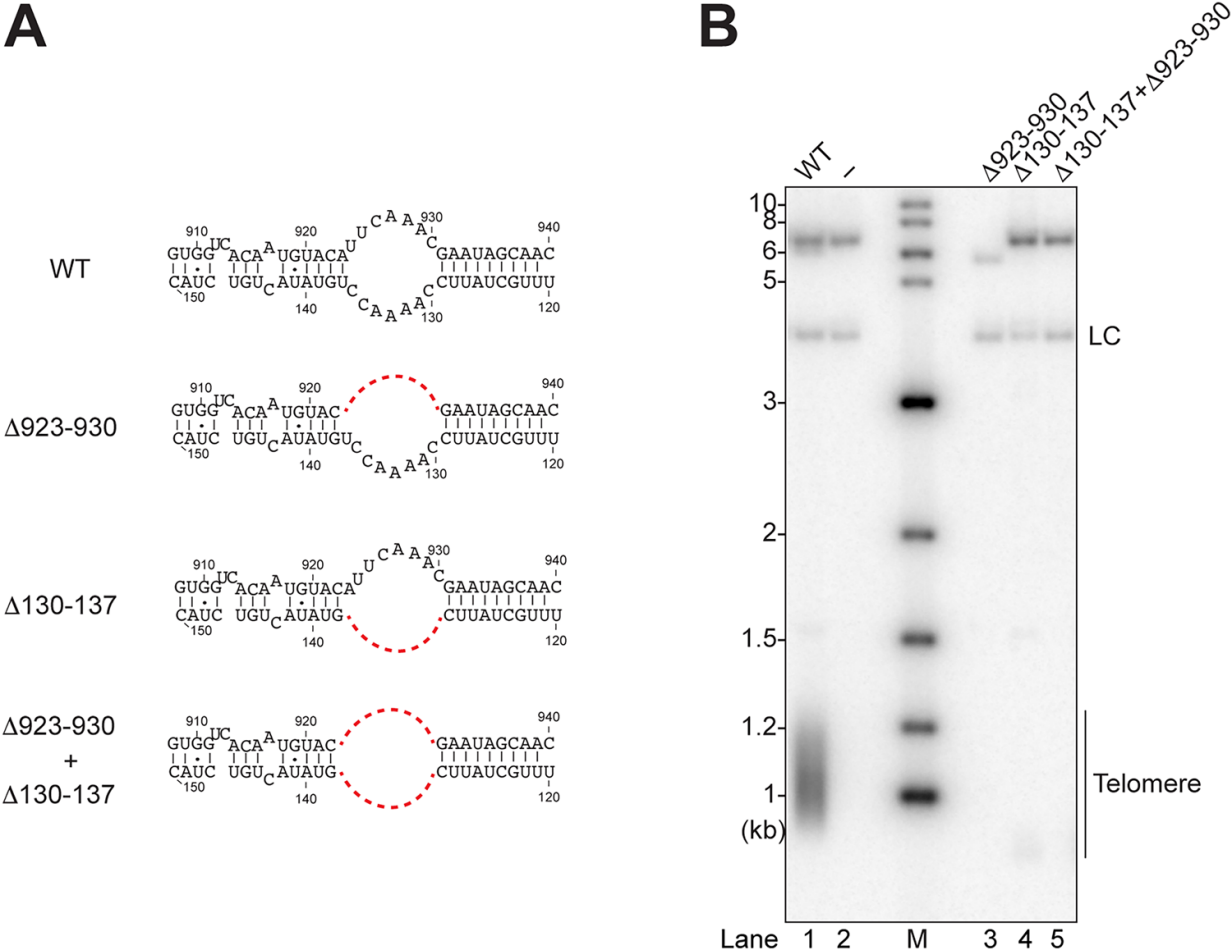
Telomere length analysis of P3 loop deletion mutants. (**A**) Schematic of P3 loop deletion mutants. Red dashed lines indicate the deleted RNA sequences. (**B**) Telomere length analysis of the deletion mutants shown in (**A**), expressed from a plasmid in *ter1*∆ cells and restreaked five times (~110 generations). Southern blot was performed using a probe against telomeric repeats and *rad16⁺* as a loading control (LC). M: size ladder, with molecular weight markers indicated on the left.

**Figure 4:**
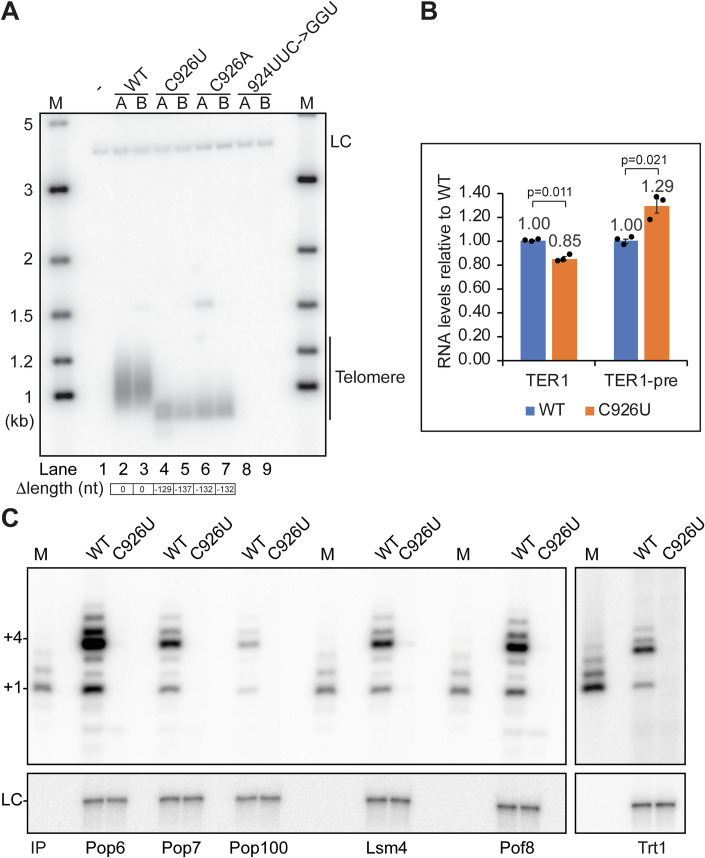
TER1 P3 mutation impairs telomere maintenance. (**A**) Telomere length analysis by Southern blot in *ter1*∆ cells expressing WT or P3-mutant TER1 from a plasmid and restreaked five times (~110 generations). A probe against telomeric repeats was used, with *rad16⁺* as a loading control. ∆length indicates the difference in fragment length comparing each lane to lane 2 (WT A isolate). (**B**) RT–qPCR quantification of total TER1 RNA in WT and C926U-mutant backgrounds. Bars show mean ± SEM (*n* = 3) after normalization to reference genes. Statistical significance was assessed using unpaired *t* tests between WT and the C926U mutant (*n* = 3). (**C**) Telomerase activity assay of WT and C926U TER1 mutants following IP of FLAG–Pop6, Pop7-HA, HA-Pop100, Lsm4-cMyc, FLAG-Pof8, and Trt1-V5. Ladder and loading control as in Fig. 2A. Source data are available online for this figure.

The identification of the Pop-binding site within TER1 RNA enabled us to further investigate the functional significance of this region for telomerase activity. We first deleted either the 5′ or 3′ arm of the loop, or both arms together (Fig. EV3A). Telomere length analysis revealed that deletion of the 5’ arm led to severe telomere shortening, and deletion of 3’ arm or both arms led to complete telomere loss over 110 generations, confirming the essential role of this RNA element in telomere maintenance (Fig. EV3B). Even a single-nucleotide substitution at position C926 caused telomere shortening of ~130 nt (Fig. 4A, lanes 4–7). Further mutation of two adjacent nucleotides (924UUC to GGU) resulted in complete telomere loss after 110 generations (Fig. 4A, lanes 8 and 9).

To assess whether these mutations affected TER1 RNA levels, we performed RT–qPCR on total RNA isolated from cells expressing either wild-type or C926U-mutant TER1. The analysis revealed a mild reduction in steady-state levels of mature TER1 and a modest increase in the precursor form (Fig. 4B). Despite the relatively minor impact on RNA stability, telomerase activity assays revealed a marked reduction in activity associated with Pop6, Pop7, and Pop100 (Fig. 4C).

To determine whether this reduction in activity was merely due to impaired Pop protein binding, we also assessed telomerase activity associated with Lsm4 and Pof8, two constitutive components of the active complex (Paez-Moscoso et al, 2018; Tang et al, 2012), and Trt1, the catalytic subunit. All immunoprecipitates displayed a comparable reduction in telomerase activity (Fig. 4C). These findings demonstrate that the C926U mutation directly impairs telomerase function.

### Pop6, Pop7, and Pop100 promote telomerase assembly

**Figure 5:**
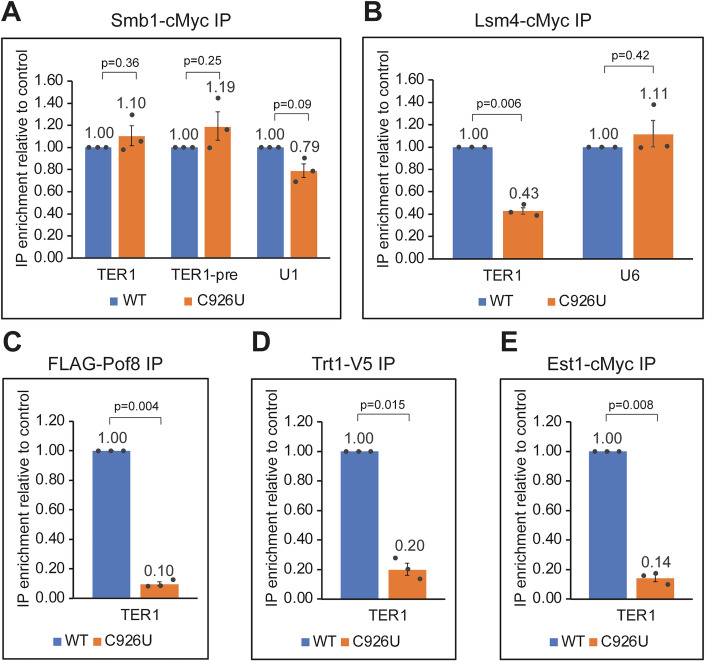
Compromised Pop6, Pop7, and Pop100 binding affects telomerase holoenzyme assembly. (**A**) Smb1 binding to TER1 is not affected by the C926U mutation. Bars show mean RNA enrichment ( ± SEM, *n* = 3) in the mutant relative to WT, normalized to input, following Smb1-cMyc IP using anti-c-Myc antibody and RT–qPCR. U1 served as a positive control target for the Sm complex. (**B**) Lsm4 binding to TER1 is reduced by the C926U mutation. RNA enrichment by Lsm4-cMyc IP using anti-c-Myc antibody was quantified as in (**A**). U6 served as a positive control for Lsm2–8 binding. (**C**) Pof8 binding to TER1 is strongly reduced by the C926U mutation. RNA enrichment by FLAG-Pof8 IP using anti-FLAG antibody was analyzed as in (**A**). (**D**) Trt1 binding to TER1 is strongly reduced by the C926U mutation. RNA enrichment by Trt1-V5 IP using anti-V5 antibody was analyzed as in (**A**). (**E**) Est1 binding to TER1 is strongly reduced by the C926U mutation. RNA enrichment by Est1-cMyc IP using anti-c-Myc antibody was analyzed as in (**A**). Statistical significance assessments for (**A**–**E**): unpaired *t* tests comparing C926U mutant to WT (*n* = 3). Source data are available online for this figure.

We next examined whether the C926U mutation affects the recruitment of individual telomerase subunits. Binding of Smb1, a component of the Sm complex that associates with the TER1 precursor, was unaffected by the mutation, indicating that Sm complex binding occurs independently of Pop proteins (Fig. 5A). In contrast, association of Lsm4, a subunit of the Lsm2–8 complex that binds to the 3′ end of TER1 following spliceosomal cleavage, was reduced to ~43% (Fig. 5B). A similar decrease to ~40% was observed for Lsm8 (Appendix Fig. S6). More pronounced effects were observed for other components of the telomerase holoenzyme. Binding of Pof8 was reduced to approximately 10% of wild-type levels (Fig. 5C). Similarly, association of the catalytic subunit Trt1 with TER1 was reduced to ~20%, confirming that this single-nucleotide mutation disrupts assembly of the functional enzyme complex (Fig. 5D). Interestingly, Est1 binding was also reduced to ~14% in the mutant background (Fig. 5E), despite previous findings indicating that Trt1 and Est1 bind TER1 independently of one another (Leonardi et al, 2008; Webb and Zakian, 2008).

The observation that loss of Pop binding profoundly impairs the recruitment of multiple core subunits supports a model in which Pop6, Pop7, and Pop100 contribute to shaping the RNA scaffold to facilitate efficient telomerase assembly.

### Pop binding coincides with structural changes in the T-PK region

To investigate in vivo structural changes in TER1 RNA, we performed in-cell selective 2′-hydroxyl acylation analyzed by primer extension and mutational profiling (SHAPE-MaP) (Smola et al, 2015; Smola and Weeks, 2018). The SHAPE reagent 1-methyl-7-nitroisatoic anhydride (1M7) modifies the 2′-hydroxyl group of ribose in flexible, single-stranded regions of RNA, leading to misincorporation during reverse transcription at modified sites. The resulting mutations are quantified via high-throughput sequencing, and the reactivity is calculated for each nucleotide to infer RNA structure. High reactivities correlate well with single-stranded regions, and low reactivities are indicative of base-paired regions.

**Figure EV4:**
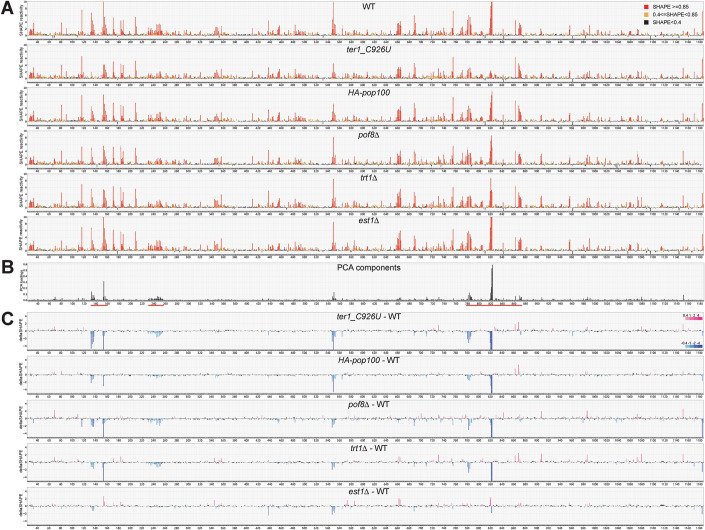
In-cell SHAPE-MaP profiles of TER1 RNA. (**A**) In-cell SHAPE-MaP analysis of TER1 in WT, *ter1_C926U*, *HA-pop100*, *pof8*∆, *trt1*∆, and *est1*∆ strains. Bars represent mean SHAPE reactivities ( ± SEM, *n* = 3), with a maximum display value of 10, across nucleotide positions 22–1186 (primer regions excluded). (**B**) PCA loadings for each nucleotide from the principal component analysis (PCA) of SHAPE data shown in (**A**). Underlined regions correspond to the P3-like and T–PK domains of TER1. (**C**) deltaSHAPE profiles comparing average SHAPE reactivities of *ter1_C926U*, *HA-pop100*, *pof8*∆, *trt1*∆, and *est1*∆strains to WT (mutant minus WT). Nucleotides with deltaSHAPE > 0.4 are shown in pink, < –0.4 in blue, and between –0.4 and 0.4 in black.

To assess the structural impact of the point mutation, we applied in-cell SHAPE-MaP to full-length TER1 RNA from strains expressing either wild-type or C926U-mutant TER1 (Fig. EV4A). Furthermore, a strain harboring HA–Pop100 was included in the analysis due to its hypomorphic phenotype, indicating that the epitope tag partially compromised Pop100 function, reducing TER1 levels to ~67% and shortening telomeres (Fig. EV1). SHAPE profiles were also generated for TER1 from strains carrying deletions for either *pof8*, *trt1*, or *est1*. These additional samples were included as associations of Pof8, Trt1, and Est1 to TER1 appeared to be severely impaired by the C926U mutation (Fig. 5C–E), and we wanted to compare the structural changes caused by these gene deletions with the effect of the C926U mutation.

**Figure 6:**
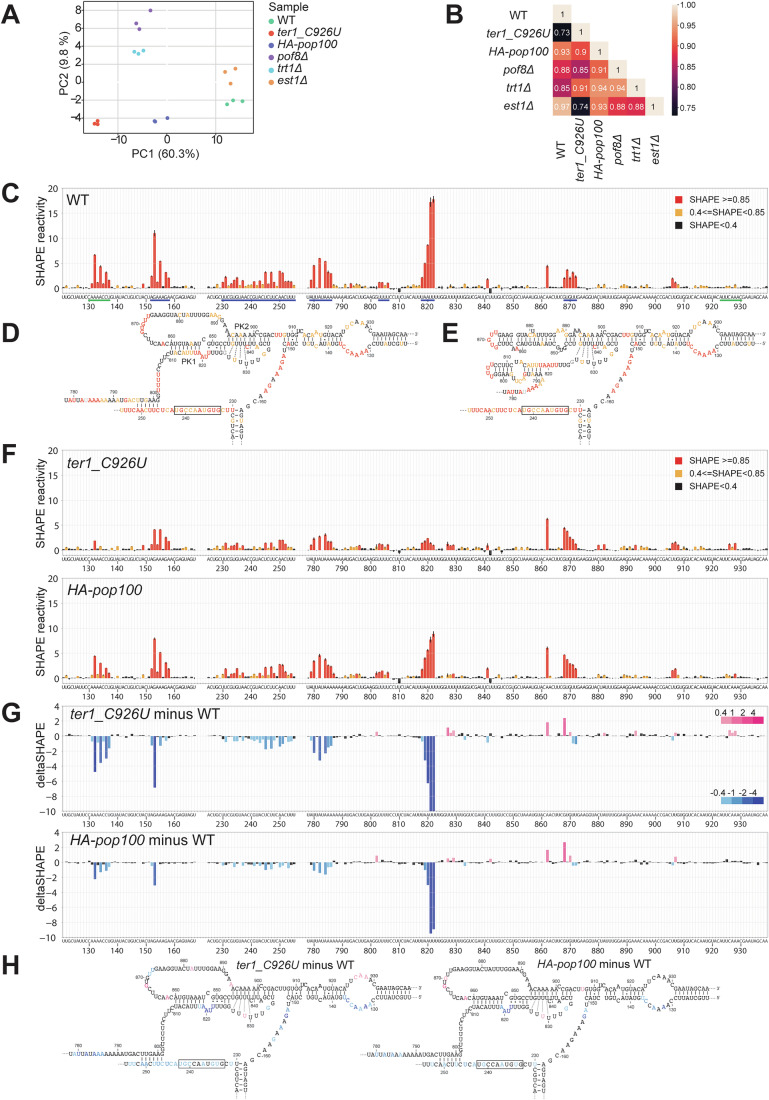
SHAPE-MaP profiles and structural changes in the P3–template–pseudoknot (P3–T–PK) region of TER1. (**A**) Principal component analysis (PCA) of SHAPE reactivities across TER1 in WT, *ter1_C926*, *HA-pop100*, *pof8*∆, *trt1*∆, and *est1*∆ samples, based on biological triplicates. (**B**) Pearson correlation matrix of per-nucleotide average SHAPE reactivities across the same sample set. (**C**) Mean SHAPE reactivity profiles ( ± SEM, *n* = 3) for the P3-like and T-PK regions of TER1 (nucleotide positions 121–167, 225–255, and 778–939) in WT cells. Red, orange, and black correspond to high, moderate, and low reactivities, respectively. Green lines under the trace indicate the P3-like loop nucleotides; blue lines mark highly reactive regions in the T-PK region. (**D**) Nucleotides in the P3-like and T-PK regions of TER1 colored according to SHAPE reactivities in WT cells, overlaid on the predicted secondary structure. (**E**) Possible alternative base pairings in the T-PK region indicated by SHAPE reactivities. (**F**) Mean SHAPE reactivity profiles ( ± SEM, *n* = 3) for the P3-like and T-PK regions of TER1 (nucleotide positions 121–167, 225–255, and 778–939) in *ter1_C926U* and *HA-pop100* strains. (**G**) deltaSHAPE profiles (mutant minus WT) comparing *ter1_C926U* and *HA-pop100*, to WT for the P3-like and T-PK regions. Nucleotides with deltaSHAPE >0.4 are shown in pink; deltaSHAPE < -0.4 in blue; and -0.4 ≤ deltaSHAPE ≤0.4 in black. (**H**) deltaSHAPE from (**G**) mapped onto the predicted TER1 structure. Only nucleotides with statistically significant changes (*P* < 0.05, unpaired *t* test, *n* = 3) are colored (pink or blue); non-significant positions (*P* ≥ 0.05) are shown in black. Source data are available online for this figure.

Principal component analysis (PCA) of SHAPE reactivities confirmed high reproducibility and consistent structural patterns across biological replicates (Fig. 6A). Notably, the C926U mutation had a greater impact on global SHAPE reactivity than deletions of any single protein subunit, as determined by PCA and Pearson correlation analyses (Fig. 6A,B). Analysis of PCA loadings across nucleotide positions showed that most of the RNA structure was unaffected, with structural differences concentrated in a few discrete regions (underlined in red in Fig. EV4B). Even though the affected regions were distant from each other in primary sequence space, they mostly map near the P3-like loop and template–pseudoknot (T–PK) region of TER1 in the structure model (Appendix Fig. S7). SHAPE difference maps (deltaSHAPE) for each mutant compared to wild-type closely mirrored the nucleotide-specific differences indicated by PCA loadings, further supporting the conclusion that Pop binding is a key determinant for the folding of the core region, comprised of the P3-like loop, the template, and the pseudoknot (Fig. EV4C).

Further inspection of SHAPE profiles across this region revealed several highly reactive sites in the wild-type (WT) sample indicative of exposed single-strandedness (red bars in Fig. 6C). For the P3-like loop (green underlined regions), the 5′ arm (nucleotides 131–137) was highly reactive, whereas the 3′ arm (nt 923–930) and flanking regions showed only mild reactivity (Fig. 6C,D). These features closely resemble the SHAPE profile of the P3 loop in MRP1, where the 3′ arm (nt 63–69) is highly reactive, and the opposing arm (nt 39–46) remains unreactive (Appendix Fig. S8A, underlined in green, S8B). This asymmetric reactivity pattern is consistent with the binding of Pop6, Pop7, and Pop100 to one arm of the loop, thereby shielding it and leaving the opposite arm solvent exposed.

The WT sample also exhibited distinct SHAPE reactivities in the T-PK region (blue underlined regions in Fig. 6C), including the linker between the TBE and P3-like loop, the template region and its downstream sequences extending to nucleotide 255, as well as portions of pseudoknot stem 1 (PK1) (Fig. 6D). In addition, two prominent stretches of high reactivity were observed at positions 803–806 and 868–872. In both cases, the flanking sequences show high complementarity and low reactivity, supporting the potential formation of an alternative structure (Fig. 6E).

These findings align with a recent phylogenetics-based study of TER1 secondary structure that proposed the existence of alternative or intermediate folding within the PK1 region (McMurdie et al, 2026). Supporting this model, five nucleotides within the predicted PK1 stem (nt 818–822) were strongly reactive (Fig. 6C,E). In contrast, the 3′ arm of the predicted stem lacked SHAPE reactivity. Notably, the lowest free-energy structure predicted by Mfold also adopted this alternative pairing configuration (Appendix Fig. S9A,B, structure 1).

Comparison of the *ter1_C926U* mutant to wild-type revealed marked alterations in SHAPE reactivity across the P3-like loop and T-PK region of TER1 (Fig. 6F,G, top panels). The mutation significantly decreased reactivity in the 5′ arm of the loop and modestly increased reactivity in the 3′ arm, consistent with loss of Pop6/Pop7 binding (Fig. 6G, top panel; Fig. 6H, left). To confirm that these changes in reactivity are a specific consequence of the C926U mutation in TER1, we examined the SHAPE reactivity of the P3 loop in MRP1 from the two strains as a negative control. As expected, SHAPE reactivity of the P3 loop in MRP1 was unaffected by the C926U mutation in TER1, underscoring the specificity of the structural change in TER1 (Appendix Fig. S10A,B).

**Figure EV5:**
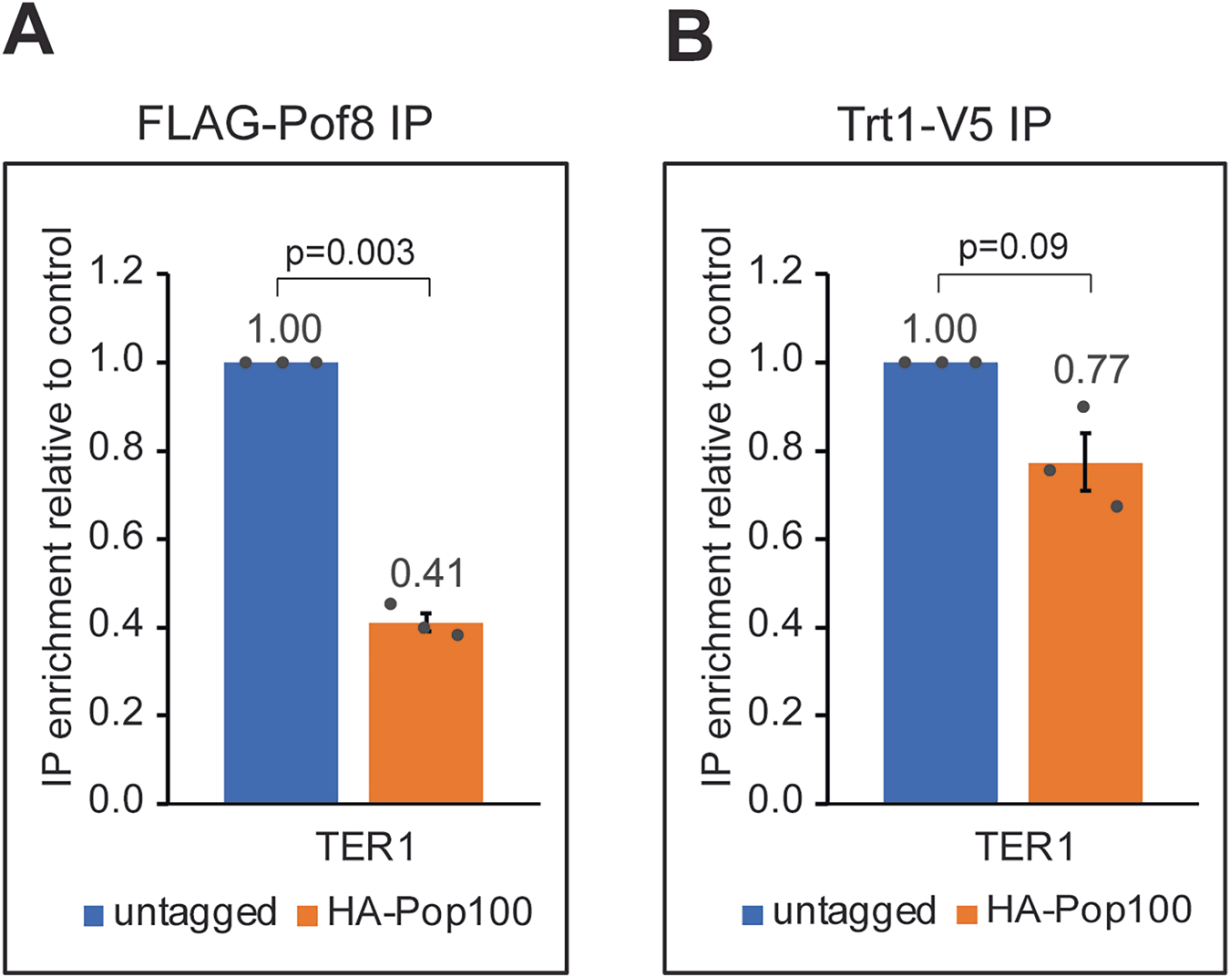
Effect of HA-tagged Pop100 on Pof8 and Trt1 binding to TER1. (**A**) RT–qPCR analysis of RNA recovered from FLAG-Pof8 IP from extracts with untagged- and HA-tagged Pop100. Bars represent mean enrichment ( ± SEM, *n* = 3) relative to untagged-Pop100 and normalized to input. Statistical analysis: unpaired *t* tests (*n* = 3). (**B**) RT–qPCR analysis of RNA recovered from Trt1-V5 IP from extracts with untagged- and HA-tagged Pop100 as in (**A**). Statistical analysis: unpaired *t* tests (*n* = 3).

The C926U mutation also caused a pronounced reduction in SHAPE reactivity within the linker and template regions, as well as in the 5′ side of PK1 (Fig. 6G, top panel; Fig. 6H, left). These results suggest that loss of Pop protein binding disrupts the structural integrity of the entire T-PK core. This was also supported by the reactivity pattern in the hypomorphic HA-Pop100 strain, where a similar but less pronounced pattern was observed (Fig. 6F,G, bottom panel, Fig. 6H, right). Furthermore, HA-Pop100 also reduced Pof8 and Trt1 association with TER1 in immunoprecipitations (Fig. EV5A,B), but to a lesser extent compared to the TER1 C926U mutant (Fig. 5C,D).

The in-cell SHAPE profile provides in vivo information of the RNA structure; however, it reflects the average reactivity in a potentially heterogenous population of molecules. Indeed, structure prediction analyses of the TER1 core produced a set of heterogeneous folding outcomes with comparable free energy values (Appendix Fig. 9B). The top three predicted models all displayed partial pairing involving the single-stranded linker upstream of the TBE, the template region, and the PK1 stem, features that are consistent with the SHAPE changes observed in the C926U mutant and in HA-Pop100 strain relative to wild-type (Appendix Fig. 9B).

**Figure 7:**
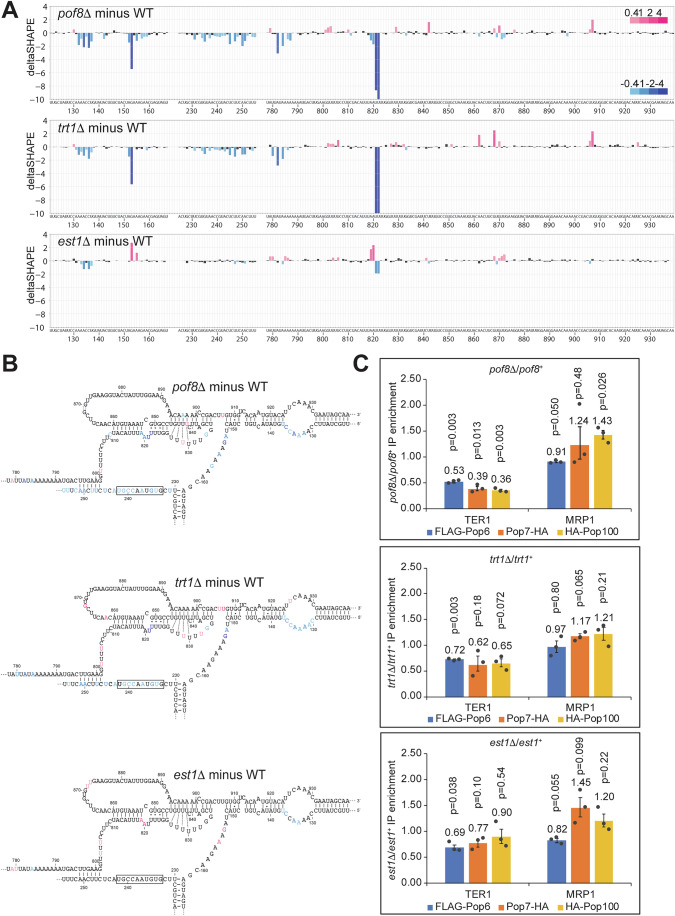
Coordinated binding of Pop6, Pop7, Pop100, and Pof8. (**A**) deltaSHAPE profiles (mutant minus WT) comparing *pof8*∆*, trt1*∆ and *est1*∆, to WT for the P3-like and T-PK regions. Nucleotides with deltaSHAPE >0.4 are shown in pink; deltaSHAPE < −0.4 in blue; and −0.4 ≤ deltaSHAPE ≤ 0.4 in black. (**B**) deltaSHAPE from (**A**) mapped onto the predicted TER1 structure. Only nucleotides with statistically significant changes (*P* < 0.05, unpaired *t* test, *n* = 3) are colored (pink or blue); non-significant positions (*P *≥ 0.05) are shown in black. (**C**) Effect of *pof8*∆*, trt1*∆ and *est1*∆ on Pop6, Pop7, and Pop100 binding to TER1. RT–qPCR analysis of RNA recovered from FLAG–Pop6, Pop7-HA, or HA-Pop100 IPs in the presence or absence of Pof8, Trt1, and Est1. Bars represent mean enrichment ( ± SEM, *n* = 3) in the absence relative to in the presence of the protein, normalized to input. Statistical significance was assessed using unpaired *t* tests (*n* = 3) comparing *pof8*∆*, trt1*∆ and *est1*∆, to the control (with *pof8*^*+*^, *trt1*^*+*^, and *est1*^*+*^). Source data are available online for this figure.

These findings further support a model in which Pop6, Pop7, and Pop100 binding is important for the correct folding of the core T-PK region of telomerase RNA, thereby facilitating assembly with other protein subunits. Previous studies showed that Pof8 interacts with the pseudoknot (PK) domain, and Trt1 is likely to directly engage with the T-PK region (Hu et al, 2020; Podlevsky and Chen, 2016). Consistent with these interactions, deletion of either *pof8* or *trt1* produced structural changes in these regions that were intermediate between those observed in the *ter1_C926U* and *HA–Pop100* strains (Fig. 7A,B). In contrast, the T-PK region in the *est1*∆ strain exhibited only minimal changes, suggesting that Est1 plays a different or less direct role in shaping the RNA structure (Fig. 7A,B).

In contrast to the profound structural changes observed in the P3-like and T-PK regions, another essential element in telomerase RNA, the conserved three-way junction (TWJ; nt 1035–1096), remained largely unaffected in all mutant backgrounds (Appendix Fig. S11). Only a small number of nucleotides exhibited increased SHAPE reactivity in the *trt1*∆ and *ter1_C926U* mutant backgrounds, consistent with the loss of Trt1 interaction (Appendix Fig. S11C,F).

### Pof8 reciprocally stabilizes Pop6, Pop7, and Pop100 binding

In addition to producing similar structural changes in the T-PK region, the *pof8*∆ and *trt1*∆ strains also exhibited reduced SHAPE reactivity in the 5′ arm of the P3-like loop (Fig. 7A,B), indicative of diminished Pop6, Pop7, and Pop100 binding. To directly assess whether Pof8 influences Pop association with TER1, we performed RT–qPCR on RNA co-immunoprecipitated with Pop6, Pop7, or Pop100 in the presence or absence of *pof8*. Deletion of *pof8* reduced TER1 binding by approximately two- to three-fold for all three Pop proteins (Fig. 7C). Deletion of *trt1* also resulted in reduced association, though to a lesser extent, while deletion of *est1* had the mildest effect (Fig. 7C). Notably, the degree of reduction in Pop6/7/100 binding correlated with the extent of SHAPE reactivity change in the P3-like loop.

These findings indicate that Pof8 binding enhances Pop6/7/100 association with TER1 and vice versa, supporting a model of reciprocal stabilization. A similar, though weaker, cooperative relationship appears to exist between Pop6/7/100 and Trt1 or Est1, respectively.

## Discussion

Here, we identified three additional players in fission yeast telomerase biogenesis: Pop6, Pop7, and Pop100. These proteins are subunits of the conserved ribonucleases RNase P and RNase MRP. We show that Pop6/7/100 bind to TER1 and are incorporated into the active telomerase complex (Figs. 1D,E and 2). Specifically, they interact with a stem–loop–stem region in TER1 that resembles the P3 binding domain in MRP1 and RRK1 (Fig. 3). A single-nucleotide mutation in this P3-like loop in TER1 (C926U) reduces Pop protein binding (Fig. 3C,D), leading to pronounced telomere shortening and reduced telomerase activity (Fig. 4A,C). This phenotype is not due to diminished RNA levels, but rather to a defect in the assembly with additional telomerase components, including Pof8, Trt1, and Est1 (Fig. 5).

The involvement of these RNase P/MRP subunits in telomerase biogenesis was first observed in budding yeast (*S. cerevisiae*), where they promote Est1 and Est2 binding to TLC1 and associate with the active telomerase complex (Lemieux et al, 2016). While these findings parallel our results in fission yeast, the underlying mechanisms appear to differ. In budding yeast, Pop6/7/1 bind a P3-like domain near the distal end of stem IVc, adjacent to the Est1-binding site (Lemieux et al, 2016). Further analysis showed that even a 2 bp increase in the distance between these sites strongly impairs Est1 binding and moderately affects Est2 binding (Laterreur et al, 2018), suggesting a spatial requirement that may involve direct interactions between Pop proteins and Est1.

**Figure 8:**
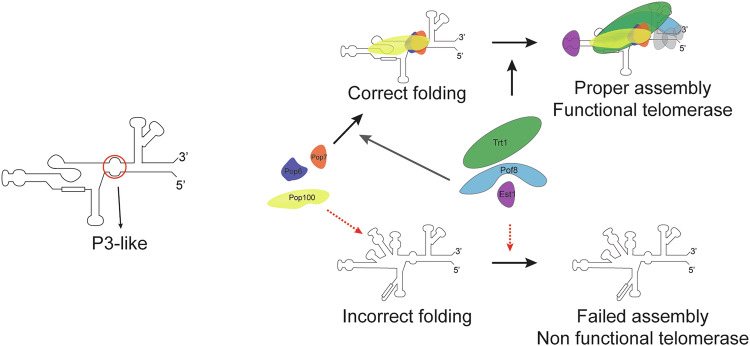
Proposed model for the role of Pop6, Pop7, and Pop100 in telomerase assembly. Early binding of Pop6, Pop7, and Pop100 to TER1 at the P3-like domain, which is close to the T-PK region, promotes correct folding of the core RNA region, enabling subsequent association of Pof8, Trt1, and Est1 and formation of a functional telomerase holoenzyme. In the absence of Pop6/Pop7/Pop100 binding, TER1 adopts alternative, nonfunctional structures that prevent downstream factor recruitment and lead to failed assembly. The binding of Pof8 and possibly Trt1 may further stabilize the RNA structure and reinforce Pop protein interactions.

Structural profiling by DMS-MaPseq in budding yeast revealed no global changes in TLC1 folding in Pop temperature-sensitive mutants, except in the P3-like loop (Garcia et al, 2020). In contrast, the P3-like loop in fission yeast TER1 resides within the core region, close to the pseudoknot (PK), and distant from the distal arm that harbors the Est1-binding site (Fig. 8). Mutation of this loop disrupts the binding of Pof8, Trt1, and Est1 (Fig. 5C–E). While Pof8 is required for Trt1 association, Est1 and Trt1 bind independently to TER1. Our SHAPE-MaP analysis reveals that the P3-like mutation alters the RNA structure not only at the P3-like site but also in the adjacent T-PK region (Fig. 6). Pof8 binds the PK domain and recruits Lsm2-8 to stabilize correctly folded TER1 (Hu et al, 2020), and the PK is also critical for telomerase activity, likely serving as a key binding site for Trt1 (Podlevsky and Chen, 2016). Together, these results suggest that Pop6/7/100 act in fission yeast telomerase biogenesis by facilitating correct folding of the TER1 RNA. Proper RNA folding is a prerequisite for Pof8, Trt1, and Est1 recruitment and formation of the active enzyme. Impaired Pop protein binding leads to structural defects in the core region of TER1, thereby disrupting the assembly of telomerase components and resulting in a nonfunctional complex (Fig. 8).

The PK domain of human telomerase RNA (hTR) is prone to folding into alternative conformations (Theimer et al, 2003). Overstabilization of one such structure reduces telomerase activity (Deshpande and Collins, 2018), yet eliminating this structural flexibility also impairs function, suggesting that the alternative conformation may have a biological function (Deshpande and Collins, 2018). Similar behavior has been observed in budding yeast and in the ciliate *Tetrahymena*, where the PK region of telomerase RNA adopts alternative conformations (Bhattacharyya and Blackburn, 1994; Cash and Feigon, 2017; Liu et al, 2012; Mihalusova et al, 2011). Our SHAPE-MaP data show that in wild-type TER1, the PK1 stem is not stably paired (Fig. 6D), possibly reflecting the presence of alternative structures in vivo. Consistent with this idea, disrupting PK1 pairing by mutating either arm does not shorten telomeres, whereas compensatory mutations that restore pairing do (McMurdie et al, 2026). Our SHAPE reactivity data agree largely with a previous study where SHAPE reactivity of the PK region was accessed for Trt1-cMyc associated TER1 (Hu et al, 2020). Interestingly, in this study, the 5’ arm of the PK1 stem had low reactivity while in ours it had medium to high reactivity. This could be explained by Trt1 associating preferentially with the fraction of TER1 that adopts the pseudoknot structure shown in Fig. 6D. However, the possibility that the difference in reactivity is related to the use of different SHAPE chemicals and read-outs cannot be excluded. Further deconvolution analysis of the PK region may reveal alternative structures in vivo and identify structure-specific binding preferences for different protein subunits.

In the Pop-binding mutant, SHAPE reactivities in the single-stranded linker preceding TBE, the template region, and part of PK1 are reduced, suggesting that these regions preferentially form base pairs (Fig. 6). These findings further support a role for Pop proteins in promoting the correct T-PK structure. In *S. cerevisiae*, Pop1 is known to contact multiple dispersed sites on RNase P and RNase MRP RNA and is thought to influence global RNA folding (Fagerlund et al, 2015; Garcia et al, 2020). Pop6 and Pop7, which bind specifically to the P3 region, serve as anchor points for Pop1. In *S. pombe*, Pop100 may similarly engage additional regions of TER1 to aid in folding.

In budding yeast, Pop6/7/1 bind TLC1 co-transcriptionally in the nucleus (Neumann et al, 2023), and temperature-sensitive mutations in *pop1* and *pop6* lead to TLC1 cytoplasmic retention and impaired nuclear re-import (Garcia et al, 2020). In fission yeast, the interaction of Pop6/7/100 with the TER1 precursor suggests that binding occurs early, prior to spliceosomal cleavage of the RNA (Fig. 1E). However, the dynamics of TER1 transport and subunit assembly across compartments remain unclear. It will be insightful to investigate how Pop proteins and the other components such as Pof8/Bmc1/Thc1 affect telomerase localization using cytological approaches in fission yeast.

Structural profiling revealed similar patterns of T-PK changes in *pof8*∆ and *trt1*∆ cells compared to the *ter1_C926U* mutant, although the effects were milder (Fig. 7A,B). Likewise, mild changes in the P3-like region were also seen in *pof8*∆ and *trt1*∆, raising the possibility that Pof8 and Trt1 may reciprocally influence Pop6/7/100 binding. In line with this, Pop6/7/100 binding to TER1 is strongly reduced in *pof8*∆ and mildly reduced in *trt1*∆ (Fig. 7C). Thus, while Pop binding is essential for Pof8 and Trt1 recruitment, the reverse may also hold true: Pof8 and Trt1 may help stabilize Pop binding, possibly by further stabilizing the RNA structure (Fig. 8).

Previous studies have highlighted the interplay between telomerase and various RNA processing machineries during biogenesis (Box et al, 2008; Collopy et al, 2018; Hu et al, 2020; Mennie et al, 2018; Paez-Moscoso et al, 2022; Paez-Moscoso et al, 2018; Porat et al, 2022; Tang et al, 2012). Our findings now place RNase P/MRP within this network of RNP interactions, with conserved roles across budding and fission yeast. Furthermore, knockdown of Pop1 in human breast cancer cells affects telomerase RNA levels and tumor progression (Zhu et al, 2023). These observations suggest that the involvement of RNase P/MRP components in telomerase biogenesis is evolutionarily ancient, although the underlying mechanisms may have diverged over time.

## Methods

**Table:** Reagents and tools table

Reagent/resource	Reference or source	Identifier or catalog number
**Experimental models**		
*S.pombe* strains	This study	Appendix Table S1
**Recombinant DNA**		
Plasmids	This study	Appendix Table S2
**Antibodies**		
Mouse anti-FLAG	Merck	F3165
Mouse anti-HA	Thermo Fisher	26183
Mouse anti-c-Myc	Merck	M4439
Mouse anti-V5	Thermo Fisher Scientific	R960-25
Mouse anti-FLAG-HRP	Merck	A8592
Mouse anti-V5-HRP	Thermo Fisher Scientific	R961-25
Rabbit anti-c-Myc	Santa Cruz Biotechnology	Sc-789
Rabbit anti-HA	Cell Signaling Technology	3724S
Goat anti-rabbit-HRP	Thermo Fisher Scientific	31460
**Oligonucleotides and other sequence-based reagents**		
Primers	This study	Appendix Table S3
RNA probes for in vitro pulldown and fluorescence polarization assays	This study	Appendix Table S3
**Chemicals, enzymes, and other reagents**		
Protein G Dynabeads	Thermo Fisher Scientific	10004D
RNasin Plus RNase inhibitor	Promega	N2611
RNA Clean & Concentrator25 Kit	Zymo Research	R1018
Proteinase K	Merck	P2308
SuperScript VILO cDNA Synthesis Kit	Thermo Fisher Scientific	11754250
Phenol:Chloroform:Isoamyl Alcohol 25:24:1	Merck	P3803
Chloroform: Isoamyl alcohol 24:1	Merck	25666
PerfeCTa SYBR Green FastMix Low ROX	Quantabio	733-1390
RNase H	New England Biolabs	M0297S
High Prime	Roche	11 585 592 001
T4 polynucleotide kinase	New England Biolabs	M0201S
[α-³²P]-dCTP	Perkin Elmer	NEG513Z500UC
[γ-³²P]-ATP	Perkin Elmer	NEG035C001MC
cOmplete EDTA-free protease inhibitor cocktail	Merck	4693132001
RNase A	Thermo Fisher Scientific	EN0531
4–12% NuPAGE Bis-Tris gel	Thermo Fisher Scientific	NP0321
12% NuPAGE Bis-Tris gel	Thermo Fisher Scientific	NP0342
InstantBlue Coomassie stain	Abcam	ab119211
Amersham Protran Western blotting membrane	Merck	GE10600012
Amersham Hybond-N+ membrane	Cytiva	RPN303B
Qubit dsDNA BR Assay Kit	Thermo Fisher Scientific	Q32853
Qubit dsDNA HS Assay Kit	Thermo Fisher Scientific	Q32854
Dynabeads MyOne Streptavidin T1	Thermo Fisher Scientific	65601
Amylose resin	New England Biolabs	E8021S
1-Methyl-7-nitroisatoic anhydride (1M7)	Merck	908401
Quick-RNA Fungal/Bacterial RNA Miniprep Kit	Zymo Research	R2014
Superscript II reverse transcriptase	Thermo Fisher Scientific	18064014
Q5 Hot Start High-Fidelity DNA Polymerase	New England Biolabs	M0493L
Nextera XT Library Prep Kit	Illumina	FC-131-1024
**Software**		
HHpred	Hildebrand et al, 2009	
Design and analysis 2	Thermo Fisher Scientific	
AlphaFold	Abramson et al, 2024	
ChimeraX-1.8	Pettersen et al, 2021	
ShapeMapper v2.1.5	Busan et al, 2018	
Image Lab	Bio-Rad	
GraphPad Prism 8	GraphPad	
**Other**		
QuantStudio 5 Real-Time PCR system	Thermo Fisher Scientific	
NextSeq 500 and 2000 platforms	Illumina	

### Strains and constructs

*S. pombe* strains used in this study are listed in Appendix Table S1. Epitope tags were introduced at genomic loci as follows: a 3×FLAG tag at the N-terminus of Pop6 (KanMX6 selection), a 3×HA tag at the N-terminus of Pop100 (NatMX6), and 3×HA tags at the C-termini of Pop7 and Pop4 (NatMX6), all under the endogenous promoters of the respective genes. PP1950 (*ter1_C926T* mutant) was generated by integrating the *ter1* fragment with endogenous promoter and C926T mutation to *ter1::ura4*^+^ by 5-Fluoroorotic acid counterselection. All gene integrations were verified by sequencing. Plasmids used in this study are listed in Appendix Table S2 and were introduced into *S. pombe* by electroporation. Plasmids pRH96 and pRH97 were generated by gene synthesis and subcloned into pJW10 (Leonardi et al, 2008), replacing the wild-type TER1 region between the MluI and NcoI sites. Plasmids pRH98, pRH115, pRH116, and pRH117 were created by site-directed mutagenesis of pJW10. Oligonucleotides used in this study are listed in Appendix Table S3.

### RNA immunoprecipitation

Native cell extracts for RNA immunoprecipitation (RNA IP) were prepared using the freezer mill method as described previously (Paez-Moscoso et al, 2022). Extracts (3 mg) were diluted to 5 mg/ml with TMG (300) buffer (10 mM Tris-HCl, pH 8.0, 1 mM MgCl₂, 10% (v/v) glycerol, 300 mM sodium acetate), supplemented with cOmplete EDTA-free protease inhibitor cocktail (Merck), 0.5 mM PMSF, 1 mM EDTA, and 0.1 mM DTT. An aliquot (50 μl) was frozen as an input control.

Protein G Dynabeads (30 μl per IP; Thermo Fisher, 10004D) were coated with 20 μg antibody per 100 μl bead suspension using anti-FLAG (Merck, F3165), anti-HA (Thermo Fisher, 26183), anti-c-Myc (Merck, M4439), or anti-V5 (Thermo Fisher, R960-25). Beads were incubated with antibody in 200 μl 1× PBS + 0.02% (v/v) Tween-20 for 30 min at room temperature, washed twice with PBS + Tween-20, and once with TMG (300). Coated beads were then incubated with 500 μl extract supplemented with 0.1% (v/v) Tween-20 and 40 U RNasin Plus RNase inhibitor (Promega, N2611) for 2 h at 4 °C with gentle rotation. Beads were washed three times with 500 μl TMG (300) plus 0.1% Tween-20, and once with 500 μl TMG (50) (identical to TMG (300) but with 50 mM sodium acetate). Beads were resuspended in 80 μl TMG (50) and frozen in liquid nitrogen for RNA extraction.

### RNA purification and RT–qPCR

Input and IP samples from the RNA immunoprecipitations were digested with stop buffer (20 mM Tris-HCl, pH 7.5, 40 mM EDTA, 5 mg/ml SDS, 2 mg/ml Proteinase K) for 15 min at 50 °C and extracted once with phenol:chloroform:isoamylalcohol (25:24:1, equilibrated with 50 mM sodium acetate, pH 5.2) and once with chloroform:isoamyl alcohol (24:1, equilibrated with 50 mM sodium acetate, pH 5.2). RNA was purified using the RNA Clean & Concentrator-25 kit (Zymo Research, R1018) according to the manufacturer’s instructions. Briefly, RNA was mixed with two volumes of RNA-binding buffer and three volumes of 100% ethanol. The mixture was transferred to a Zymo-Spin IICR column, centrifuged at 14,000×*g* for 30 s, and the flow-through was discarded. The column was washed with 400 μl RNA Wash Buffer, then treated with 5 U DNase I (Zymo Research, E1010) in 75 μl DNA Digestion Buffer for 15 min at room temperature. Columns were washed once with 400 μl RNA Prep Buffer and twice with RNA Wash Buffer (700 μl and 400 μl). RNA was eluted in 40 μl water.

For total RNA isolation, *S. pombe* cells cultured in YES medium to mid-log phase (0.5–1 × 10⁷ cells/ml) were lysed using a 6850 freezer mill and extracted with phenol/chloroform as described (Paez-Moscoso et al, 2022).

Purified RNA was reverse transcribed using the SuperScript VILO cDNA Synthesis Kit (Thermo Fisher, 11754250) in 20 μl reactions containing 2 μg total RNA (or RNA from input IPs, or ¼ of RNA from IPs), 1× VILO Reaction Mix, and 2 μl SuperScript Enzyme Mix. Reactions were incubated at 42 °C for 60 min, followed by 85 °C for 5 min. RNase H (5 U, NEB, M0297S) was added, and samples were incubated at 37 °C for 20 min and 65 °C for 20 min. cDNA was diluted 1:10 with water and used for qPCR in 10 μl reactions containing 5 μl 2× PerfeCTa SYBR Green FastMix Low ROX (Quantabio, 733-1390), 2 μl primer mix (2.5 μM each forward and reverse), 2 μl cDNA, and 1 μl water. qPCR was run in technical triplicate on a ViiA 7 or QuantStudio 5 Real-Time PCR system (Thermo Fisher).

For total RNA, expression levels were normalized to *act1* and *his1*. For IP RNA, values were normalized to the input. Each qPCR experiment included three biological replicates, and statistical analysis was performed on log₂-transformed values. Each sample was compared to the corresponding control using an unpaired *t* test assuming unequal variances (Microsoft Excel).

### Northern blot

RNA from input and IP samples was digested with stop buffer and purified via phenol/chloroform extraction followed by ethanol precipitation. The RNA was resuspended and mixed with an equal volume of 2× RNA loading dye (NEB, B0363S), heated at 75 °C for 5 min, and separated on a 4% (v/v) polyacrylamide (29:1)/8 M urea gel in 1× TBE buffer at 15 W for 80 min. RNA was transferred to a Biodyne nylon membrane (Pall Corporation) at 400 mA for 1 h in 0.5× TBE.

Crosslinking was performed using a Stratalinker (Stratagene) with 254-nm UV light at 120 mJ/cm². The TER1 probe (PCR product spanning nucleotides 536–998) was labeled using High Prime (Roche) and [α-³²P]-dCTP. The MRP1 probe (oligo Bloli6958) was labeled with T4 polynucleotide kinase (PNK) and [γ-³²P]-ATP.

Hybridizations were performed with 10 million cpm of each probe in Church-Gilbert buffer (TER1 at 60 °C, MRP1 at 42 °C). Membranes were washed with 0.1× SSC, 0.1% (w/v) SDS, exposed to Phosphorimager screens, and analyzed using a Typhoon scanner.

### Co-immunoprecipitation and western blot

To prepare native extracts for co-immunoprecipitation (co-IP), 2.5–5 ×  10⁸ mid-log phase *S. pombe* cells were resuspended in 500 μl Ext200 buffer (20 mM Tris-HCl, pH 8.0, 200 mM NaCl, 3 mM MgCl₂, 10% (v/v) glycerol, 1 mM EDTA, 0.5% Triton X-100, cOmplete EDTA-free protease inhibitor cocktail (Merck), 0.5 mM PMSF, and 0.1 mM DTT). Cells were transferred to Lysing Matrix Y tubes (MP Biomedicals) and lysed using a FastPrep-24 bead beater with four 20 s bursts at 5.5 m/s. Lysates were transferred to 1.5-ml tubes and clarified by centrifugation at 20,000×*g* for 20 min at 4 °C. Supernatants were collected, and protein concentrations were measured by Bradford assay.

For RNase A-treated samples, extracts were incubated with 2 μg/ml RNase A for 90 min at 4 °C before immunoprecipitation. IPs were performed as described for RNA IPs, but using Ext200 buffer for both the IP and wash steps. After washing, beads were resuspended in 50 μl 1× protein sample buffer (NuPAGE LDS buffer containing 50 mM DTT and 2% (w/v) SDS). Input samples were mixed with an equal volume of 2× protein sample buffer. Input and IP samples were heated at 75 °C for 10 min and resolved on 4–12% NuPAGE Bis-Tris gels (Thermo Fisher) in 1× MOPS running buffer (Thermo Fisher, NP0001).

Proteins were transferred to nitrocellulose membranes (Merck, GE10600012) using western transfer buffer (3.03 g/l Tris, 14.4 g/l glycine, 20% (v/v) methanol) at 100 V for 1 h. Membranes were blocked in 1× TTBS (20 mM Tris-HCl, pH 7.5, 137 mM NaCl, 0.1% (v/v) Tween-20) supplemented with 5% (w/v) nonfat dry milk and probed with the following primary antibodies: mouse anti-FLAG-HRP (Sigma, A8592; 1:2500), mouse anti-V5-HRP (Invitrogen, 46-0708; 1:5000), rabbit anti-c-Myc (Santa Cruz Biotechnology, sc-789; 1:2500), and rabbit anti-HA (Cell Signaling Technology, 3724S; 1:2500). Goat anti-rabbit-HRP (Thermo Fisher, 31460) was used as the secondary antibody at 1:5000.

### Genomic DNA preparation and telomere length analysis

Genomic DNA was extracted using phenol/chloroform as previously described (Pan et al, 2015). DNA concentrations were measured with the Qubit dsDNA BR Assay Kit (Thermo Fisher, Q32853). For telomere length analysis, 750 ng of genomic DNA was digested with EcoRI for 8–15 h, separated on a 1% agarose gel, and transferred to an Amersham Hybond-N+ membrane by capillary transfer. DNA was crosslinked to the membrane using a UV crosslinker with 254-nm light at 120 mJ/cm². The membrane was probed for telomeric sequences and *rad16* as a loading control, and imaging was performed as described previously (Pan et al, 2015).

### Telomerase activity assays

Telomerase activity assays from immunoprecipitations were performed as described (Paez-Moscoso et al, 2022).

### Recombinant protein purification

pET expression vectors encoding His₆-3C-tagged Pop6 or His₆-MBP-3C-tagged Pop6 and Pop7 were transformed into *E. coli* BL21-CodonPlus (DE3)-RIL cells (Agilent). Cultures (2 L) were grown in TB medium with 30 mg/L kanamycin at 37 °C to an OD₆₀₀ of 0.6, then cooled on ice to 18 °C. Protein expression was induced with 0.5 mM IPTG and continued for 21 h at 18 °C. Cells were harvested by centrifugation (4000×*g*, 15 min), resuspended in ice-cold lysis buffer (30 mM Tris-Cl, 500 mM NaCl, 10 mM imidazole, 1 mM MgCl₂, 1 mM TCEP, 5% glycerol, cOmplete EDTA-free protease inhibitor cocktail, 100 U/ml Benzonase; pH 8.0), and lysed via high-pressure homogenization at 1.9 kbar using a Constant Systems CF1 cell disruptor.

Lysates were clarified by centrifugation (40,000×*g*, 30 min, 4 °C) and applied to a HisTrap FF 5 ml Ni-NTA column (Cytiva) using a Bio-Rad NGC Quest Plus chromatography system (used for all steps). The column was washed with 15 column volumes (CV) of wash buffer (30 mM Tris-Cl, 500 mM NaCl, 10 mM imidazole, 5% glycerol), followed by 5 CV of wash buffer containing 1 M NaCl, and then another 5 CV of standard wash buffer. Proteins were eluted using a linear imidazole gradient (10–500 mM) in 30 mM Tris-Cl, 500 mM NaCl, and 5% glycerol over 10 CV.

For His₆-MBP-3C-Pop6, elution fractions were concentrated using Amicon Ultra-15 centrifugal filters (10 kDa cutoff, Millipore) and subjected to size-exclusion chromatography on a Superdex 200 16/60 pg column (Cytiva) in 25 mM Tris-Cl (pH 7.4), 150 mM NaCl, 10% glycerol.

To purify untagged Pop6 and Pop7, peak fractions of His₆-3C-Pop6 and His₆-MBP-3C-Pop7 were dialyzed overnight at 4 °C in cleavage buffer (30 mM Tris-Cl, 500 mM NaCl, 10 mM imidazole, 1 mM DTT, 5% glycerol) with His₆-3C protease (1:100 w/w) to remove affinity tags. The dialyzed material was passed over a HisTrap FF 5 ml column to remove cleaved tags and protease. Untagged proteins were concentrated using Amicon Ultra-15 filters (3 kDa cutoff, Millipore) and further purified by size-exclusion chromatography on a Superdex 200 16/60 pg column in 25 mM Tris-Cl (pH 7.4), 150 mM NaCl, 10% glycerol.

Final protein fractions (untagged Pop6, Pop7, and His₆-MBP-3C-Pop6) were concentrated to >100 μM, aliquoted, snap-frozen in liquid nitrogen, and stored at −80 °C.

### In vitro pulldown experiments

Biotinylated RNA probes were synthesized by Dharmacon with HPLC purification (sequences listed in Appendix Table S3) to assess Pop6 and Pop7 binding. Dynabeads MyOne Streptavidin T1 (40 μl per pulldown; Thermo Fisher, 65601) were washed three times with 1× B&W buffer (5 mM Tris-HCl, pH 7.5, 0.5 mM EDTA, 1 M NaCl), twice with Solution A (0.1 M NaOH, 0.05 M NaCl), and twice with Solution B (0.1 M NaCl). Beads were resuspended in 2× B&W buffer to a final concentration of 5 μg/μl (twice the original volume).

Each RNA probe (4 μg) was diluted with water to match the bead suspension volume and incubated with beads in the presence of 12.8 U RNasin Plus RNase inhibitor (Promega) for 30 min at room temperature with gentle rotation. Beads were washed three times with 1× B&W buffer and once with Ext200 buffer.

Recombinant Pop6, Pop7, or a Pop6+Pop7 mix was diluted in Ext200 buffer to 2.34 μM per protein. A 50 μl aliquot was mixed with 50 μl 2× protein sample buffer as input. The protein solution (200 μl) and 16 U RNasin Plus were added to RNA-coated or uncoated control beads. Pulldown was performed at 4 °C for 3 h with gentle rotation. Beads were washed four times for 7 min each with Ext200 buffer, resuspended in 40 μl 1× protein sample buffer, heated at 75 °C for 10 min, and separated on 12% NuPAGE Bis-Tris gels (Thermo Fisher) in 1× MES buffer. Gels were stained with InstantBlue Coomassie stain (Abcam, ab119211) and imaged using the Chemidoc MP Imaging System (Bio-Rad) with Coomassie blue gel settings. Inverted images were generated using Image Lab 6.0.1.

To test direct Pop6-Pop7 interactions, purified His₆-MBP-3C-Pop6 or His₆-MBP was incubated with untagged Pop7 (1 μM each) in 100 μl Ext200 buffer. Amylose resin (20 μl per pulldown; NEB, E8021) was used to capture MBP-tagged proteins. Binding was performed for 3 h at 4 °C with gentle rotation. Beads were washed three times for 7 min with Ext200 buffer, resuspended in 40 μl 1× protein sample buffer, and analyzed as described above for the RNA pulldown experiments.

### Fluorescence polarization assays

To measure the fluorescence polarization of Pop6, Pop7, or the complex binding to TER1 P3-like domain, 6-FAM-labeled WT or C926U RNA probes were synthesized by Integrated DNA Technologies (IDT) with HPLC purification (sequences listed in Appendix Table S3). In a 386-well plate (Corning, Low-Volume, Polysyrene, black), 5 nM of each RNA was incubated with varying concentrations of recombinant Pop6, Pop7, or a 1:1 mixture of both in a total volume of 15 μl FP buffer (20 mM Tris-Cl pH 7.5, 200 mM NaCl, 1 mM DTT, 0.1 mM EDTA, 5% glycerol, 0.05% Triton X-100). After 10 min incubation at 23 °C, FP was analyzed on a Spark 20 M plate reader (Tecan) at 23 °C (excitation wavelength: 485 nm; emission wavelength 520 nm; gain: 120; flashes: 15, integration time: 40 μs). Normalized FP values were calculated by subtracting the FP value of the RNA-only well. The mean normalized FP values including standard deviation from three independent experiments were plotted using GraphPad Prism 8. Dissociation constants (Kd) and Hill slopes were determined by fitting a nonlinear regression (“specific binding with Hill slope”).

### SHAPE-MaP

In-cell SHAPE-MaP was performed as described in (Smola et al, 2015; Smola and Weeks, 2018). Briefly, 4 × 10^8^ mid-log phase *S. pombe* cells were resuspended in 9 ml YES medium and split into two tubes. One tube received 500 μl of 100 mM 1M7 (Merck, 908401) ( + ) and the other 500 μl DMSO (−) as a control. After mixing, cells were incubated at 32 °C for 5 min with shaking and collected. RNA was extracted using the Quick-RNA Fungal/Bacterial RNA Miniprep Kit (Zymo Research, R2014).

Cell pellets were resuspended in 800 μl RNA Lysis Buffer, transferred to ZR BashingBead Lysis Tubes, and lysed in a FastPrep-24 bead beater (three 20 s bursts at 5.5 m/s). Lysates were centrifuged at 14,000×*g* for 1 min, and the supernatants were transferred to Zymo-Spin IIICG columns for binding and cleanup. Flow-through was mixed with an equal volume of 100% ethanol, loaded onto Zymo-Spin IICR columns, and purified using the RNA Clean & Concentrator protocol.

Gene-specific primers for MaP RT and PCR are listed in Appendix Table S3. Due to the length of TER1 ( ~ 1.2 kb), two RT primers (Bloli7828 for the 5′ half and Bloli8852 for the 3′ half) were used. For MRP1, Bloli8786 was used. RNA (1 μg in 10 μl) was mixed with 1 μl of 2 μM RT primers, denatured at 65 °C for 5 min, and cooled on ice. MaP buffer (8 μl) was added, and samples were incubated at 42 °C for 2 min. Superscript II reverse transcriptase (1 μl; ThermoFisher, 18064014) was added, and reactions were incubated at 42 °C for 3 h, followed by inactivation at 70 °C for 15 min.

cDNA was purified using G-25 microspin columns (Cytiva, 27532501). PCR was carried out in 50 μl reactions with 1× Q5 Reaction Buffer, 200 μM dNTPs, 0.5 μM of each primer, 1 U Q5 Hot Start High-Fidelity DNA Polymerase (NEB, M0493L), and 5 μl cDNA. For the TER1 5′ half, primers Bloli1339 and Bloli7828 were used with cycling: 98 °C for 30 s, followed by 25 cycles of 98 °C for 10 s, 60 °C for 30 s, 72 °C for 30 s, and a final extension at 72 °C for 2 min. For the TER1 3′ half, primers Bloli8858 and Bloli8852 were used with 26 cycles and 72 °C extension increased to 45 s. MRP1 was amplified with Bloli8785 and Bloli8786 using the TER1 5′ protocol.

An aliquot (5 μl) of each PCR product was checked on an agarose gel. The remaining product was purified with the MinElute PCR Purification Kit (Qiagen, 28006), and concentrations were measured using the Qubit dsDNA HS Assay Kit. Amplicons were pooled at equimolar ratios, and dual-indexed libraries were prepared using the Nextera XT Library Prep Kit (Illumina, FC-131-1024). Libraries were sequenced as 2×150 bp paired-end reads on a NextSeq 500 or 2000 platform.

SHAPE data were analyzed using ShapeMapper v2.1.5 (https://github.com/Weeks-UNC/shapemapper2). Default parameters were used, except for: --max-primer-offset 600, --window-to-trim 1, and --min-length-to-trim 20. Normalized SHAPE reactivity profiles were generated per biological replicate. The mean ± SEM from three replicates per sample was plotted using Python 3.9.13. For the overlapping region of TER1 (nt 528–543), data from both amplicons (3 replicates each) were combined to yield six total replicates.

### Protein structure visualization and overlay

AlphaFold-predicted structures of Pop6 (AF-G2TRL5-F1-v4) and Pop7 (AF-O94251-F1-v4) were downloaded from AlphaFold Protein Structure Database (Varadi et al, 2024). The Pop6-Pop7 interaction was predicted by Alphafold server (https://alphafoldserver.com) (Abramson et al, 2024) using Pop6 and Pop7 protein sequences. Visualization and overlay analysis of the structures were carried out using ChimeraX-1.8 (Pettersen et al, 2021).

## Supplementary information

Appendix

Peer Review File

Source data Fig. 1

Source data Fig. 2

Source data Fig. 3

Source data Fig. 4

Source data Fig. 5

Source data Fig. 6

Source data Fig. 7

Source Data Appendix S2B

Expanded View Figures

## Data Availability

The SHAPE-MaP sequencing data is available at https://www.ncbi.nlm.nih.gov/bioproject/ under the accession number PRJNA1250409. Scripts used in this study are available at 10.5281/zenodo.15601336. The source data of this paper are collected in the following database record: biostudies:S-SCDT-10_1038-S44319-026-00782-9.
