# Supplementary material for: RNase P/MRP subunits chaperone telomerase holoenzyme assembly in fission yeast
Source: EMBO Rep. 2026 Apr 28;27(12):3277–302. doi: 10.1038/s44319-026-00782-9 (PMC13303942; doi:10.1038/s44319-026-00782-9)
Supplement: Supplementary file 1 — Appendix [file 44319_2026_782_MOESM1_ESM.pdf]

Appendix

Table of Content

Appendix Figure S1. Structural similarities between Pop6 orthologs in *S. pombe*, humans, and *S. cerevisiae*. -- 2

Appendix Figure S2. Random spore analysis of *pop6⁺/pop6Δ* diploid strain. ------- 3

Appendix Figure S3. Expression and purification of recombinant Pop6 and Pop7. -- 4

Appendix Figure S4. Electrostatic surface models of AlphaFold-predicted Pop7 and Pop6. -- 5

Appendix Figure S5. Predicted secondary structures of TER1 mature and precursor forms by Mfold. --- 6

Appendix Figure S6. RNA enrichment by immunoprecipitation of Lsm8-cMyc. ------ 7

Appendix Figure S7. SHAPE reactivity changes in TER1 structure model. ------------ 8

Appendix Figure S8. In-cell SHAPE-MaP profiles of MRP1 RNA. ----------------------- 9

Appendix Figure S9. Alternative RNA structures in the core region of TER1 predicted by Mfold. --- 10

Appendix Figure S10. deltaSHAPE of MRP1 RNA. --------------------------------------- 11

Appendix Figure S11. In-cell SHAPE-MaP profiles of the TER1 three-way junction (TWJ) domain. --- 12

Appendix Table S1. Strains used in this study. -- 13

Appendix Table S2: Plasmids used in this study. -- 14

Appendix Table S3: Oligos used in this study. --- 15

A

```
   ss_pred   ceeccccchHHHHHHHHHHHhcc--eeeEEEEEeccceeEeeeHHHHHHHHHHhcCCChhheeee
Sp     2     IIEKSINAKQLSLKLWDSFVK--EKQVSFVAEGDATVKLVSLVELLKRRCNEQKIPFNQTIEL   62 (95)
             .|..+++.+.++...-..|.+ ...|.+.|-|.|--|+|+++|.+||+...  .+|.+.+
Hs     39    RVKEGSKIRNLMAFATASMAQPATRAIVFSGCGRATTKTVTCAEILKRRLAG----LHQVTRL   97 (199)
   ss_pred   EECCCCcHHHHHHHHHHHhcCCCCcEEEEEeehHHHHHHHHHHHHHHHHcCC----CeEEEEe
```

B

```
   ss_pred   CceeccccchHHHHHHHHHHHhcc-------eeeEEEEEeccceeEeeeHHHHHHHHHHhcCCChhheeee
Sp     1     MIIEKSINAKQLSLKLWDSFVK-------EKQVSFVAEGDATVKLVSLVELLKRRCNEQKIPFNQTIEL   62 (95)
             |.|.+.++.+..+...-..|.. .+.|-++|.|.|.=|+||++|++||+-.+.+..++|.+.+.-....
Sc     47    HGISKNDNIKKSVNKLDKQINMADRSLGLQQVVCIFSYGPHIQKMLSILEIFKKGYIKNNKKIYQWNKL   97 (158)
   ss_pred   EEEcCCChHHHHHHHHHHHHhhccccCcCcEEEEEEecHHHHHHHHHHHHHHHHchhcCCcEEEEeee


   ss_pred   ccccchh-hccccccCccccCCcceEEEE
Sp     63    IPSEEQ-SSDTLIHGNRRSLPQLKIDLQ   89 (95)
              ....      +....++..|.|.|-|-
Sc     98    TSFDIKREGRNELQEERLKVPILVTLVS   143 (158)
   ss_pred   eEEEEeecCccccccceEEeccEEEEEEE
```

C **D** **E** **F**

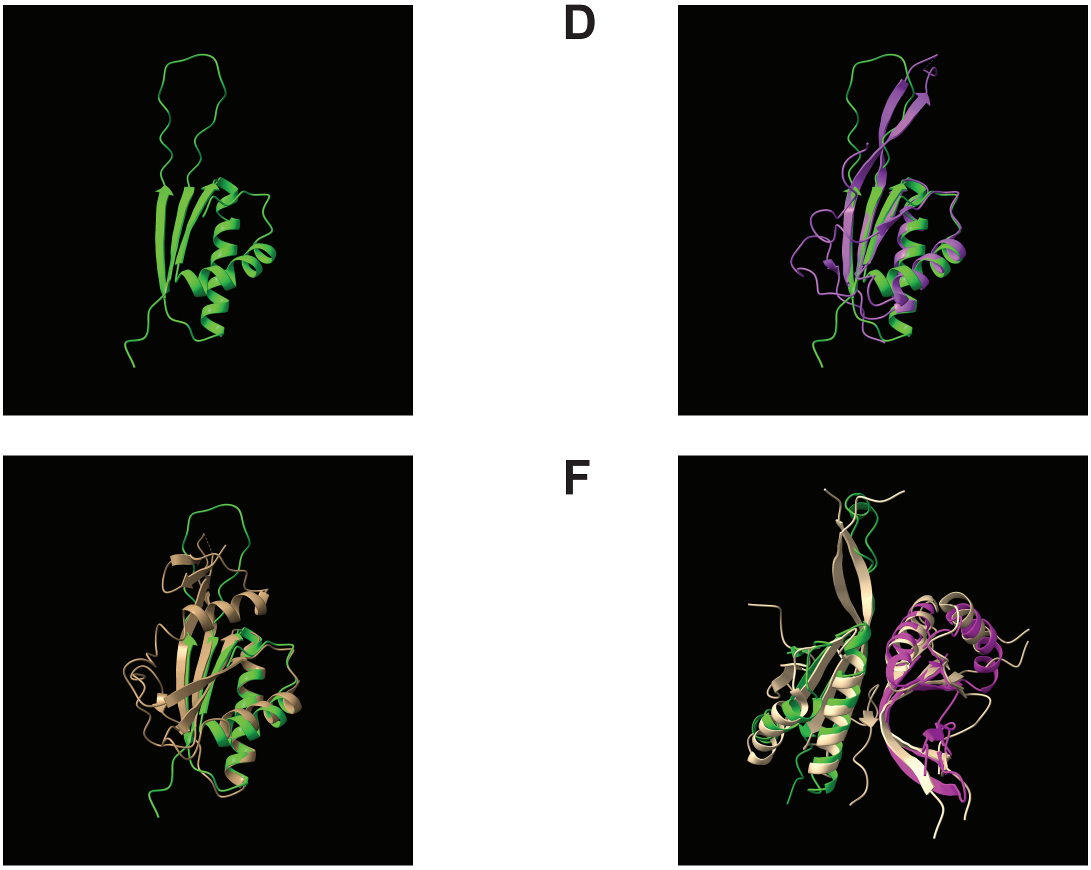

Appendix Figure S1. Structural similarities between Pop6 orthologs in *S. pombe*, humans, and *S. cerevisiae*.

(A) HHpred-based alignment and secondary structure prediction of *S. pombe* Pop6 (Sp) and human RPP25 (Hs). Symbols between sequences indicate alignment quality: "|" very good, "+" good, "." neutral, "−" poor, and "=" very poor. "ss_pred" denotes predicted secondary structures: H (helix), E (sheet), and C (coil). Numbers in the parentheses indicate full lengths of the proteins. **(B)** HHpred alignment and secondary structure prediction of *S. pombe* Pop6 (Sp) and *S. cerevisiae* Pop6 (Sc), using the same notation as in (A). **(C)** Predicted 3D structure of *S. pombe* Pop6 (model: AF-G2TRL5-F1-v4) generated by AlphaFold. **(D)** Structural superposition of AlphaFold-predicted *S. pombe* Pop6 (green) with the crystal structure of human RPP25 (PDB ID: 6CWX, purple). **(E)** Structural superposition of AlphaFold-predicted *S. pombe* Pop6 (green) with the crystal structure of S. cerevisiae Pop6 (PDB ID: 3IAB, brown). **(F)** AlphaFold-Multimer model of the Pop6 (green)–Pop7 (magenta) interaction overlaid with the crystal structure of the human RPP20–RPP25 complex (PDB ID: 6CWX, beige).

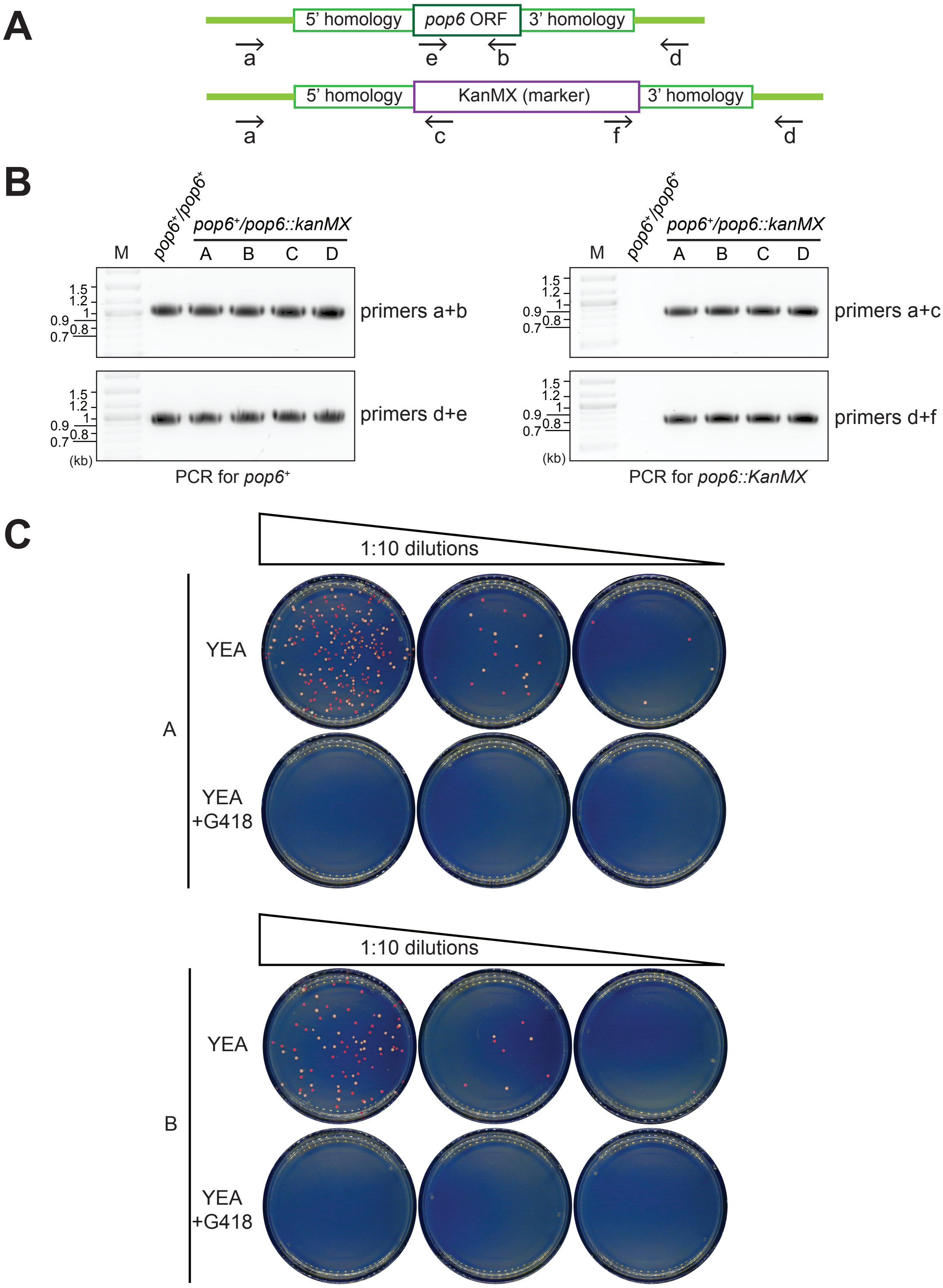

Appendix Figure S2. Random spore analysis of *pop6*⁺/pop6Δ diploid strain.
(A) Schematic of the primers used to verify the strain. One allele of the *pop6*⁺ is replaced by the KanMX marker. **(B)** Diagnostic PCR to verify the diploid *pop6⁺/pop6::kanMX* strain using genomic DNA from 4 isolates and the parental WT *pop6⁺/pop6*+ strain. Primers a+b and d+e (left panels) give products of expected size from the *pop6*⁺ allele, and primers a+c and d+f (right panels) give products of expected size from the *pop6::kanMX* allele. The parental *pop6⁺/pop6⁺* strain gives only the products from *pop6*⁺ allele. Isolates A and B are used for further analysis. **(C)** Spores from two independent isolates of the diploid *pop6⁺/pop6::kanMX* strain was plated with 1:10 serial dilutions to YEA without selection and to YEA+G418 that selects for *pop6*Δ by the *KanMX* cassette. The total number of colonies from all three dilutions were counted to be 230 for YEA and 0 for YEA+G418 for A isolate, and 93 for YEA and 0 for YEA+G418 for B isolate.

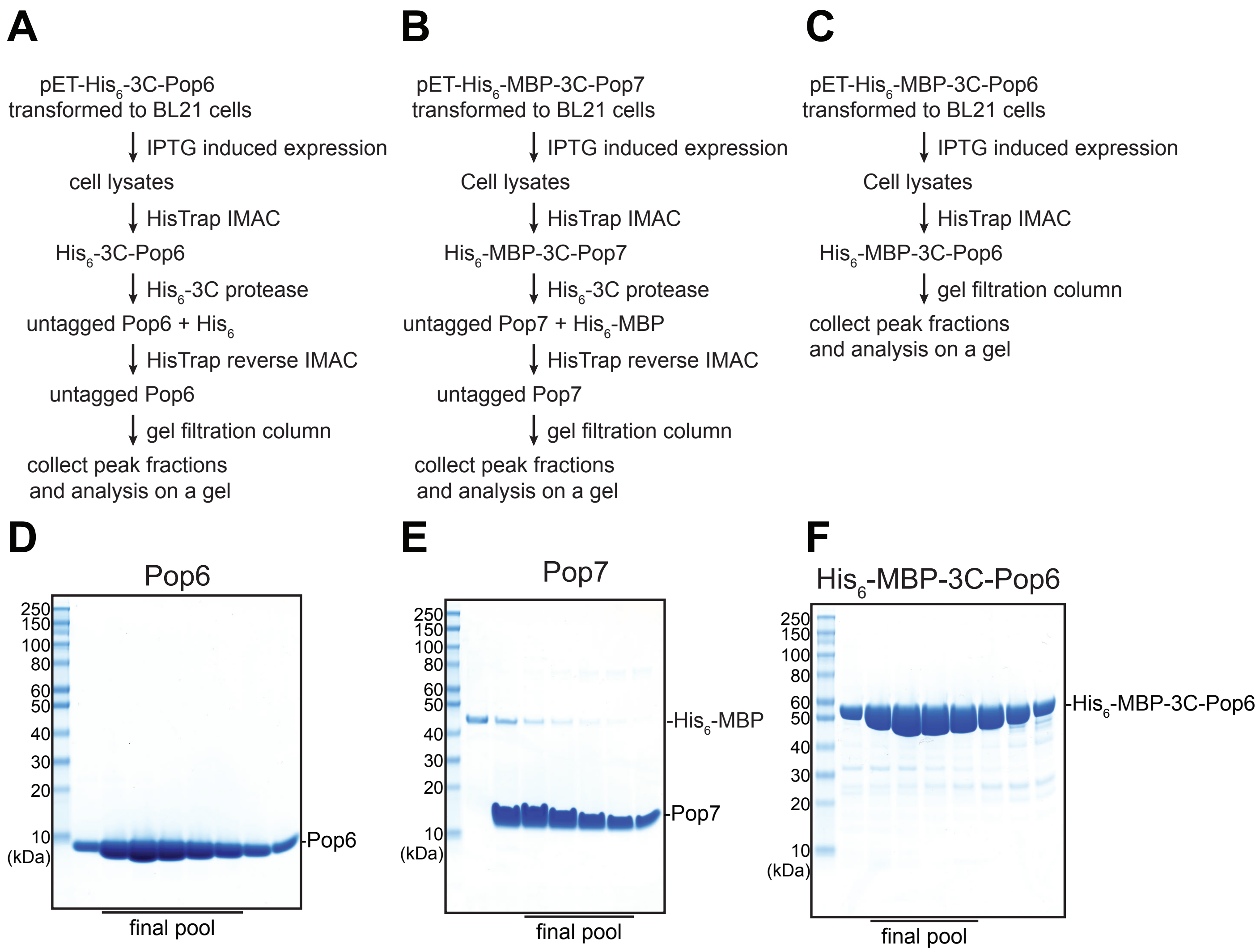

Appendix Figure S3. Expression and purification of recombinant Pop6 and Pop7. **(A–C)** Flowcharts summarizing the purification workflows for recombinant Pop6, Pop7 and His$_6$-MBP-3C-Pop6 expressed in E. coli. **(D)** Fractions from gel filtration of untagged Pop6 (corresponding to panel A), visualized by Coomassie staining. Labeled fractions were pooled for downstream experiments. **(E)** Fractions from gel filtration of untagged Pop7 (corresponding to panel B), analyzed as in (D). **(F)** Fractions from gel filtration of His$_6$-MBP-3C-Pop6 (corresponding to panel C), analyzed as in (D).

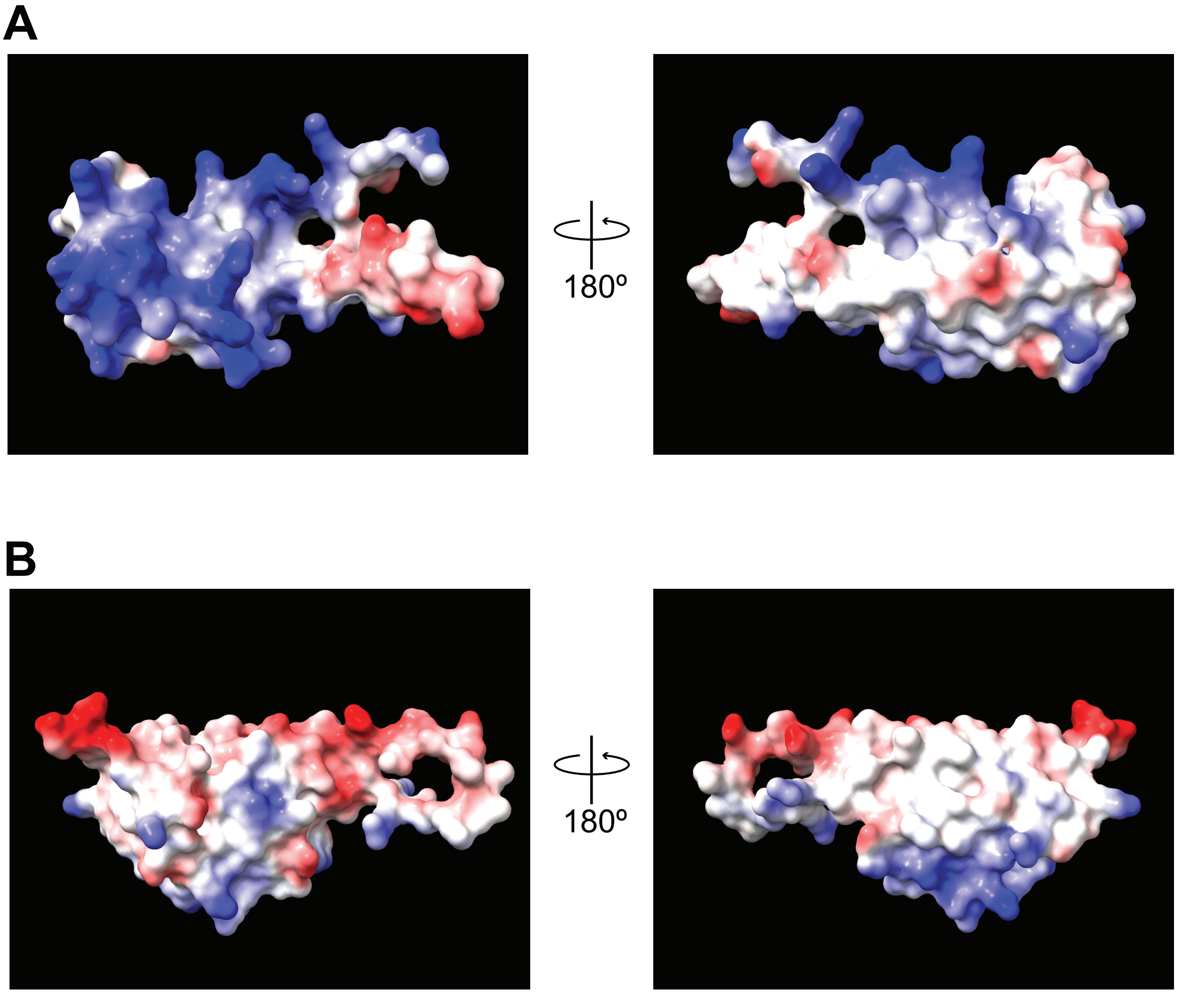

Appendix Figure S4. Electrostatic surface models of AlphaFold-predicted Pop7 and Pop6.
(A) Electrostatic surface of AlphaFold-predicted Pop7. Red regions indicate negative charge, blue regions indicate positive charge, and white indicates neutral charge. **(B)** Electrostatic surface of AlphaFold-predicted Pop6.

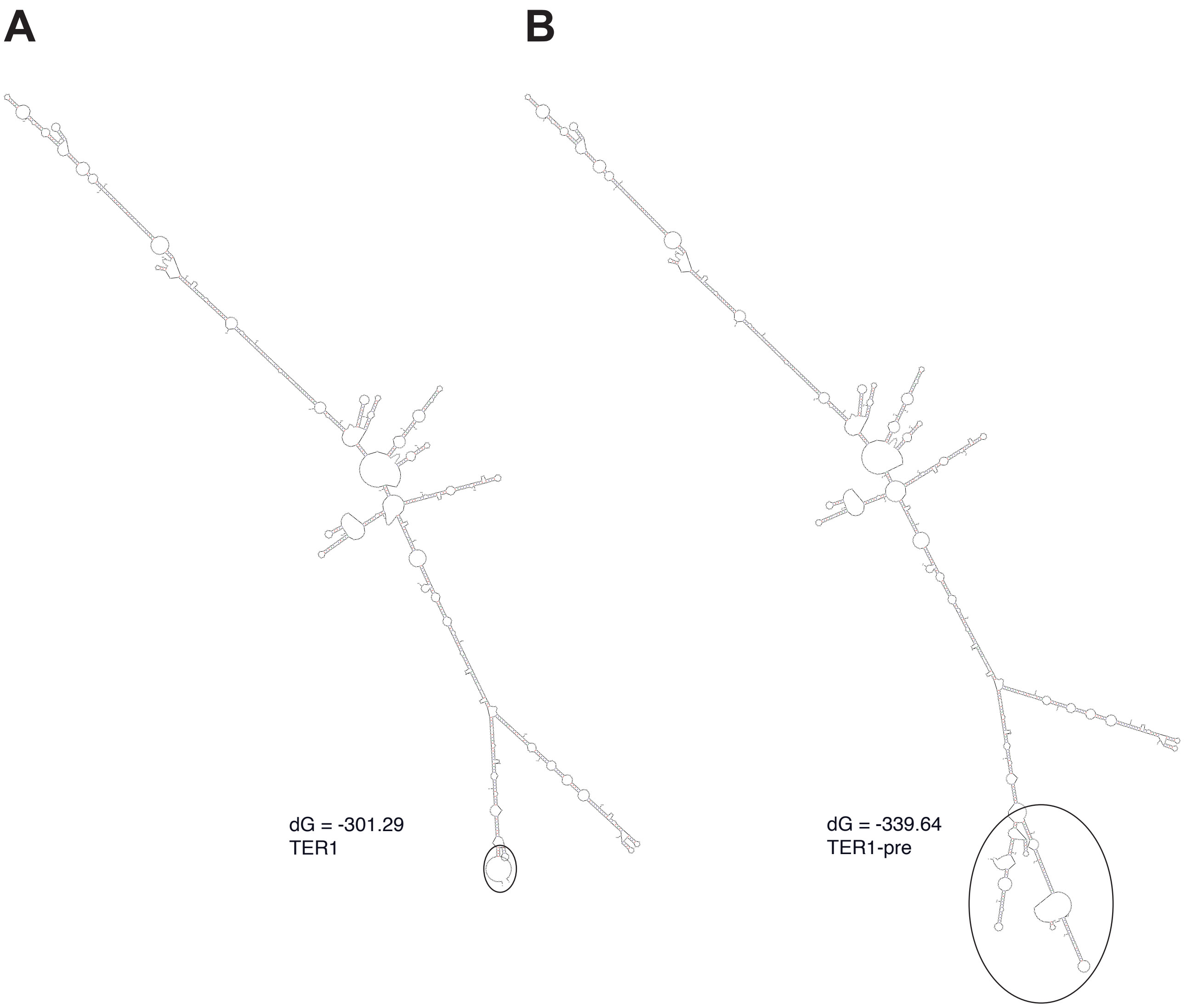

Appendix Figure S5. Predicted secondary structures of TER1 mature and precursor forms by Mfold.
(A) Mfold-predicted secondary structure of mature TER1 (nt 1-1213) with the lowest free energy (ΔG). The ellipse marks the 5’ 18 nt (nt 1-18) and 3’ 26 nt (nt 1188-1213) which folds differently in the precursor form. **(B)** Mfold-predicted secondary structure of TER1 precursor (nt 1-1419) with the lowest free energy. The ellipse marks the regions that are different from the mature structure in (A) (nt 1-18 and nt 1188-1419).

Lsm8-cMyc IP

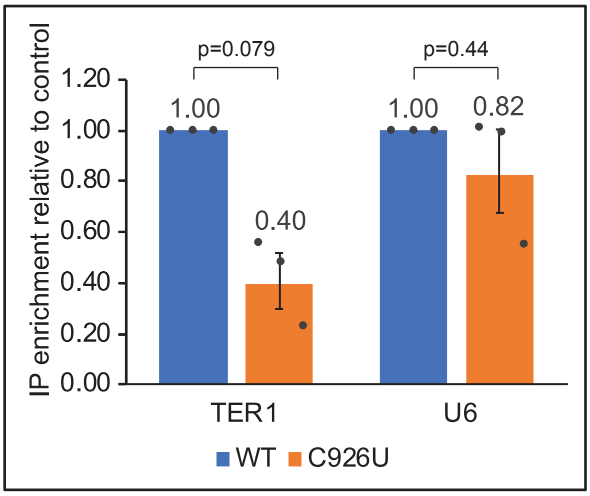

Appendix Figure S6. RNA enrichment by immunoprecipitation of Lsm8-cMyc. Immunoprecipitation (IP) was performed from extracts containing Lsm8-cMyc and either wild-type (WT) or C926U mutant TER1 expressed from a plasmid. Bars represent mean RNA levels (±SEM, n = 3) recovered in the mutant relative to WT, normalized to input levels and quantified by RT-qPCR. Statistical analysis: unpaired t-tests (n=3).

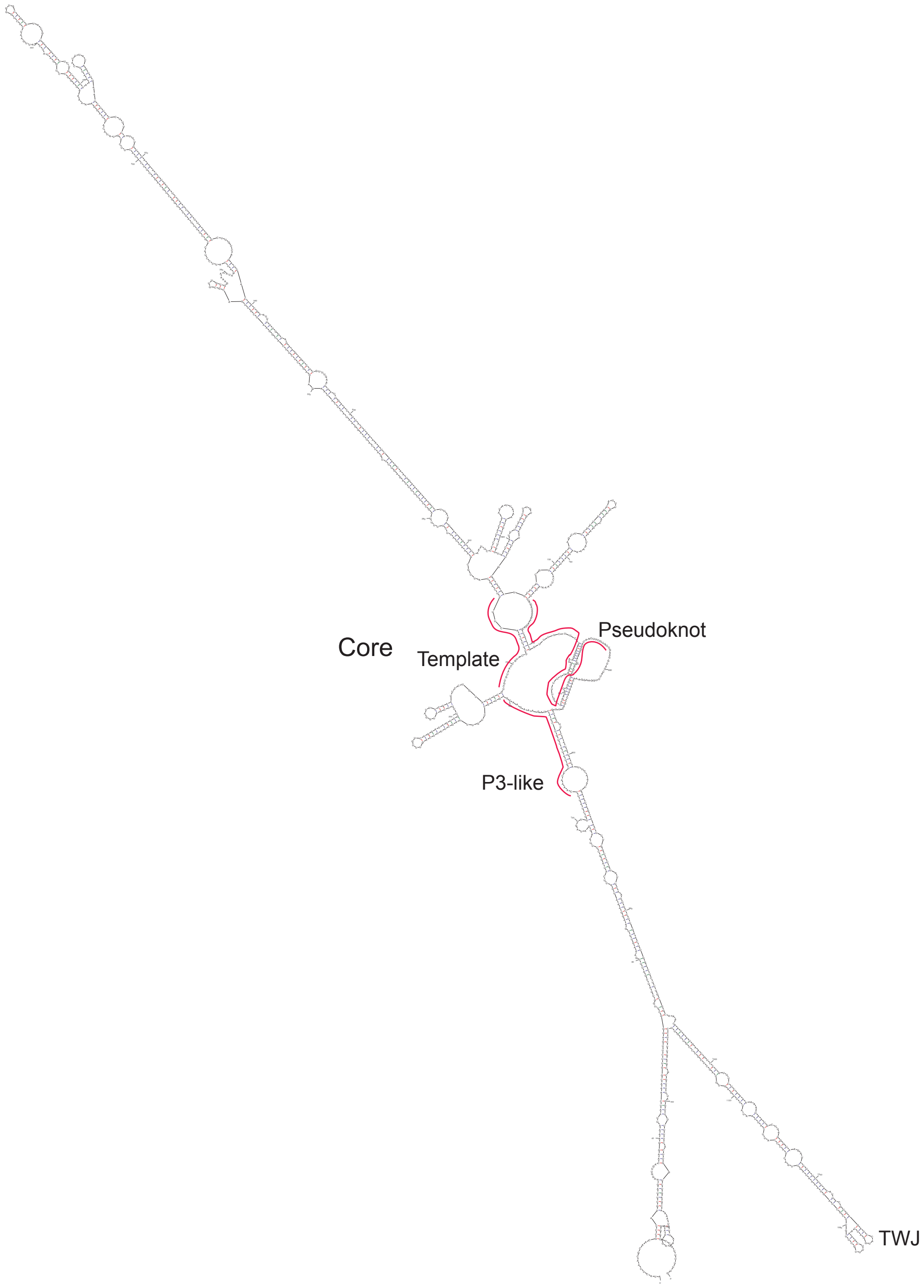

Appendix Figure S7. SHAPE reactivity changes in TER1 structure model.
TER1 structure model with the red lines marking the regions that change the most in the PCA loading analysis (correspond to the red underlined regions in Fig. EV4B).

A

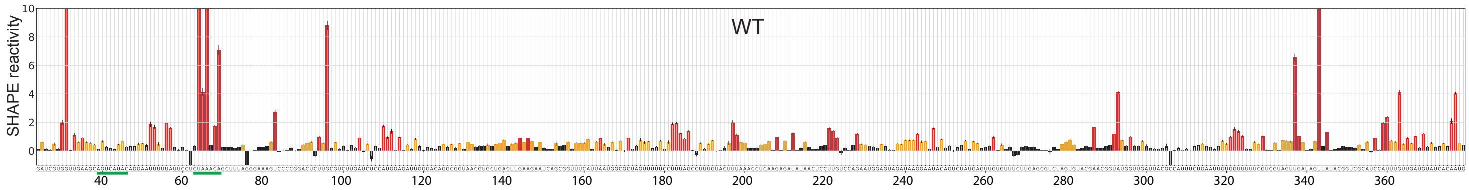

B

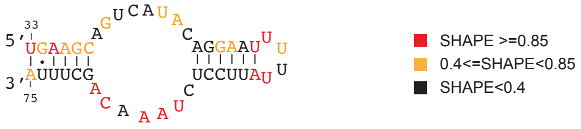

SHAPE of MRP1 P3 domain in WT cells

Appendix Figure S8. In-cell SHAPE-MaP profiles of MRP1 RNA.
(A) In-cell SHAPE-MaP of MRP1 RNA in WT background. Bars represent mean SHAPE reactivities (±SEM, n = 3), with a maximum display value of 10. Horizontal lines below the trace indicate the P3 loop region. **(B)** SHAPE reactivities of nucleotides in the MRP1 P3 region mapped onto the predicted RNA structure, colored according to WT reactivity values.

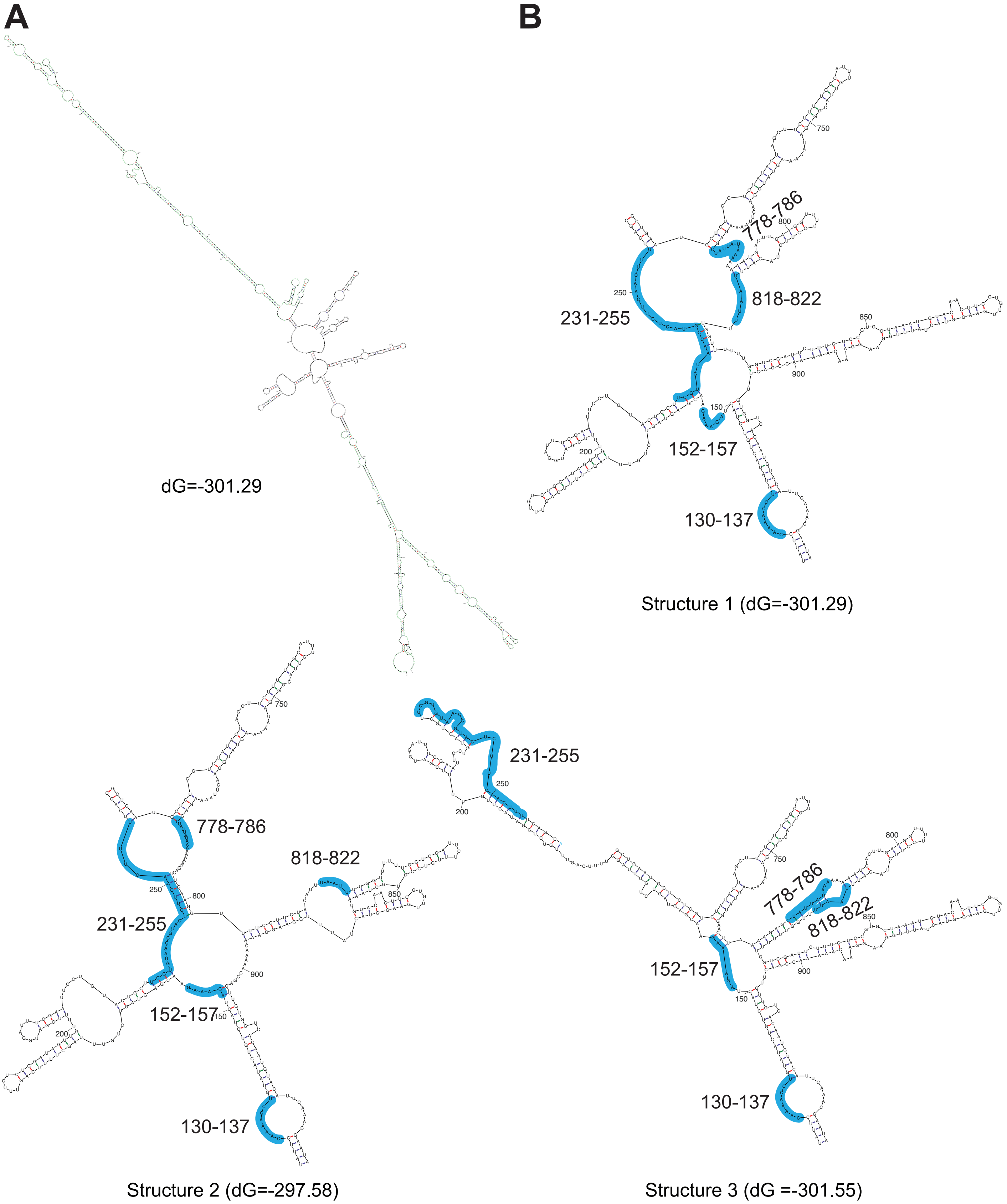

Appendix Figure S9. Alternative RNA structures in the core region of TER1 predicted by Mfold.
(A) Mfold-predicted secondary structure of full-length TER1 with the lowest free energy (ΔG). The core region, where most SHAPE reactivity differences are observed and multiple structural alternatives were predicted, is shown in black. The remainder of the RNA is shown in green. **(B)** Three alternative structures of the core region, extracted from the top three Mfold-predicted structures. Blue lines highlight regions with SHAPE reactivity differences between ter1_C926U and WT.

A

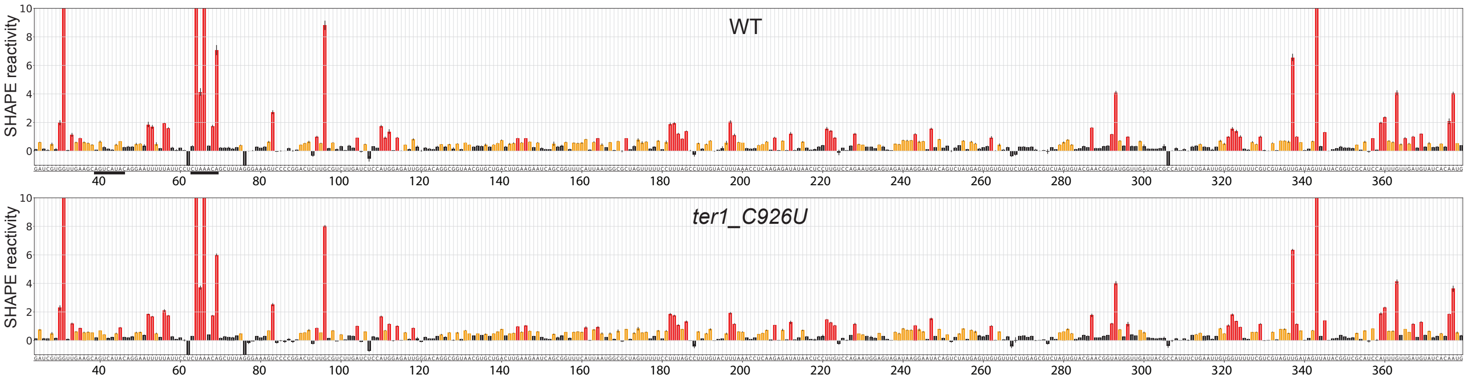

B

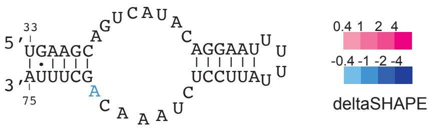

ter1_C926U minus WT deltaSHAPE of MRP1 P3 domain

Appendix Figure S10. deltaSHAPE of MRP1 RNA.
(A) In-cell SHAPE-MaP of MRP1 RNA in WT (top, the same data as Appendix Figure S8) and *ter1_C926U* (bottom) strains. Horizontal lines below the WT trace indicate the P3 loop region. **(B)** deltaSHAPE comparison of *ter1_C926U* minus WT in the MRP1 P3 region. Nucleotides with deltaSHAPE < –0.4 and $p < 0.05$ are colored blue; deltaSHAPE > 0.4 and $p < 0.05$ are colored pink. Nucleotides with deltaSHAPE between –0.4 and 0.4 or $p \geq 0.05$ are shown in black.

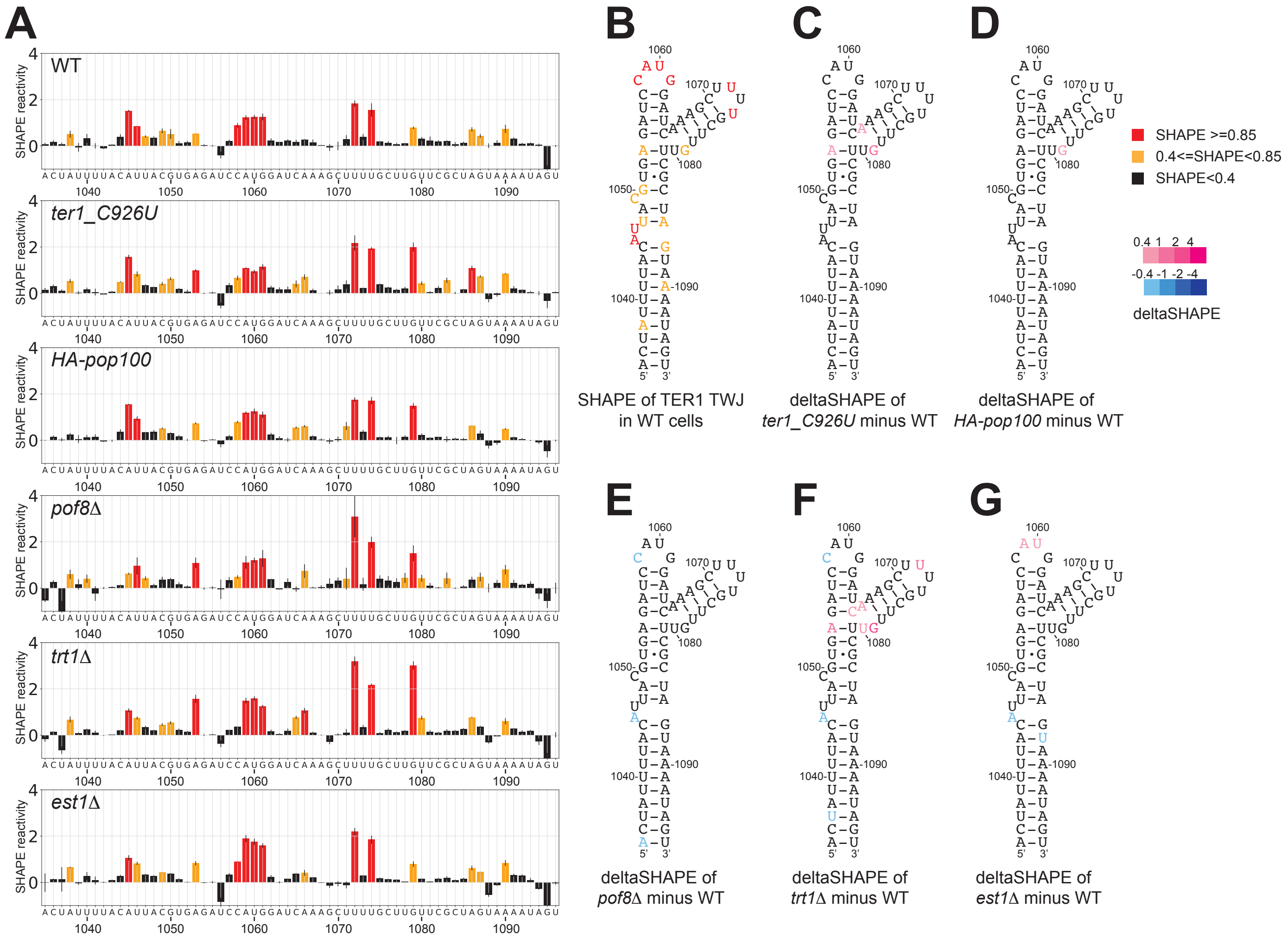

Appendix Figure S11. In-cell SHAPE-MaP profiles of the TER1 three-way junction (TWJ) domain.

(A) SHAPE reactivity profiles of the TER1 TWJ region (nucleotide positions 1035–1096) in WT, *ter1_C926U*, *HA-pop100*, *pof8*Δ, *trt1*Δ, and *est1*Δ strains. **(B)** Nucleotides in the TWJ region colored according to SHAPE reactivities in WT cells, mapped onto the predicted structure. **(C–G)** deltaSHAPE profiles for the TWJ region comparing each mutant (*ter1_C926U*, *HA-pop100*, *pof8*Δ, *trt1*Δ, or *est1*Δ) to WT (mutant minus WT). Significance was assessed by unpaired t-test ($n = 3$). Nucleotides with deltaSHAPE < –0.4 and $p < 0.05$ are shown in blue; deltaSHAPE > 0.4 and $p < 0.05$ are shown in pink; Nucleotides with deltaSHAPE between –0.4 and 0.4 or $p \geq 0.05$ are shown in black.

Appendix Table S1. Strains used in this study

Strain name	genotype	Source
PP60sp	*$h^{?}$ ade6-M? his3-D1 leu1-32 ura4-D18 trt1::$ura4^{+}$*	This study
PP138	*h^{-} ade6-M216 his3-D1 leu1-32 ura4-D18*	Lab stock
PP237sp	*$h^{?}$ ade6-M? his3-D1 leu1-32 ura4-D18 est1::kanMX6*	This study
PP1732	*h^{-} ade6-M216 his3-D1 leu1-32 ura4-D18 pop7::pop7-3xHA-natMX6*	This study
PP1733	*h^{-} ade6-M216 his3-D1 leu1-32 ura4-D18 pop100::natMX6-3xHA-pop100*	This study
PP1743	*h^{+} ade6-M210 his3-D1 leu1-32 ura4-D18 pop7::pop7-3xHA-natMX6*	This study
PP1746	*h^{+} ade6-M210 his3-D1 leu1-32 ura4-D18 pop100::natMX6-3xHA-pop100*	This study
PP1762	*$h^{?}$ ade6-M216 his3-D1 leu1-32 ura4-D18 pop7::pop7-3xHA-natMX6 lsm4::lsm4-13myc-hphMX6*	This study
PP1765	*$h^{?}$ ade6-M216 his3-D1 leu1-32 ura4-D18 pop100::natMX6-3xHA-pop100 lsm4::lsm4-13myc-hphMX6*	This study
PP1778	*h^{-} ade6-M216 his3-D1 leu1-32 ura4-D18 pop6::kanMX6-3xFLAG-pop6*	This study
PP1783	*$h^{?}$ ade6-M216 his3-D1 leu1-32 ura4-D18 pop6::kanMX6-3xFLAG-pop6 pop7::pop7-3xHA-natMX6*	This study
PP1785	*h^{+} ade6-M210 his3-D1 leu1-32 ura4-D18 pop6::kanMX6-3xFLAG-pop6*	This study
PP1791	*h^{-} ade6-M216 his3-D1 leu1-32 ura4-D18 pop4::pop4-3xHA-natMX6*	This study
PP1825	*$h^{?}$ ade6-M216 his3-D1 leu1-32 ura4-D18 pop6::kanMX6-3xFLAG-pop6 lsm4::lsm4-13myc-hphMX6*	This study
PP1851	*$h^{?}$ ade6-M210 his3-D1 leu1-32 ura4-D18 pop6::kanMX6-3xFLAG-pop6 pop100::natMX6-3xHA-pop100*	This study
PP1858	*$h^{?}$ ade6-M210 his3-D1 leu1-32 ura4-D18 pof8::kanMX6*	Paez-Moscoso *et al*, 2022
PP1950	*h^{+} ade6-M210 his3-D1 leu1-32 ura4-D18 ter1::ter1_C926U*	This study
PP2115	*$h^{?}$ ade6-M216 his3-D1 leu1-32 ura4-D18 pop7::pop7-3xHA-natMX6 pof8::kanMX6*	This study
PP2116	*$h^{?}$ ade6-M216 his3-D1 leu1-32 ura4-D18 pop100::natMX6-3xHA-pop100 pof8::kanMX6*	This study
PP2117	*$h^{?}$ ade6-M216 his3-D1 leu1-32 ura4-D18 pop6::kanMX6-3xFLAG-pop6 pof8::kanMX6*	This study
PP2133	*h^{+} ade6-M216 his3-D1 leu1-32 ura4-D18 pop7::pop7-3xHA-natMX6 trt1::$ura4^{+}$*	This study
PP2134	*h^{+} ade6-M216 his3-D1 leu1-32 ura4-D18 pop100::natMX6-3xHA-pop100 trt1::$ura4^{+}$*	This study
PP2135	*h^{+} ade6-M216 his3-D1 leu1-32 ura4-D18 pop6::kanMX6-3xFLAG-pop6 trt1::$ura4^{+}$*	This study
PP2136	*$h^{?}$ ade6-M210 his3-D1 leu1-32 ura4-D18 pop7::pop7-3xHA-natMX6 est1::kanMX6*	This study
PP2137	*$h^{?}$ ade6-M216 his3-D1 leu1-32 ura4-D18 pop100::natMX6-3xHA-pop100 est1::kanMX6*	This study
PP2138	*$h^{?}$ ade6-M216 his3-D1 leu1-32 ura4-D18 pop6::kanMX6-3xFLAG-pop6 est1::kanMX6*	This study
FP1455	*$h^{?}$ ade6-M? his3-D1 leu1-32 ura4-D18 ter1::kanMX6 [pJW10]*	This study
FP1458	*$h^{?}$ ade6-M? his3-D1 leu1-32 ura4-D18 ter1::kanMX6 [pRH96]*	This study
FP1459	*$h^{?}$ ade6-M? his3-D1 leu1-32 ura4-D18 ter1::kanMX6 [pRH97]*	This study

FP1460	*$h^?$ ade6-M? his3-D1 leu1-32 ura4-D18 ter1::kanMX6 [pRH98]*	This study
FP1470	*$h^?$ ade6-M216 his3-D1 leu1-32 ura4-D18 ter1::kanMX6 [pRH115]*	This study
FP1471	*$h^?$ ade6-M216 his3-D1 leu1-32 ura4-D18 ter1::kanMX6 [pRH116]*	This study
FP1472	*$h^?$ ade6-M216 his3-D1 leu1-32 ura4-D18 ter1::kanMX6 [pRH117]*	This study
FP1473	*$h^?$ ade6-M216 his3-D1 leu1-32 ura4-D18 ter1::kanMX6 [pDBlet]*	This study
FP1474	*$h^?$ ade6-M216 his3-D1 leu1-32 ura4-D18 ter1::kanMX6 [pJW10]*	This study
FP1480	*$h^?$ ade6-M210 his3-D1 leu1-32 ura4-D18 lsm4::lsm4-13myc-natMX6 ter1::kanMX6 [pJW10]*	This study
FP1481	*$h^?$ ade6-M210 his3-D1 leu1-32 ura4-D18 smb1::sbm1-13myc-natMX6 ter1::kanMX6 [pJW10]*	This study
FP1482	*$h^?$ ade6-M216 his3-D1 leu1-32 ura4-D18 lsm4::lsm4-13myc-natMX6 ter1::kanMX6 [pRH96]*	This study
FP1483	*$h^?$ ade6-M210 his3-D1 leu1-32 ura4-D18 smb1::sbm1-13myc-natMX6 ter1::kanMX6 [pRH96]*	This study
FP1488	*$h^?$ ade6-M? his3-D1 leu1-32 ura4-D18 pop7::pop7-3xHA-natMX6 ter1::kanMX6 [pJW10]*	This study
FP1489	*$h^?$ ade6-M? his3-D1 leu1-32 ura4-D18 pop7::pop7-3xHA-natMX6 ter1::kanMX6 [pRH96]*	This study
FP1491	*$h^?$ ade6-M? his3-D1 leu1-32 ura4-D18 est1::est1-13myc-hphMX6 ter1::kanMX6 [pJW10]*	This study
FP1492	*$h^?$ ade6-M? his3-D1 leu1-32 ura4-D18 est1::est1-13myc-hphMX6 ter1::kanMX6 [pRH96]*	This study
FP1525	*$h^?$ ade6-M216 his3-D1 leu1-32 ura4-D18 trt1::trt1-v5-hphMX6 ter1::kanMX6 [pJW10]*	This study
FP1530	*$h^?$ ade6-M216 his3-D1 leu1-32 ura4-D18 trt1::trt1-v5-hphMX6 ter1::kanMX6 [pRH96]*	This study
FP1537	*$h^?$ ade6-M? his3-D1 leu1-32 ura4-D18 lsm8::lsm8-13myc-natMX6 ter1::kanMX6 [pJW10]*	This study
FP1538	*$h^?$ ade6-M? his3-D1 leu1-32 ura4-D18 lsm8::lsm8-13myc-natMX6 ter1::kanMX6 [pRH96]*	This study
FP1572	*$h^?$ ade6-M216 his3-D1 leu1-32 ura4-D18 pof8::3xFLAG-pof8-kanMX6 ter1::natMX6 [pJW10]*	This study
FP1573	*$h^?$ ade6-M216 his3-D1 leu1-32 ura4-D18 pof8::3xFLAG-pof8-kanMX6 ter1::natMX6 [pRH96]*	This study
FP1584	*$h^?$ ade6-M216 his3-D1 leu1-32 ura4-D18 pop100::natMX6-3xHA-pop100 ter1::kanMX6 [pJW10]*	This study
FP1585	*$h^?$ ade6-M216 his3-D1 leu1-32 ura4-D18 pop100::natMX6-3xHA-pop100 ter1::kanMX6 [pRH96]*	This study
FP1596	*$h^?$ ade6-M216 his3-D1 leu1-32 ura4-D18 pop6::kanMX6-3xFLAG-pop6 ter1::kanMX6 [pJW10]*	This study
FP1597	*$h^?$ ade6-M216 his3-D1 leu1-32 ura4-D18 pop6::kanMX6-3xFLAG-pop6 ter1::kanMX6 [pRH96]*	This study

Appendix Table S2: Plasmids used in this study

Plasmid name	Description	Usage
pDBlet (1)	Empty vector	Transform to *S.pombe*
pJW10 (2)	pDblet-WT_TER1	Transform to *S.pombe*
pRH96	pDblet-ter1_C926U	Transform to *S.pombe*
pRH97	pDblet-ter1_C926A	Transform to *S.pombe*
pRH98	pDblet-ter1_924UUC->GGU	Transform to *S.pombe*

pRH115	pDblet-ter1_Δ130-137+Δ923-930	Transform to *S.pombe*
pRH116	pDblet-ter1_Δ130-137	Transform to *S.pombe*
pRH117	pDblet-ter1_Δ923-930	Transform to *S.pombe*
pET-His6-3C-Pop6	pET-His6-3C-Pop6	Transform to *E.coli* BL21(DE3) for protein purification
pET-His6-MBP-3C-Pop7	pET-His6-MBP-3C-Pop7	Transform to *E.coli* BL21(DE3) for protein purification
pET-His6-MBP-3C-Pop6	pET-His6-MBP-3C-Pop6	Transform to *E.coli* BL21(DE3) for protein purification

Appendix Table S3: Oligos used in this study

Oligo Name	oligo sequence	Usage
Bloli6958	5'-CGTTCGTACACTAGACGCTCAAGAAACAC-3'	MRP1 probe for northern blot
Bloli7827	5'-CAGTGTACGTGAGTCTTCTGCCTT-3'	RT-qPCR for TER1(3)
Bloli7828	5'-CAAAAATTCGTTGTGATCTGACAAGC-3'	RT-qPCR for TER1(3)
Bloli7825	5'-AATTGCGTATTTAGTAAGAACGCG-3'	RT-qPCR for TER1-pre (4)
Bloli7826	5'-GATTCATCACTTTCTCAAAATTTTGAAACCG-3'	RT-qPCR for TER1-pre (4)
Bloli7829	5'-GGATTCCTACGTTGGTGATGA-3'	RT-qPCR for Act1 (4)
Bloli7830	5'-CGTTGTAGAAAGTGTGATGCC-3'	RT-qPCR for Act1 (4)
Bloli7818	5'-CGAAGACGTGCTTCAGCGA-3'	RT-qPCR for His1 (5)
Bloli7819	5'-TGTCCACCTCGGAATCACTG-3'	RT-qPCR for His1 (5)
Bloli7870	5’-GAGTTGTGTTTCTTGAGCG-3’	RT-qPCR for MRP1
Bloli7871	5’-ACTAAGCCCCATTGTGATAC-3’	RT-qPCR for MRP1
Bloli7886	5’-GTAAGGTCGGAGAAACATCT-3’	RT-qPCR for RRK1
Bloli7887	5’-GACAAACGAGGCCATACT-3’	RT-qPCR for RRK1
Bloli7831	5’-ACCTGGCATGAGTTTCTGC-3’	RT-qPCR for U1 (6)
Bloli7832	5’-GACCTTAGCCAGTCCACAGTTA-3’	RT-qPCR for U1 (6)
Bloli7833	5’-GATCTTCGGATCACTTTGGTC-3’	RT-qPCR for U6 (4)
Bloli7834	5’-GGTTTTCTCTCAATGTCGCAG-3’	RT-qPCR for U6 (4)
Bloli7828	5’-CAAAAATTCGTTGTGATCTGACAAGC-3’	SHAPE-MaP RT primer and PCR Reverse primer for TER1 5’ half
Bloli1339	5’-ATACTCAACGCAACGCCCATG-3’	SHAPE-MaP PCR Forward primer for TER1 5’ half
Bloli8852	5’-TGGGCCCGTTCCTTTTAAC-3’	SHAPE-MaP RT primer and PCR Reverse primer for TER1 3’ half
Bloli8858	5’-CCTACCTTTCATTTGTATAAAATC-3’	SHAPE-MaP PCR Forward primer for TER1 3’ half
Bloli8786	5’-TTGAGCACGAGACTAAGCC-3’	SHAPE-MaP RT primer and PCR Reverse primer for MRP1
Bloli8785	5’-AAATGATACCTTTGAGCTCGAA-3’	SHAPE-MaP PCR Forward primer for MRP1
Bloli7654 (RNA)	5'Bi/CGACUUGUGGUCACAAUGUACAUUCAAACGAAUAGCAACUUGUUGCUAUUCCAAAACCUGUAUACUGUCUACUAGAAAG-3’	Wt P3 probe for in vitro pulldown
Roli1019 (RNA)	5'Bi/CGACUUGUGGUCACAAUGUACAUUUAAACGAAUAGCAACUUGUUGCUAUUCCAAAACCUGUAUACUGUCUACUAGAAAG-3’	Mt P3 probe for in vitro pulldown
Roli1024 (RNA)	5'Bi/CCAGAUAGUUGACUUAACUACAAAAGGACCUAGACAAUUCAUGGCUAAAACCGCUAAUGUGCUUAUCUCUAAUGCUGUU-3’	Scrambled control (Sc) probe for in vitro pulldown
6FAM-WT_P3 (RNA)	5’6FAM/AAUGUACAUUCAAACGAAUAGCAACUUGUUGCUAUUCCAAAACCUGUAUA-3’	WT probe for fluorescence polarization assays

6FAM-Mut_P3 (RNA)	5’6FAM/AAUGUACAUUUAAACGAAUAGCAACUUGU UGCUAUUCCAAAACCUGUAUA-3’	Mut probe for fluorescence polarization assays

References

1. Brun, C., Dubey, D.D. and Huberman, J.A. (1995) pDblet, a stable autonomously replicating shuttle vector for Schizosaccharomyces pombe. *Gene*, **164**, 173-177.
2. Leonardi, J., Box, J.A., Bunch, J.T. and Baumann, P. (2008) TER1, the RNA subunit of fission yeast telomerase. *Nat Struct Mol Biol*, **15**, 26-33.
3. Mennie, A.K., Moser, B.A. and Nakamura, T.M. (2018) LARP7-like protein Pof8 regulates telomerase assembly and poly(A)+TERRA expression in fission yeast. *Nat Commun*, **9**, 586.
4. Collopy, L.C., Ware, T.L., Goncalves, T., S, I.K., Yang, Q., Amelina, H., Pinder, C., Alenazi, A., Moiseeva, V., Pearson, S.R. *et al.* (2018) LARP7 family proteins have conserved function in telomerase assembly. *Nat Commun*, **9**, 557.
5. Fujita, I., Tanaka, M. and Kanoh, J. (2012) Identification of the functional domains of the telomere protein Rap1 in Schizosaccharomyces pombe. *PLoS One*, **7**, e49151.
6. Tang, W., Kannan, R., Blanchette, M. and Baumann, P. (2012) Telomerase RNA biogenesis involves sequential binding by Sm and Lsm complexes. *Nature*, **484**, 260-264.
