# Supplementary figures and images for: RNase P/MRP subunits chaperone telomerase holoenzyme assembly in fission yeast
Source: EMBO Rep. 2026 Apr 28;27(12):3277–302. doi: 10.1038/s44319-026-00782-9 (PMC13303942; doi:10.1038/s44319-026-00782-9)

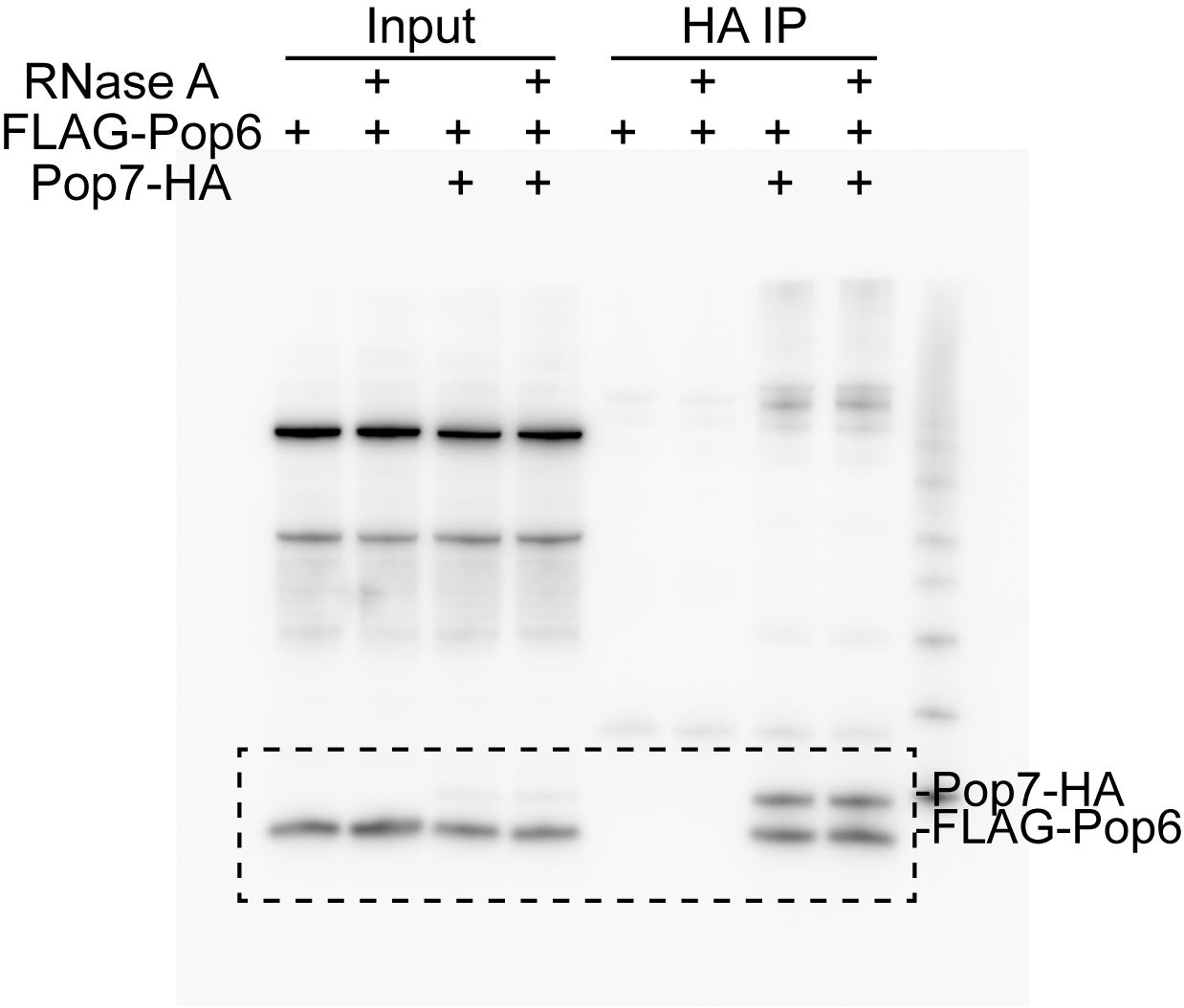

Supplement: Supplementary file 3 — Source data Fig. 1 [file 44319_2026_782_MOESM3_ESM.zip › Figure 1/1A/western_HA reprobe.tif]

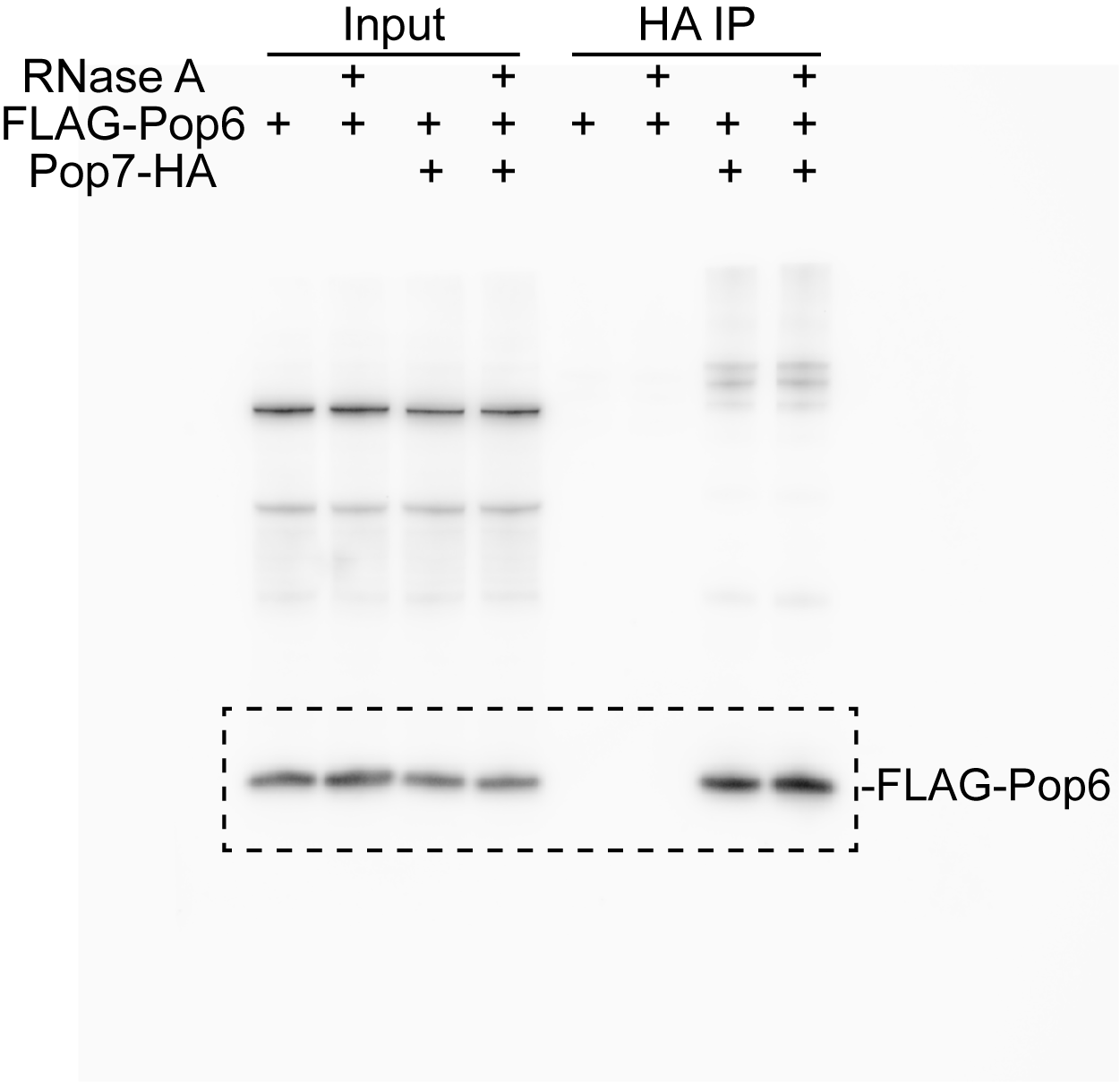

Supplement: Supplementary file 3 — Source data Fig. 1 [file 44319_2026_782_MOESM3_ESM.zip › Figure 1/1A/western_FLAG.tif]

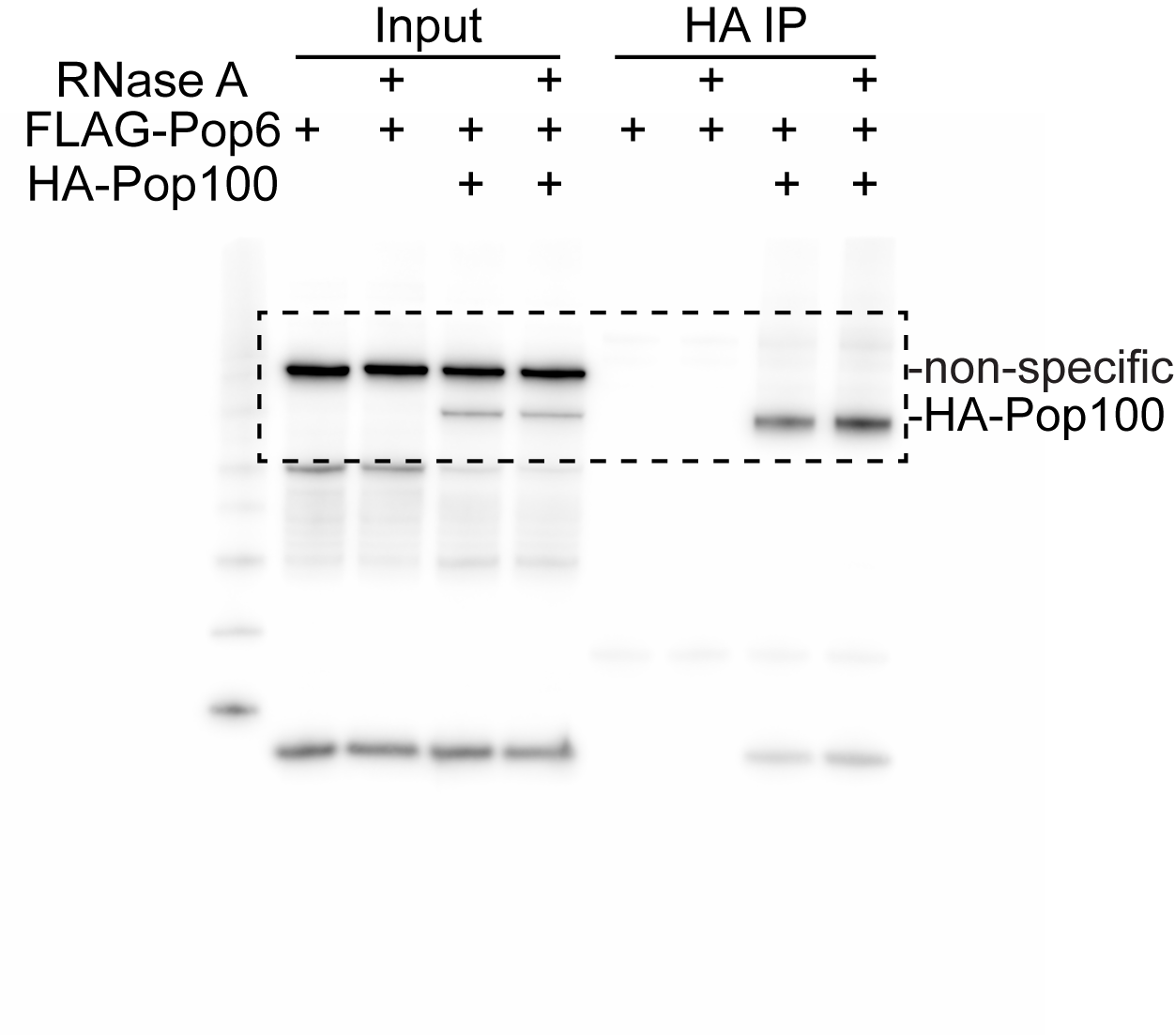

Supplement: Supplementary file 3 — Source data Fig. 1 [file 44319_2026_782_MOESM3_ESM.zip › Figure 1/1B/western_HA reprobe.tif]

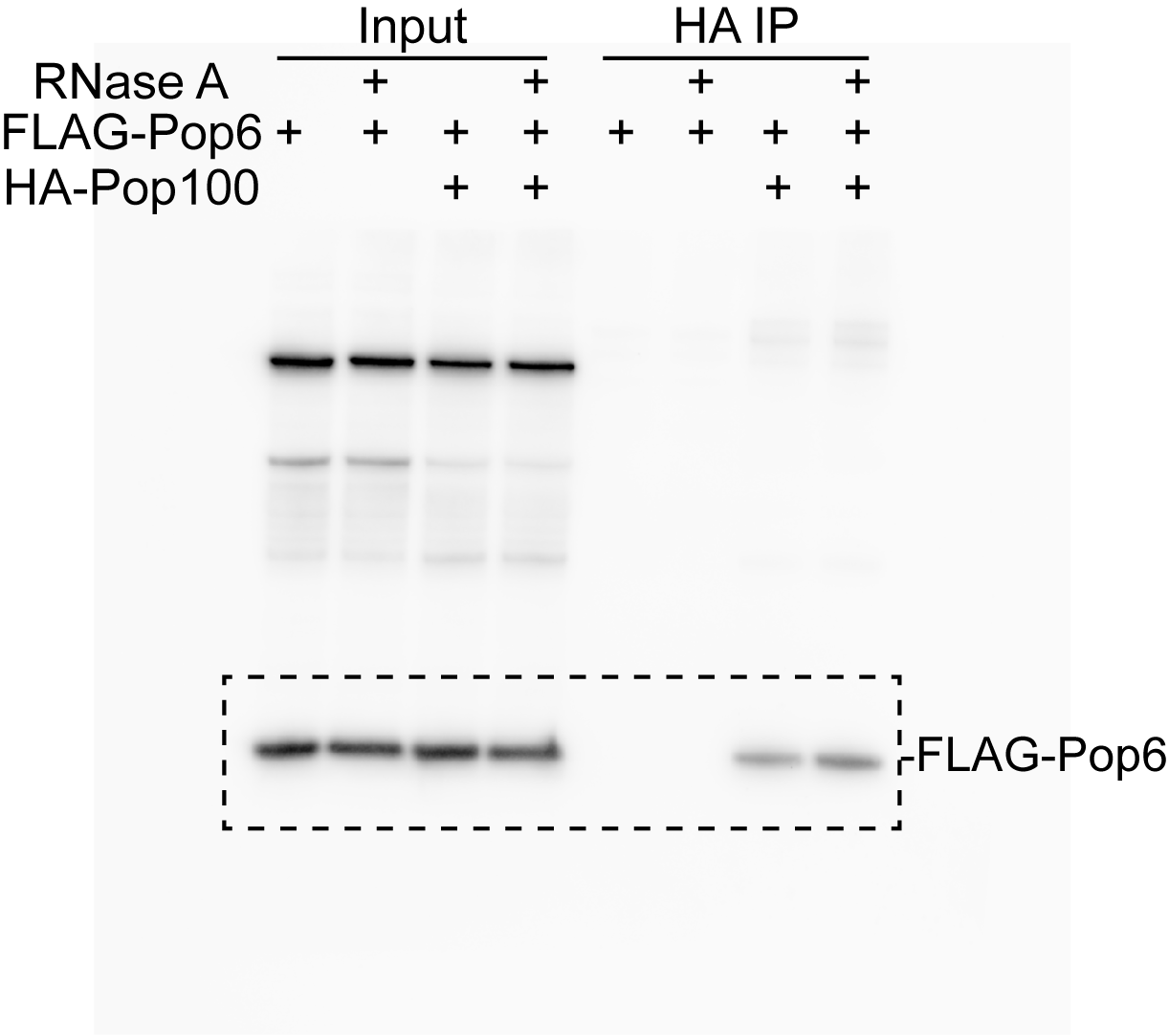

Supplement: Supplementary file 3 — Source data Fig. 1 [file 44319_2026_782_MOESM3_ESM.zip › Figure 1/1B/western_FLAG.tif]

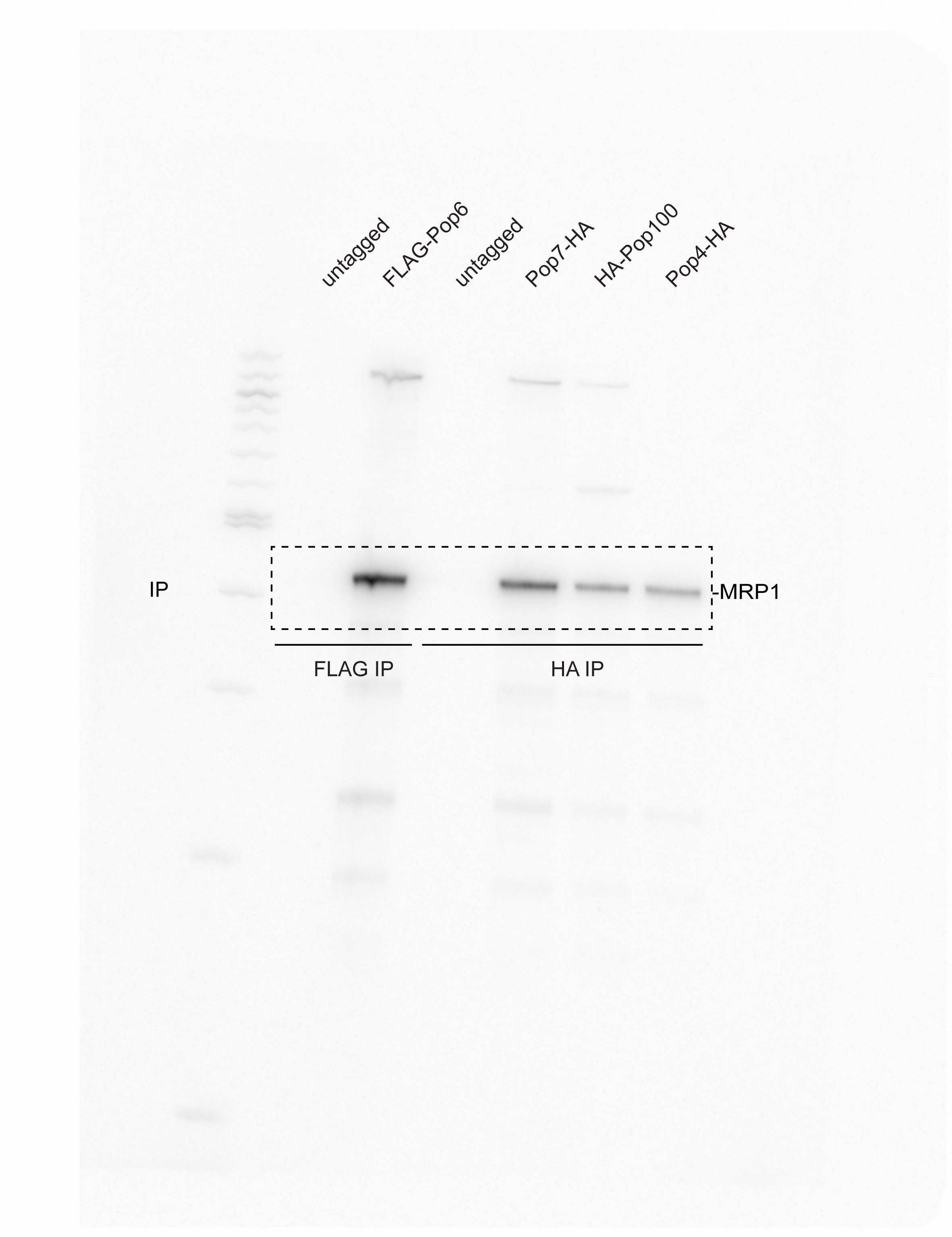

Supplement: Supplementary file 3 — Source data Fig. 1 [file 44319_2026_782_MOESM3_ESM.zip › Figure 1/1D/IP northern_MRP1 reprobe.tif]

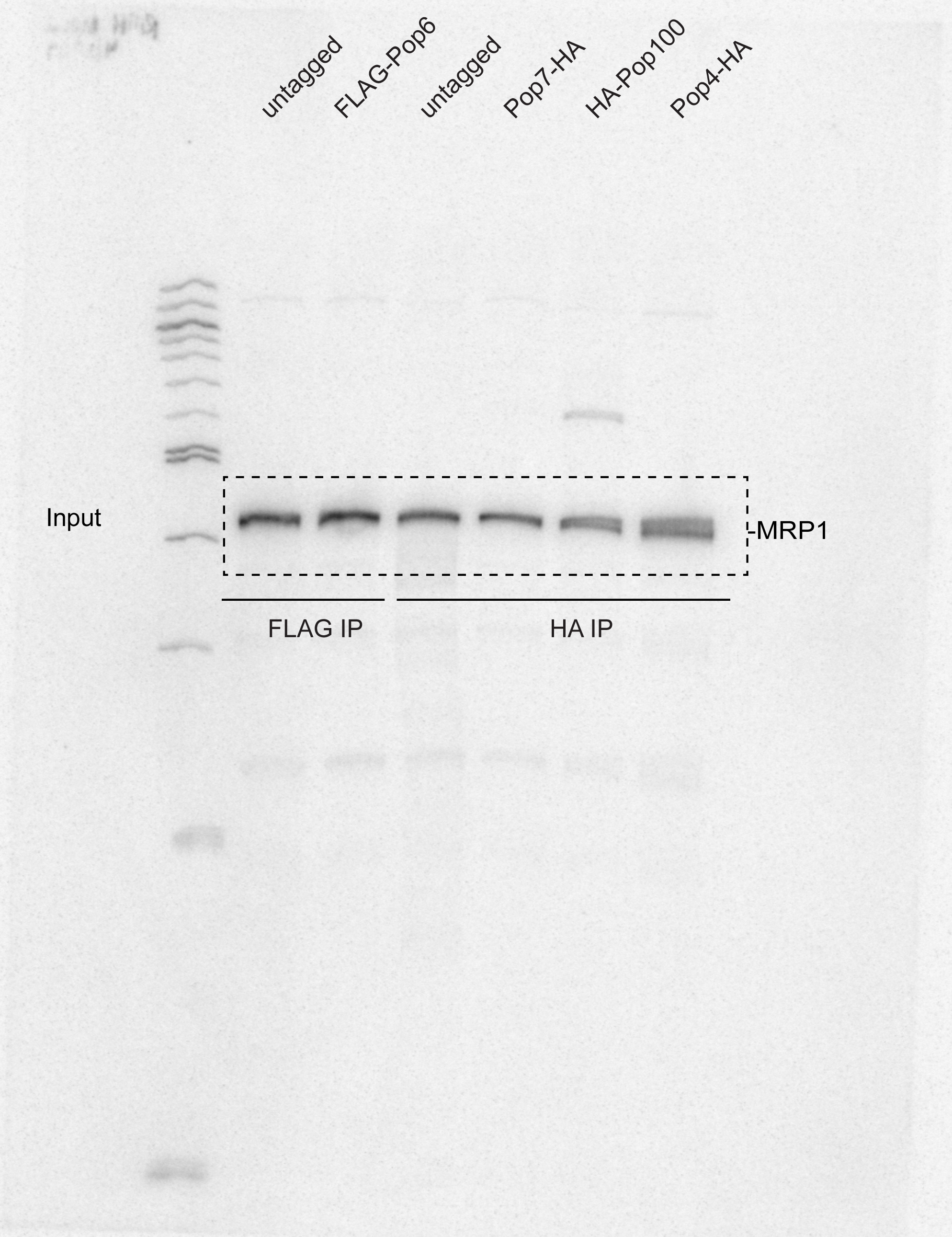

Supplement: Supplementary file 3 — Source data Fig. 1 [file 44319_2026_782_MOESM3_ESM.zip › Figure 1/1D/input northern_MRP1 reprobe.tif]

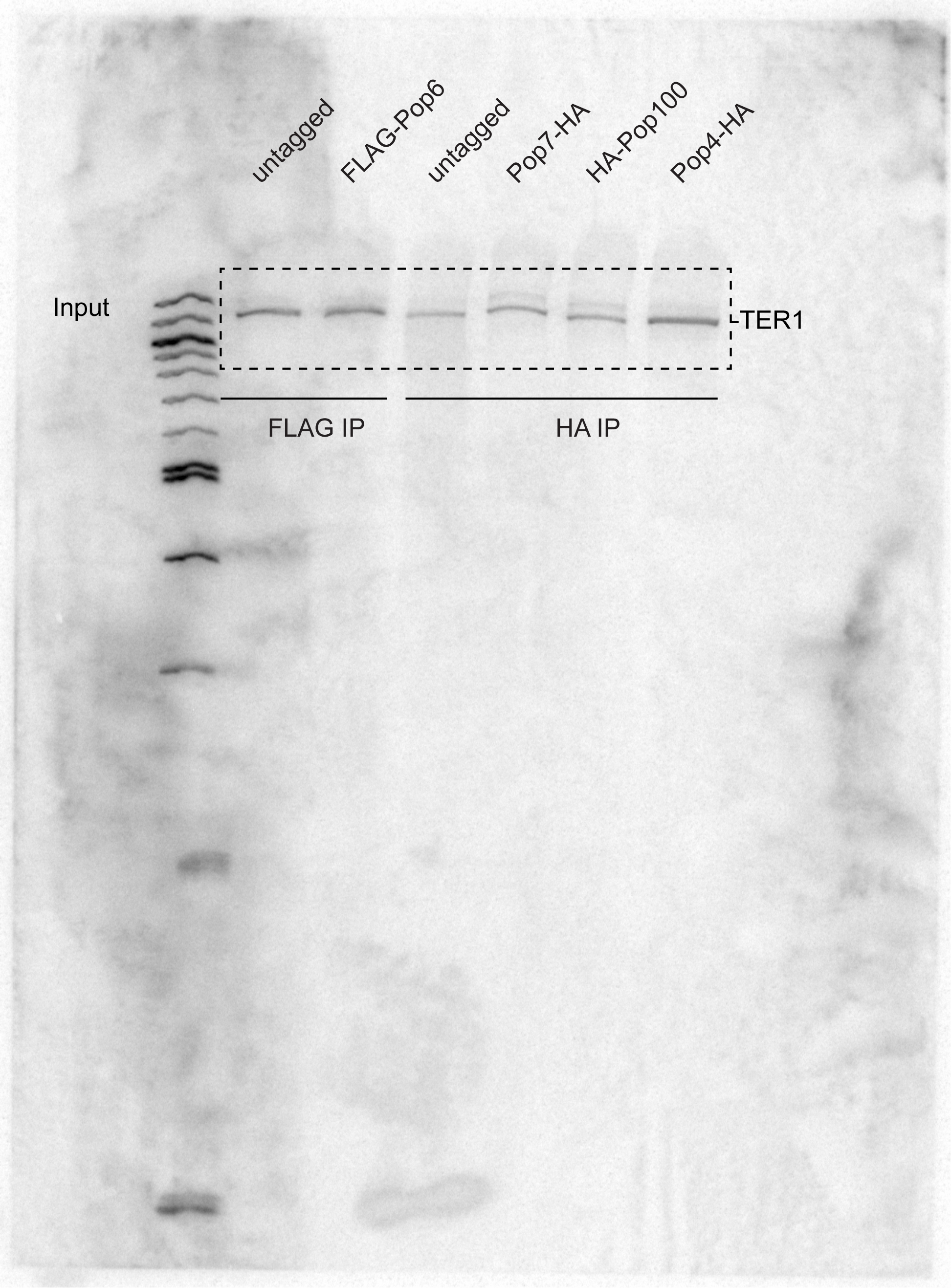

Supplement: Supplementary file 3 — Source data Fig. 1 [file 44319_2026_782_MOESM3_ESM.zip › Figure 1/1D/input northern_TER1.tif]

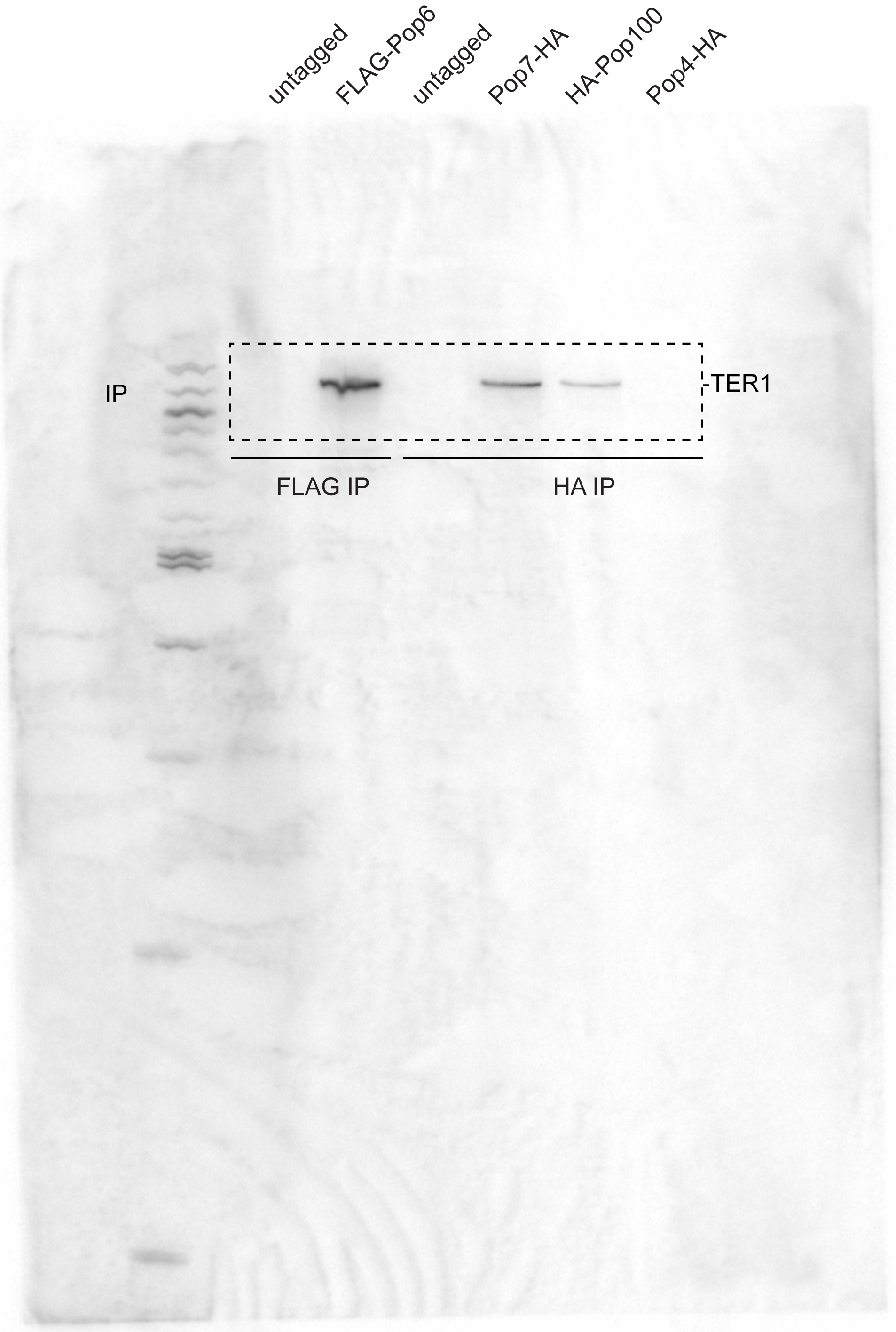

Supplement: Supplementary file 3 — Source data Fig. 1 [file 44319_2026_782_MOESM3_ESM.zip › Figure 1/1D/IP northern_TER1.tif]

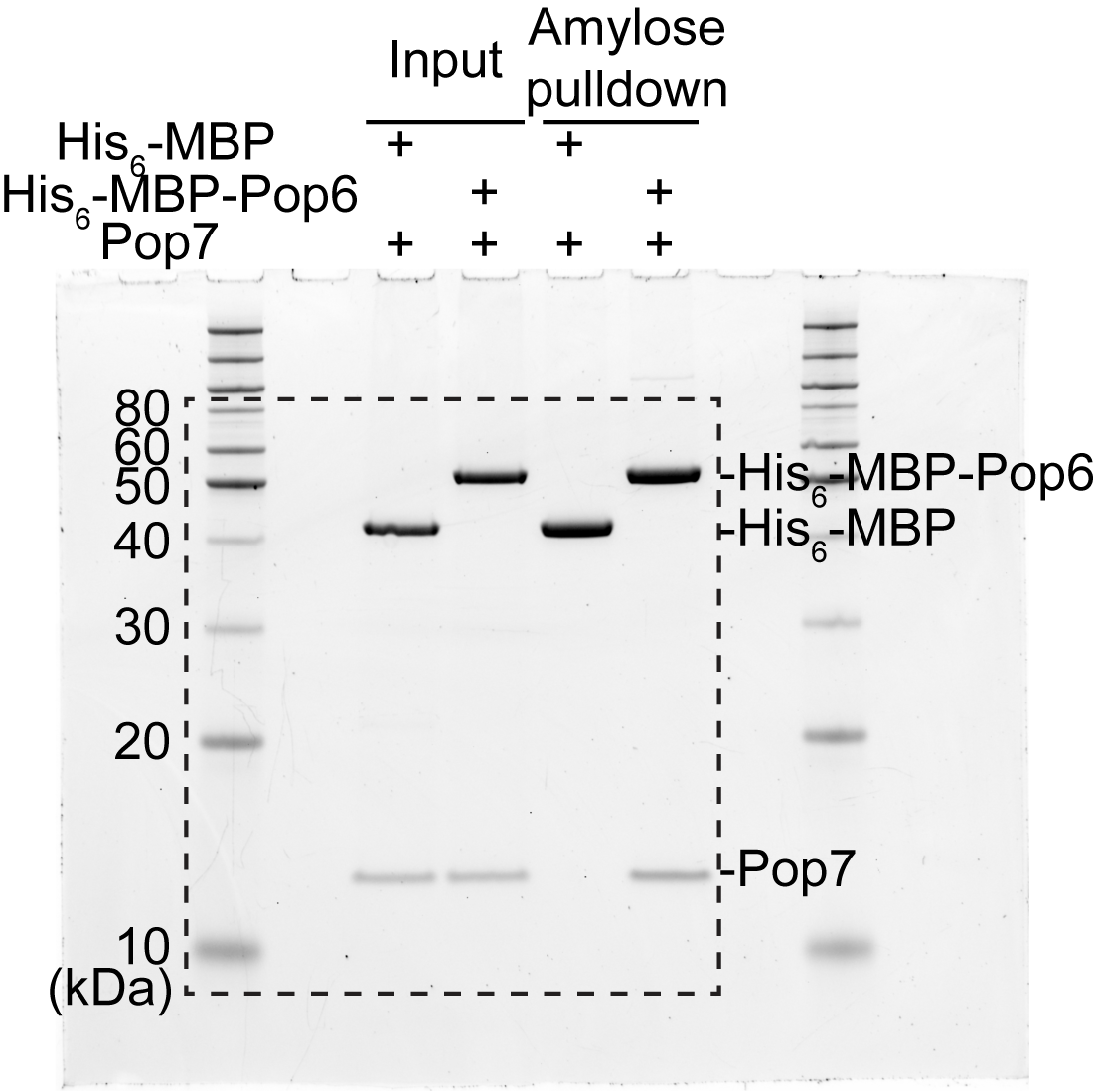

Supplement: Supplementary file 3 — Source data Fig. 1 [file 44319_2026_782_MOESM3_ESM.zip › Figure 1/1C/gel_Coomassie.tif]

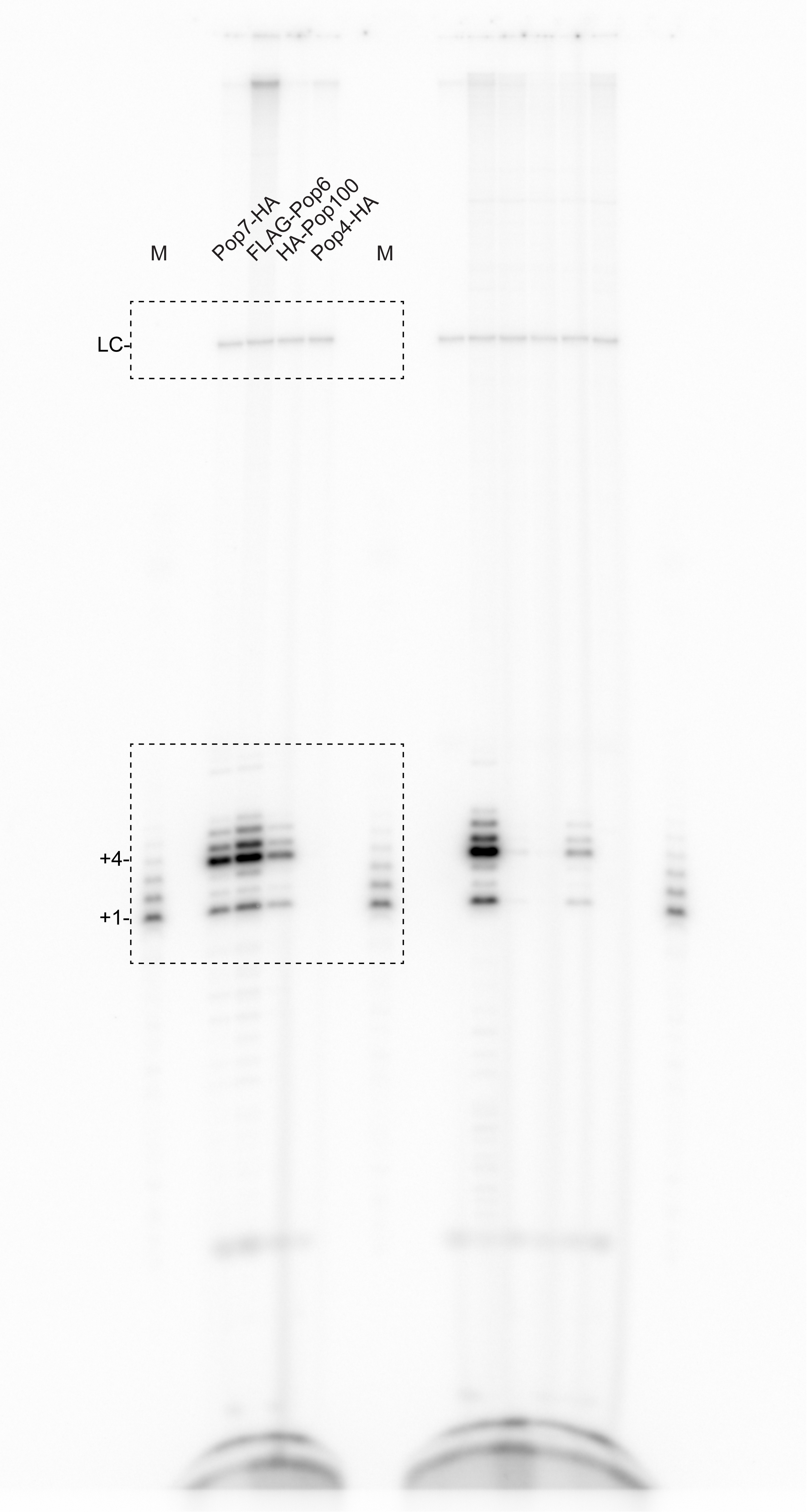

Supplement: Supplementary file 4 — Source data Fig. 2 [file 44319_2026_782_MOESM4_ESM.zip › Figure 2/2A/Telomerase activity assay.tif]

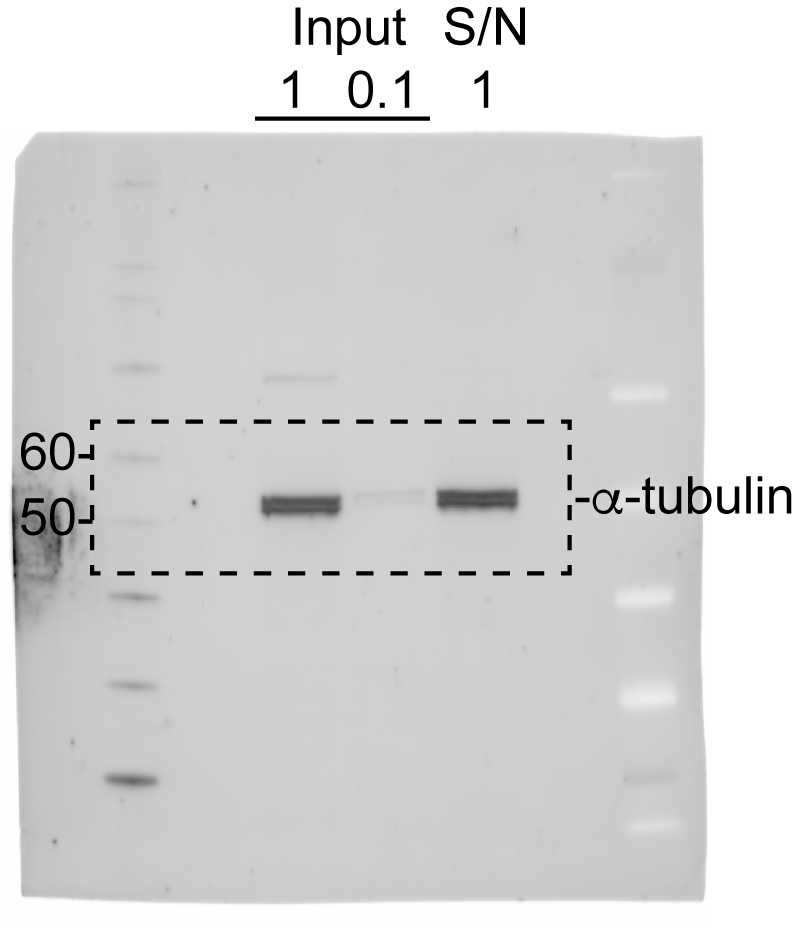

Supplement: Supplementary file 4 — Source data Fig. 2 [file 44319_2026_782_MOESM4_ESM.zip › Figure 2/2F/western_tbulin reprobe.tif]

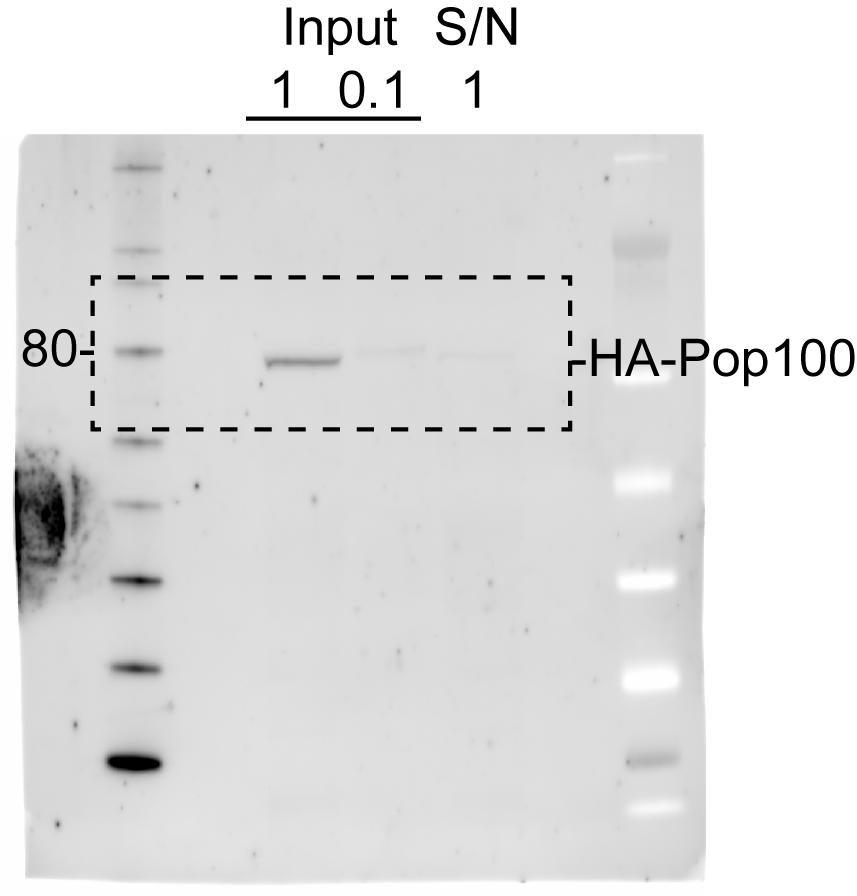

Supplement: Supplementary file 4 — Source data Fig. 2 [file 44319_2026_782_MOESM4_ESM.zip › Figure 2/2F/western_HA.tif]

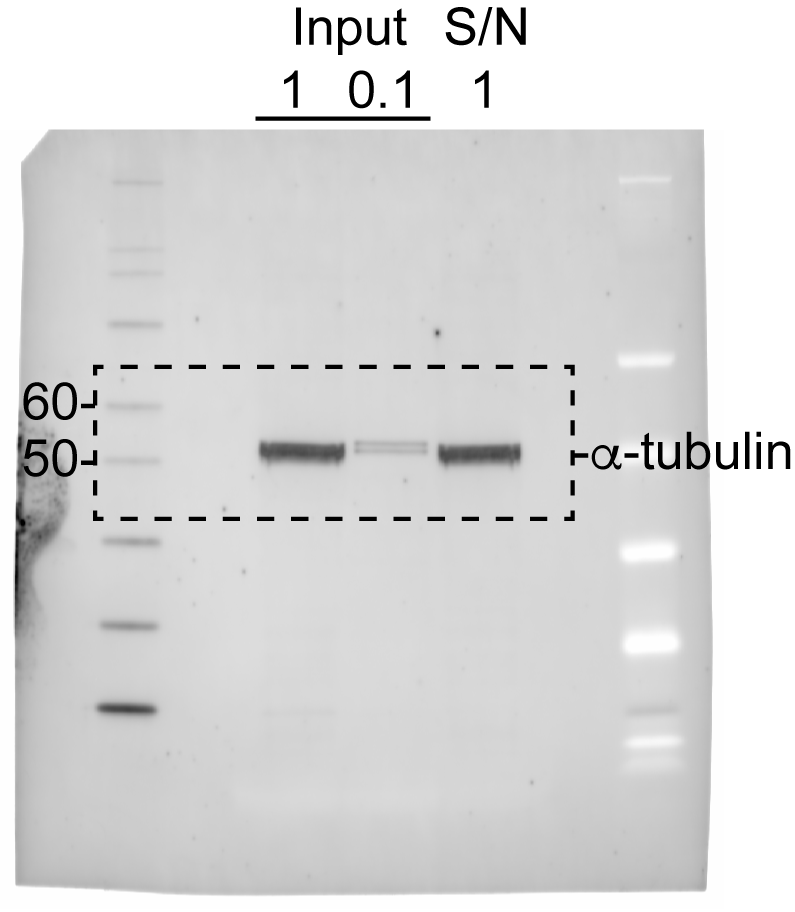

Supplement: Supplementary file 4 — Source data Fig. 2 [file 44319_2026_782_MOESM4_ESM.zip › Figure 2/2D/western_tubulin reprobe.tif]

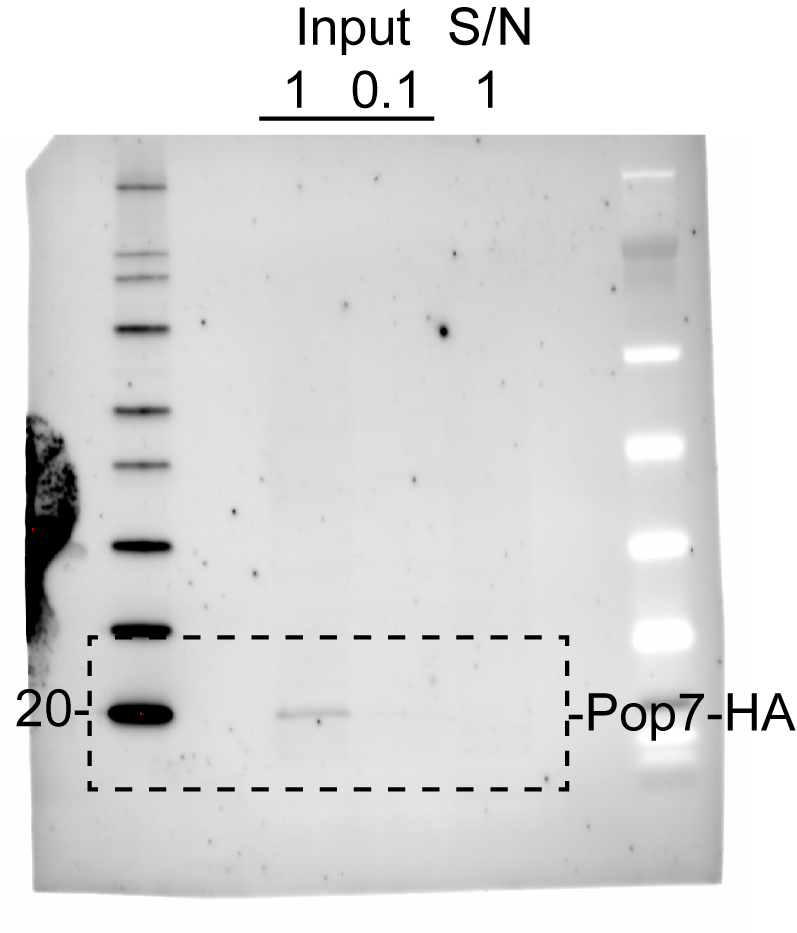

Supplement: Supplementary file 4 — Source data Fig. 2 [file 44319_2026_782_MOESM4_ESM.zip › Figure 2/2D/western_HA.tif]

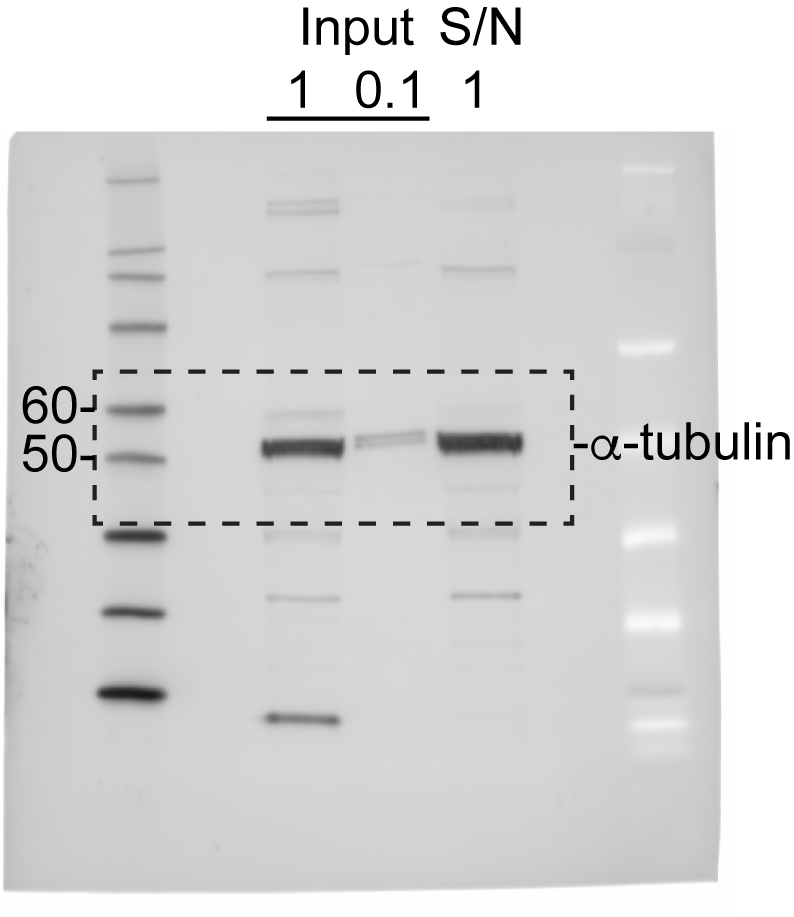

Supplement: Supplementary file 4 — Source data Fig. 2 [file 44319_2026_782_MOESM4_ESM.zip › Figure 2/2B/western_tubulin reprobe.tif]

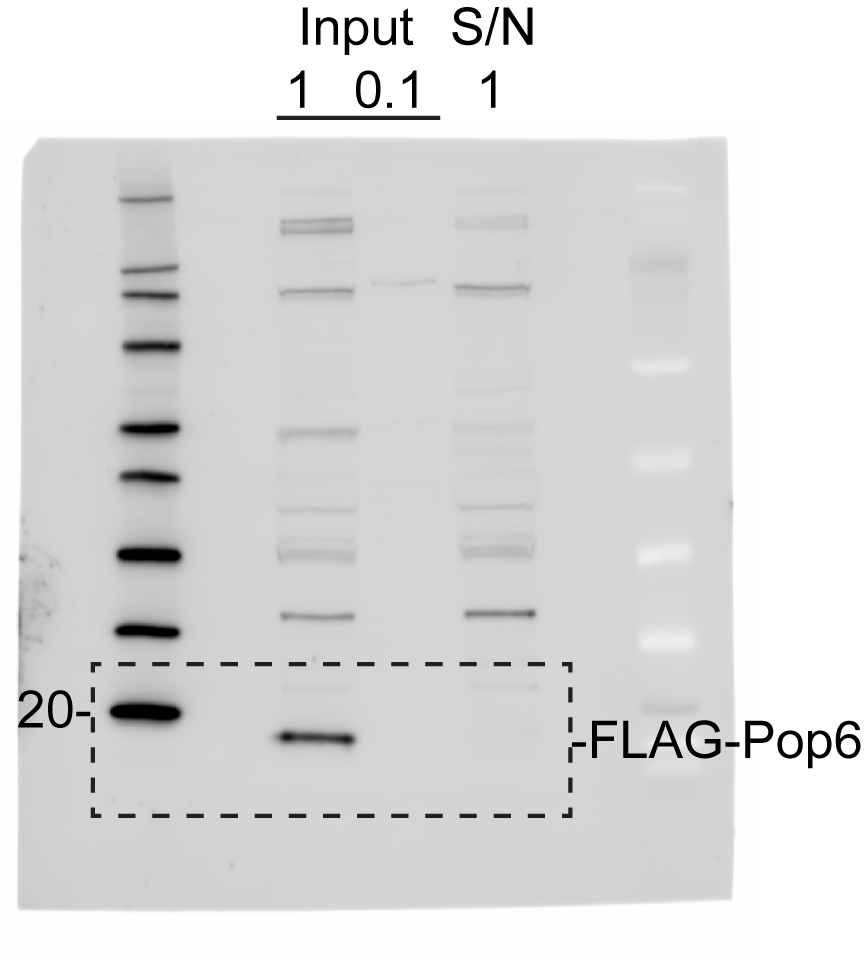

Supplement: Supplementary file 4 — Source data Fig. 2 [file 44319_2026_782_MOESM4_ESM.zip › Figure 2/2B/western_FLAG.tif]

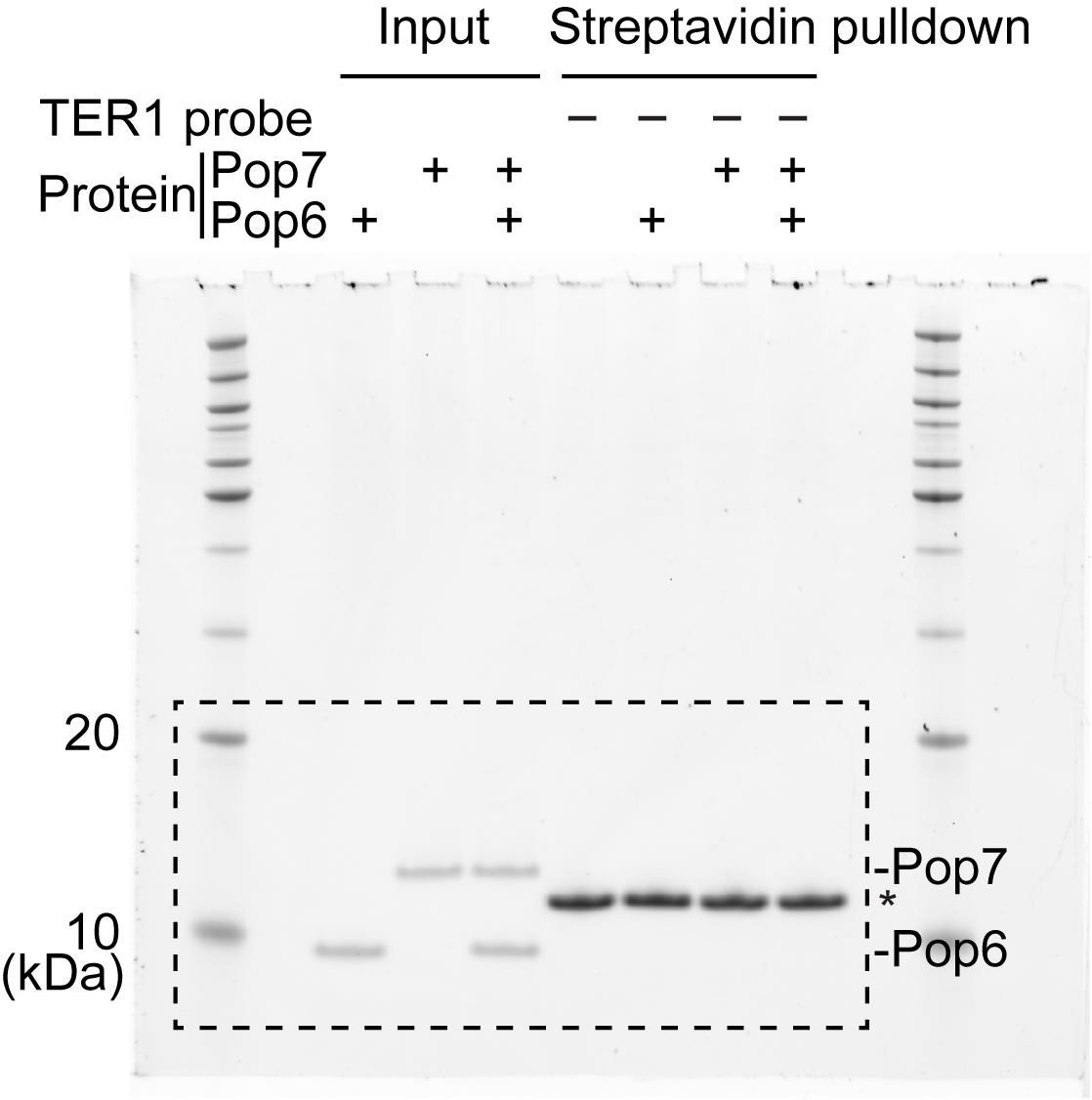

Supplement: Supplementary file 5 — Source data Fig. 3 [file 44319_2026_782_MOESM5_ESM.zip › Figure 3/3C/gel_Coomasie_1.tif]

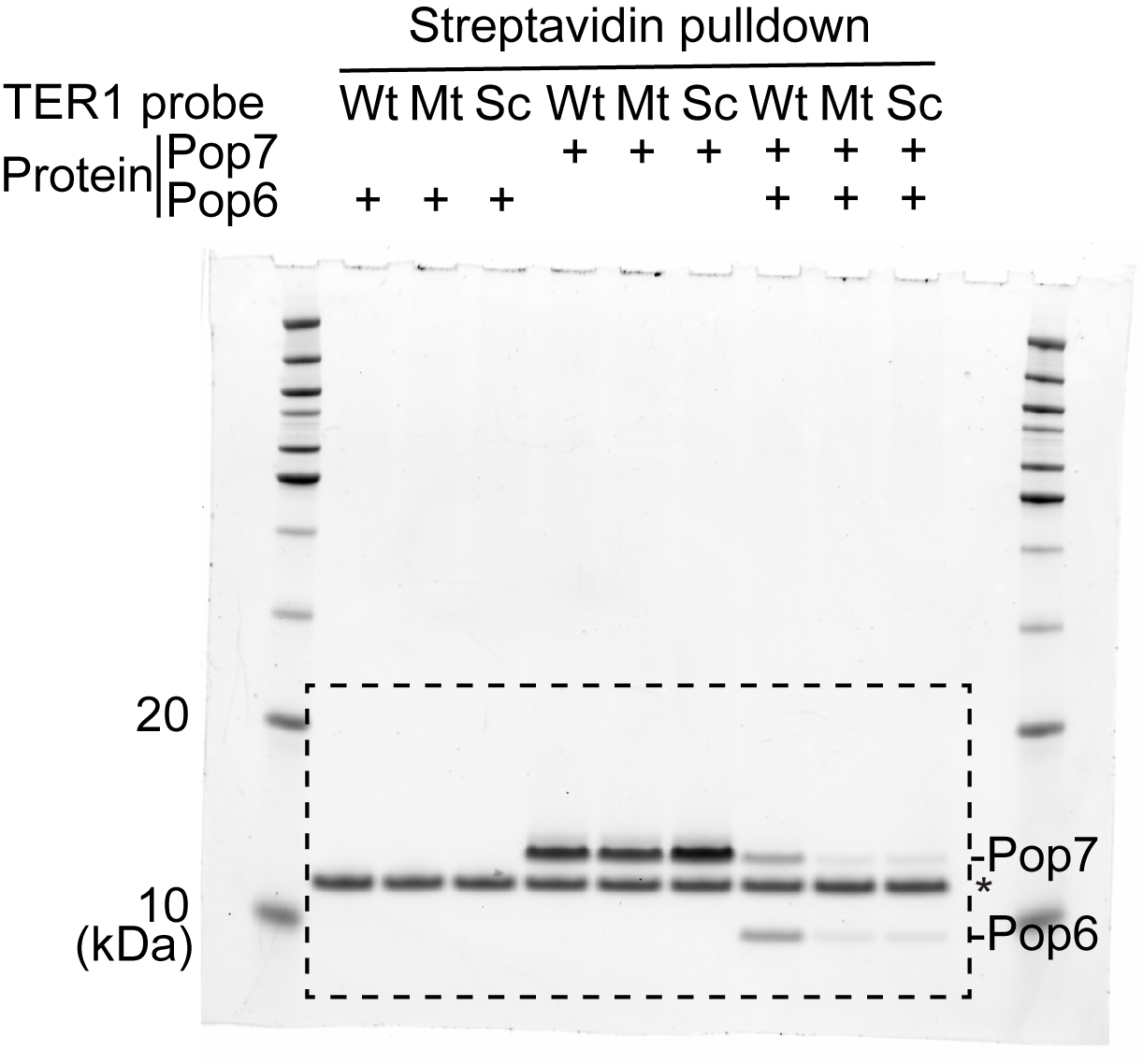

Supplement: Supplementary file 5 — Source data Fig. 3 [file 44319_2026_782_MOESM5_ESM.zip › Figure 3/3C/gel_Coomasie_2.tif]

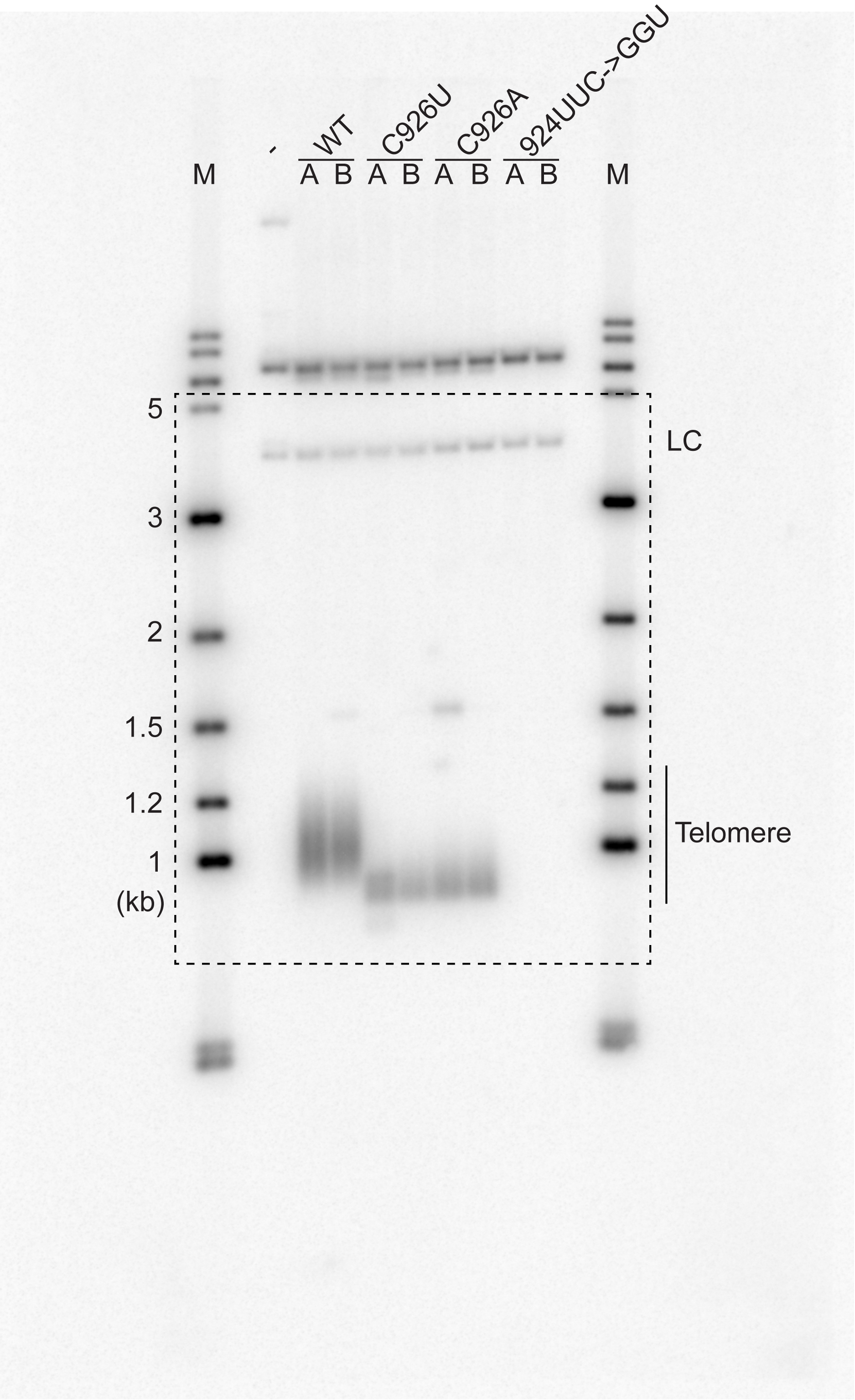

Supplement: Supplementary file 6 — Source data Fig. 4 [file 44319_2026_782_MOESM6_ESM.zip › Figure 4/4A/Telomeric Southern.tif]

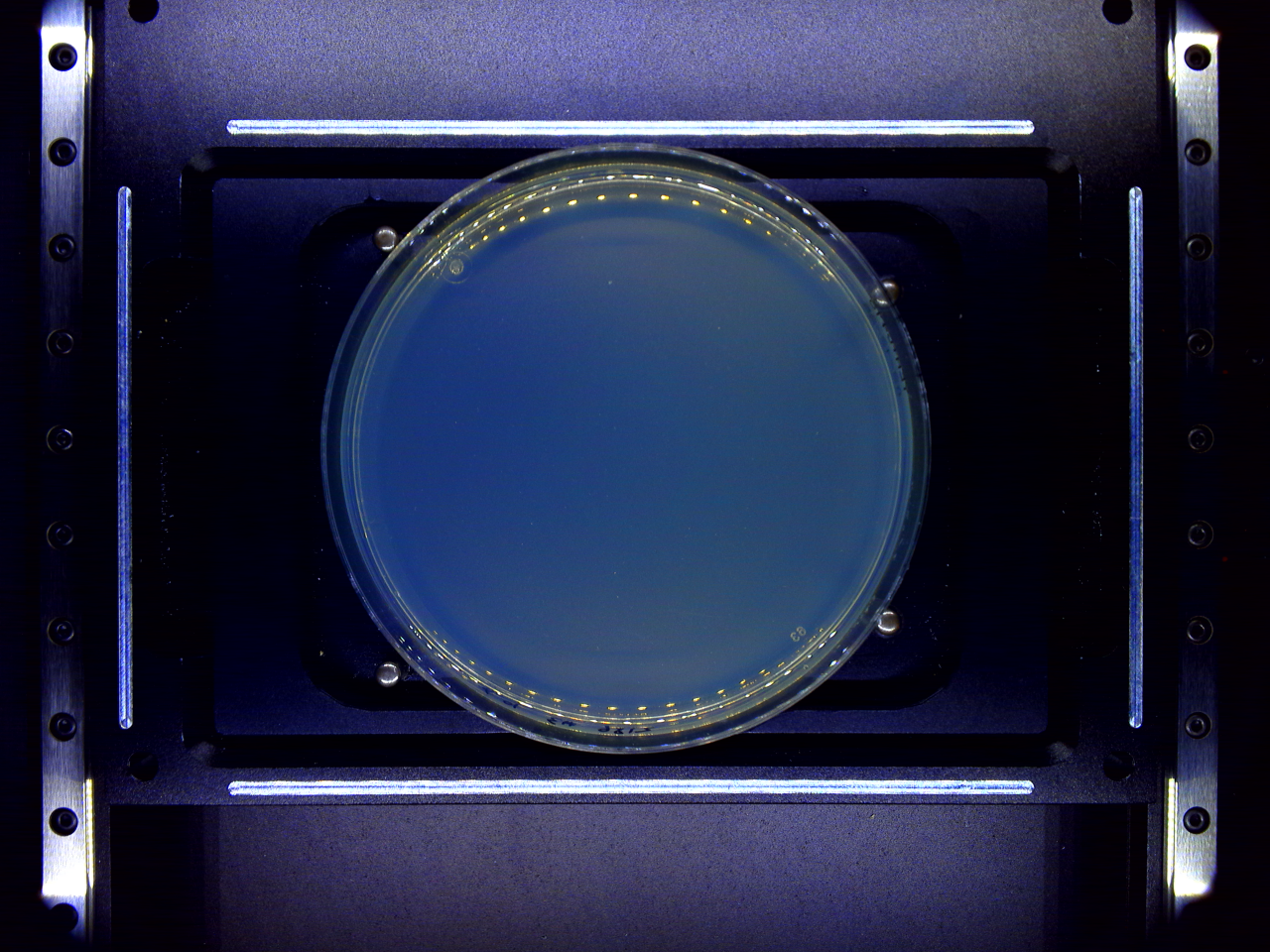

Supplement: Supplementary file 10 — Source Data Appendix S2B [file 44319_2026_782_MOESM10_ESM.zip › Source data_Appendix S2B/A_G418_dilution 2.png]

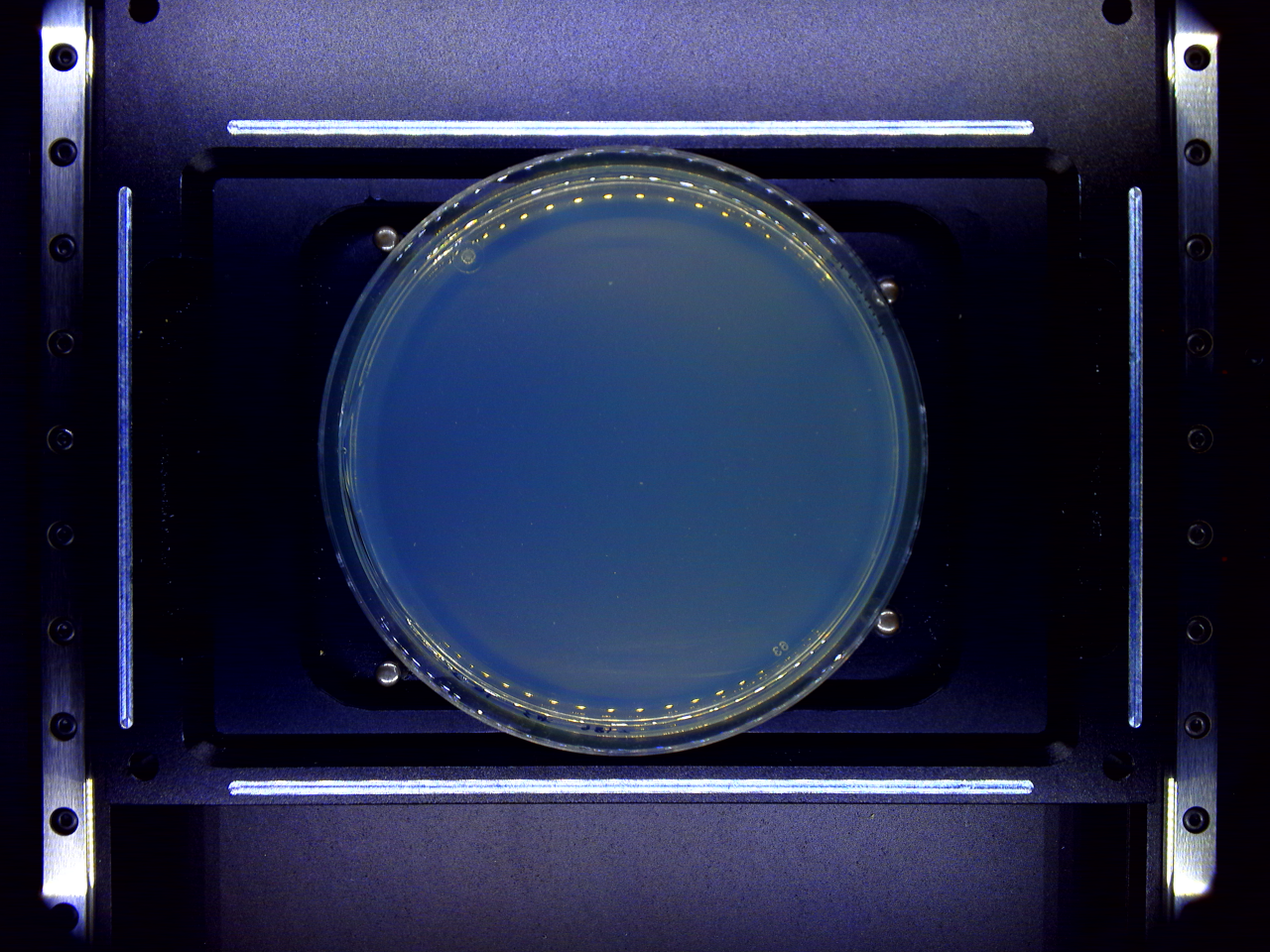

Supplement: Supplementary file 10 — Source Data Appendix S2B [file 44319_2026_782_MOESM10_ESM.zip › Source data_Appendix S2B/A_G418_dilution 3.png]

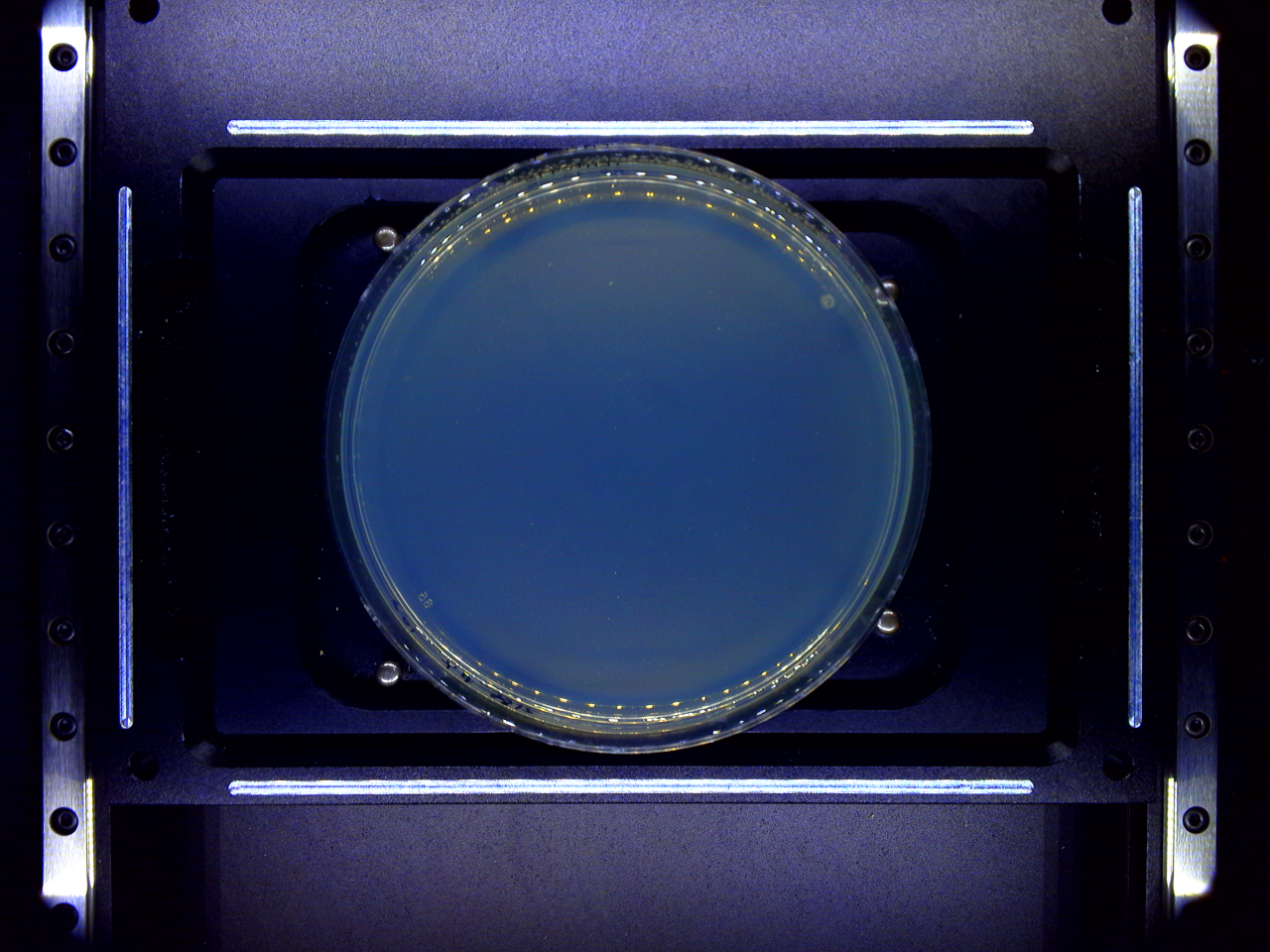

Supplement: Supplementary file 10 — Source Data Appendix S2B [file 44319_2026_782_MOESM10_ESM.zip › Source data_Appendix S2B/B_G418_dilution 1.png]

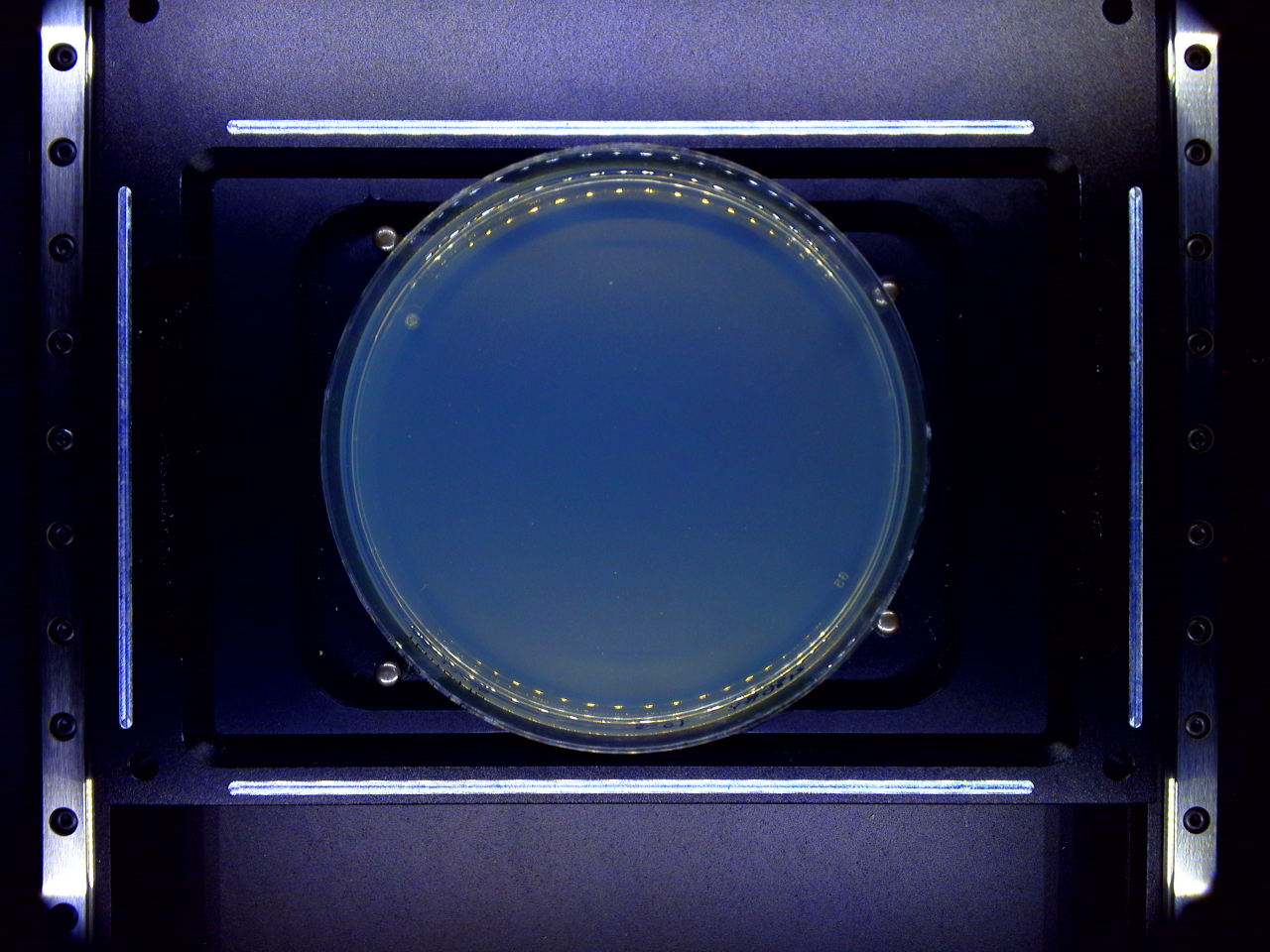

Supplement: Supplementary file 10 — Source Data Appendix S2B [file 44319_2026_782_MOESM10_ESM.zip › Source data_Appendix S2B/B_G418_dilution 3.png]

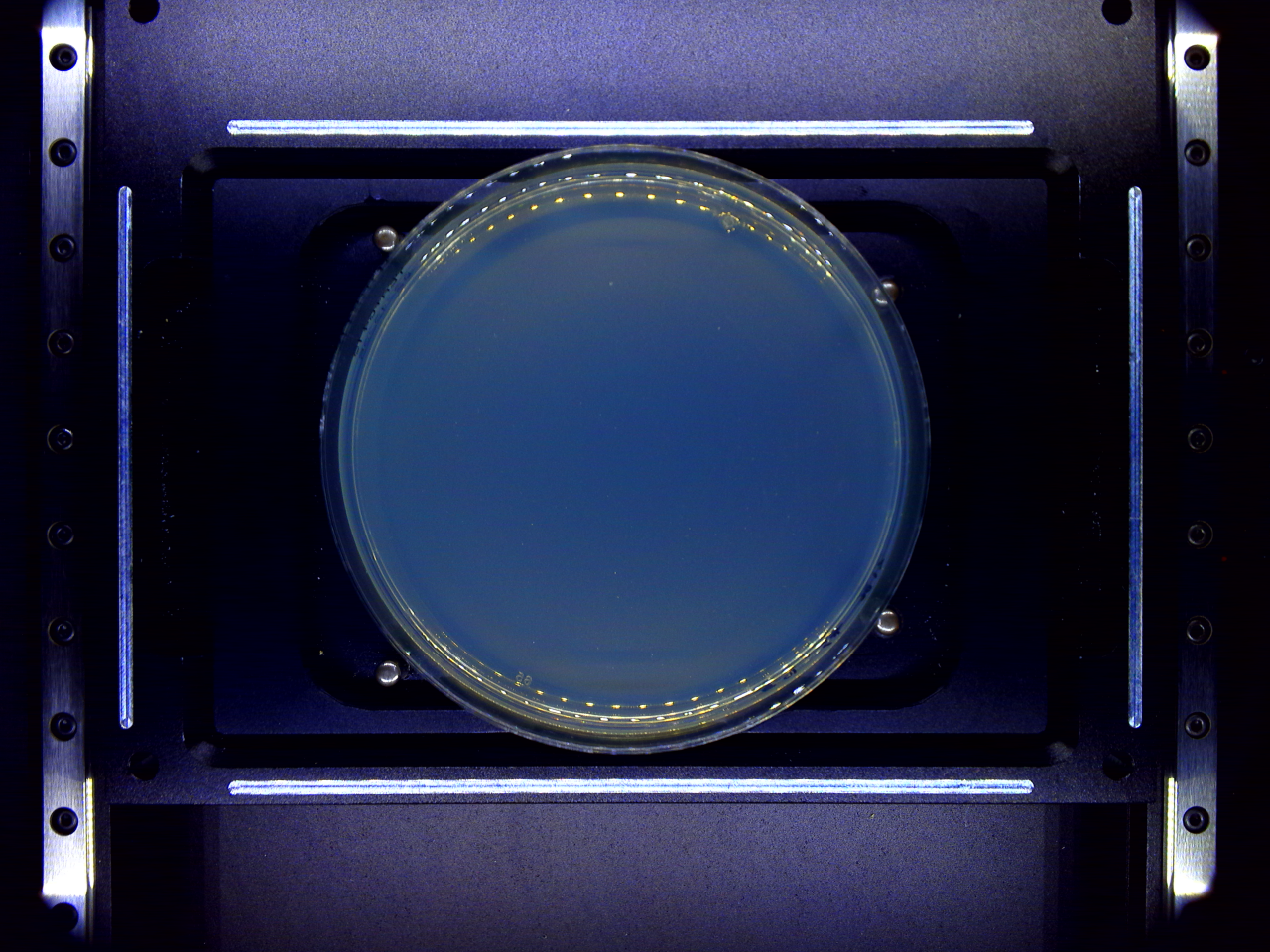

Supplement: Supplementary file 10 — Source Data Appendix S2B [file 44319_2026_782_MOESM10_ESM.zip › Source data_Appendix S2B/A_G418_dilution 1.png]

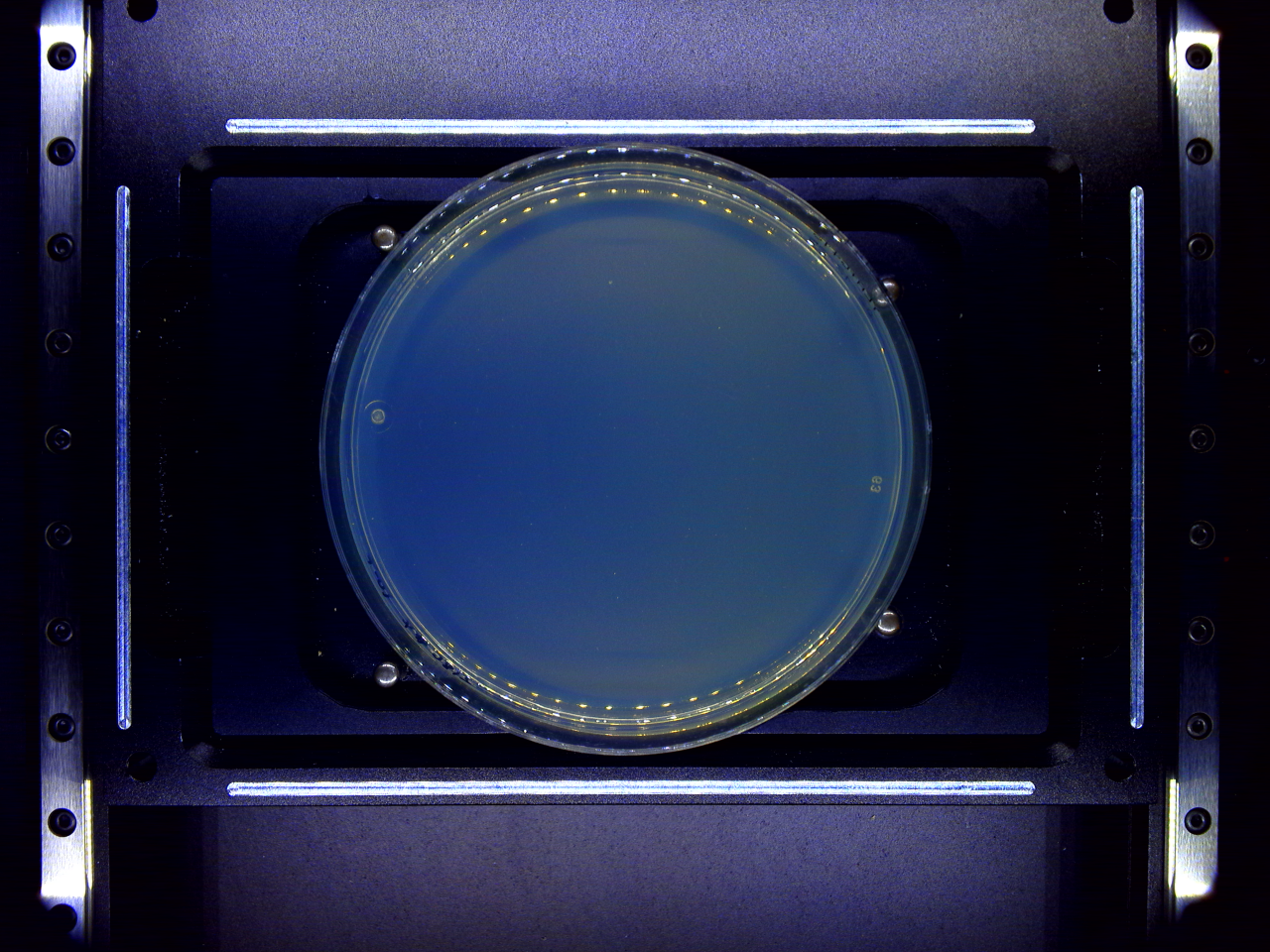

Supplement: Supplementary file 10 — Source Data Appendix S2B [file 44319_2026_782_MOESM10_ESM.zip › Source data_Appendix S2B/B_G418_dilution 2.png]

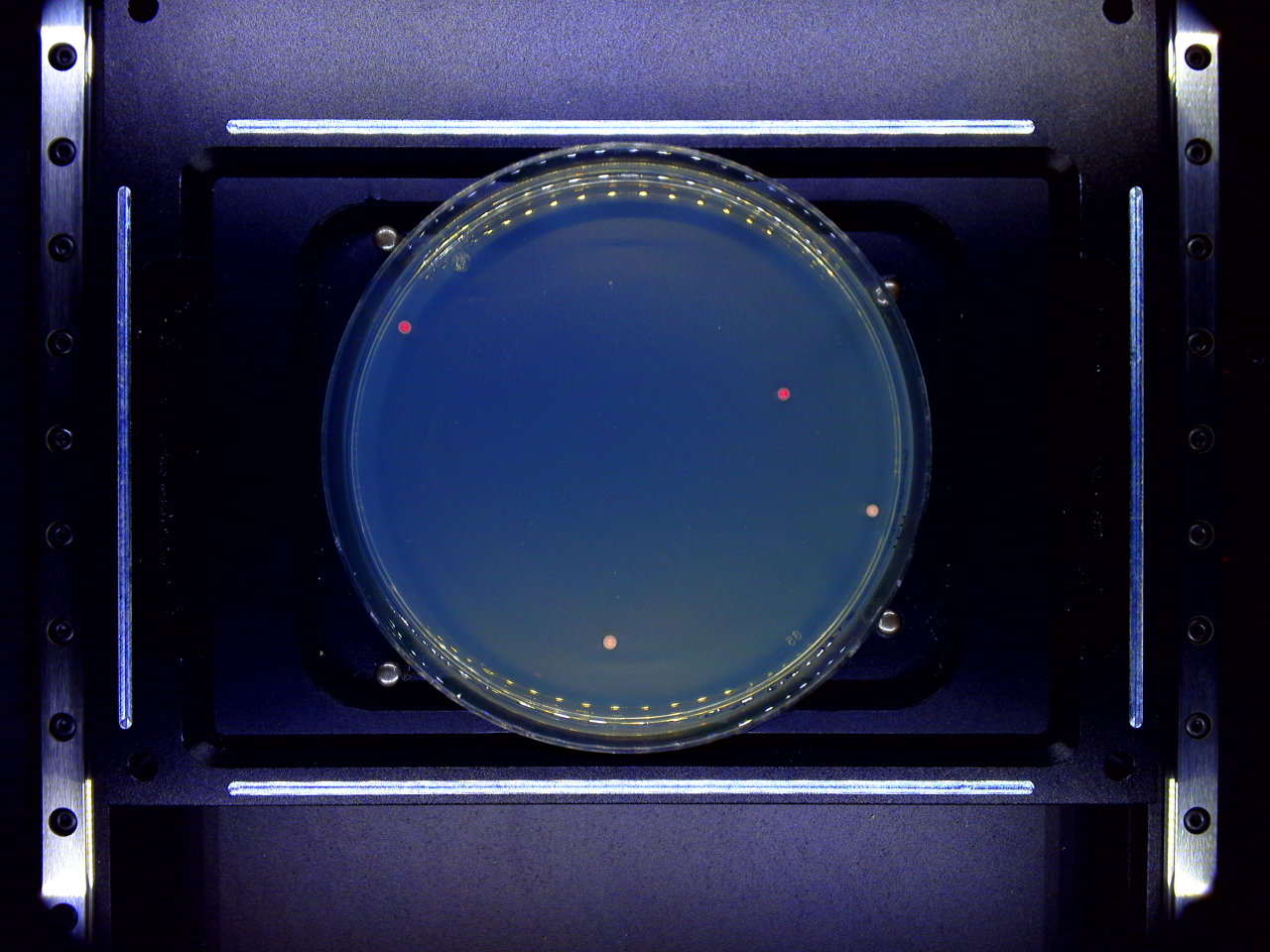

Supplement: Supplementary file 10 — Source Data Appendix S2B [file 44319_2026_782_MOESM10_ESM.zip › Source data_Appendix S2B/A_YEA_dilution 3.png]

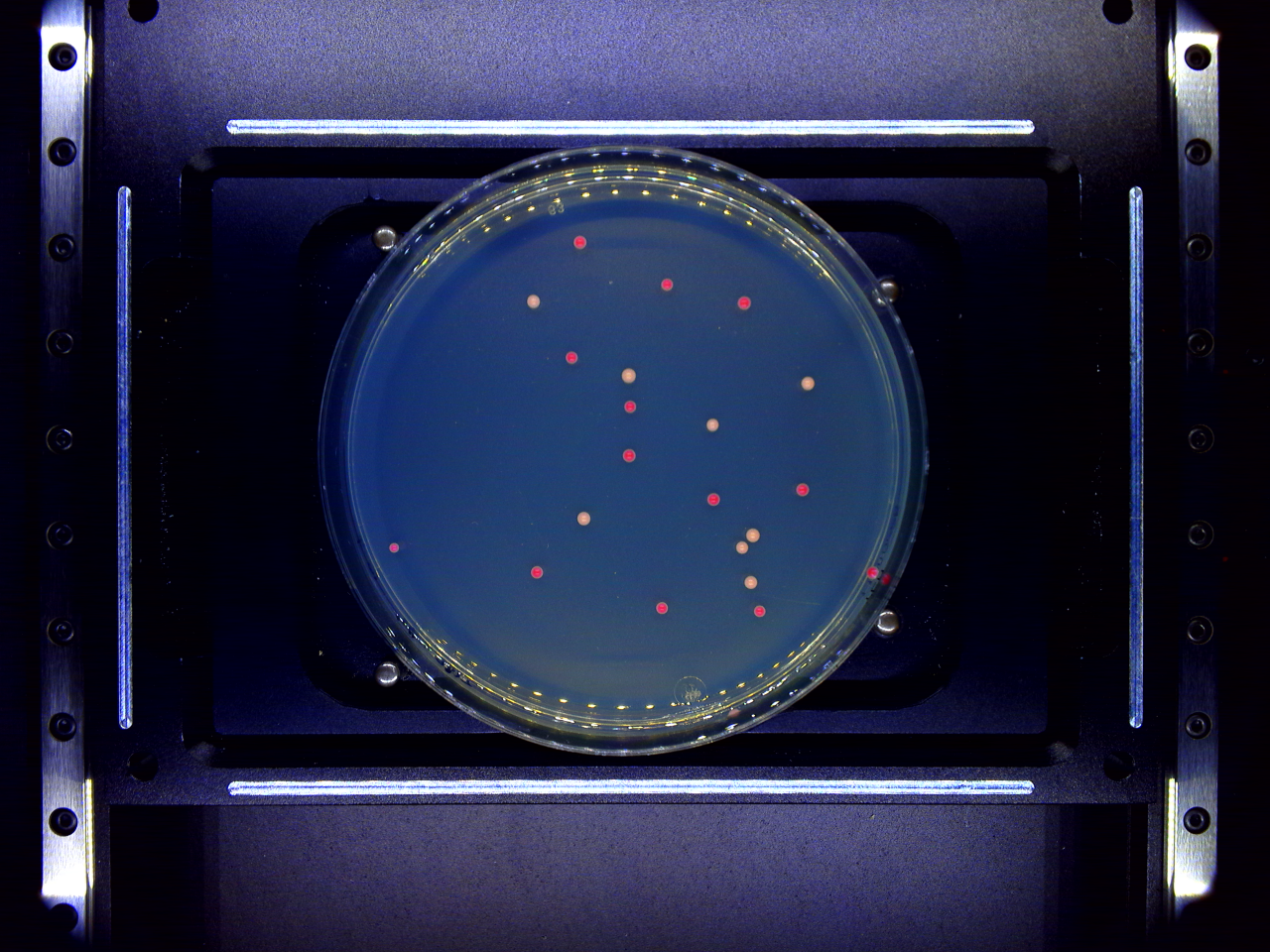

Supplement: Supplementary file 10 — Source Data Appendix S2B [file 44319_2026_782_MOESM10_ESM.zip › Source data_Appendix S2B/A_YEA_dilution 2.png]

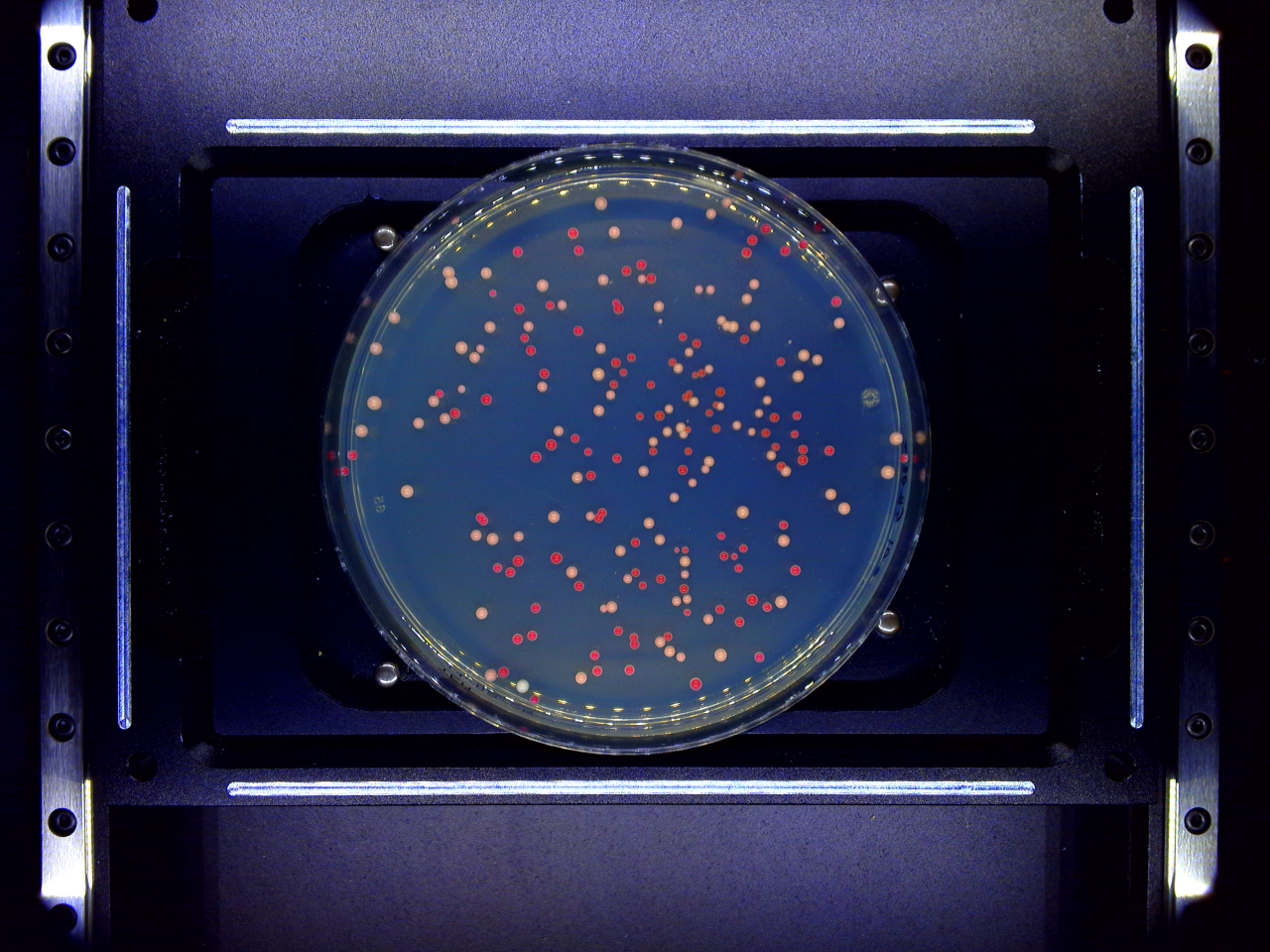

Supplement: Supplementary file 10 — Source Data Appendix S2B [file 44319_2026_782_MOESM10_ESM.zip › Source data_Appendix S2B/A_YEA_dilution 1.png]

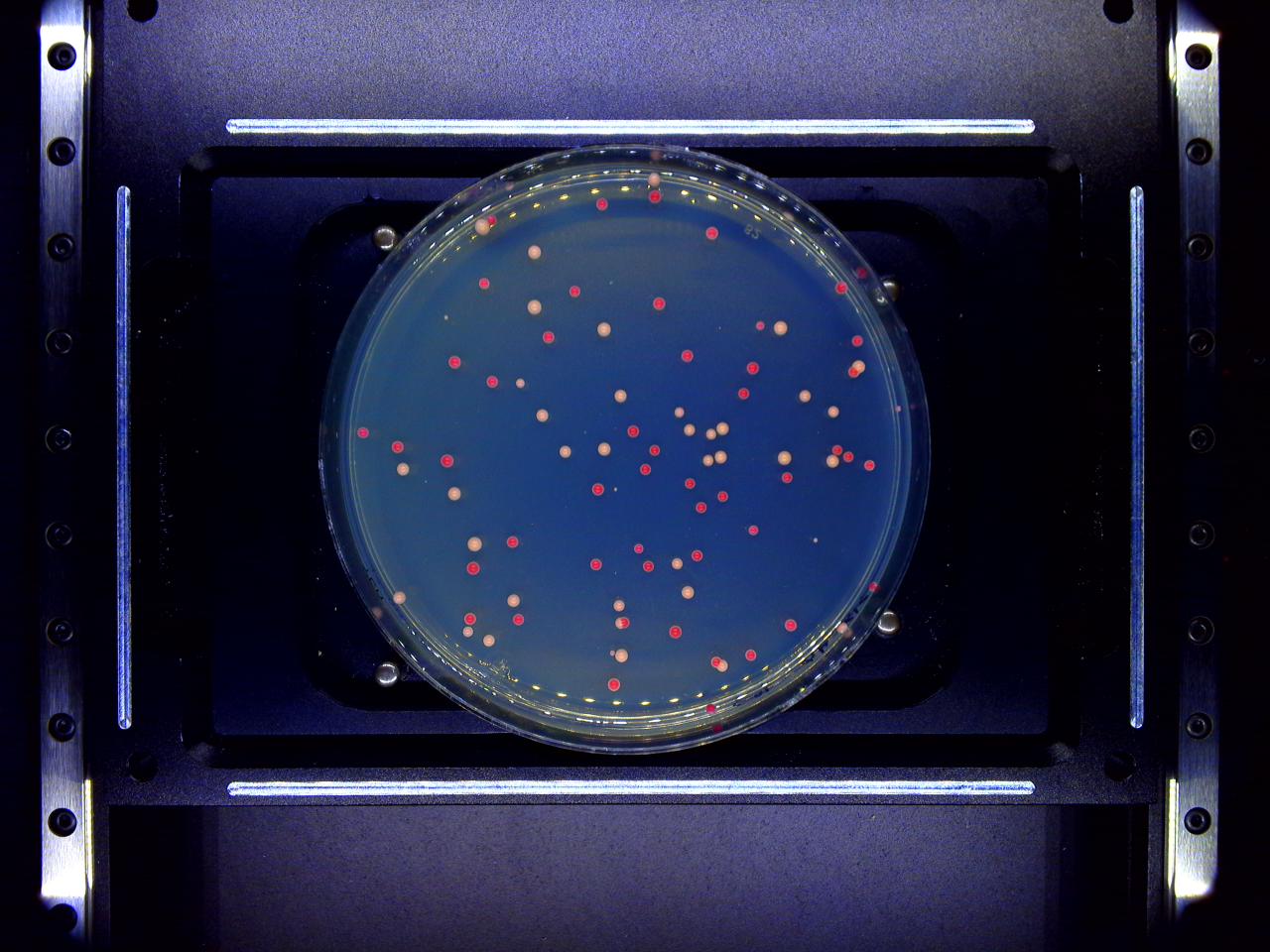

Supplement: Supplementary file 10 — Source Data Appendix S2B [file 44319_2026_782_MOESM10_ESM.zip › Source data_Appendix S2B/B_YEA_dilution 1.png]

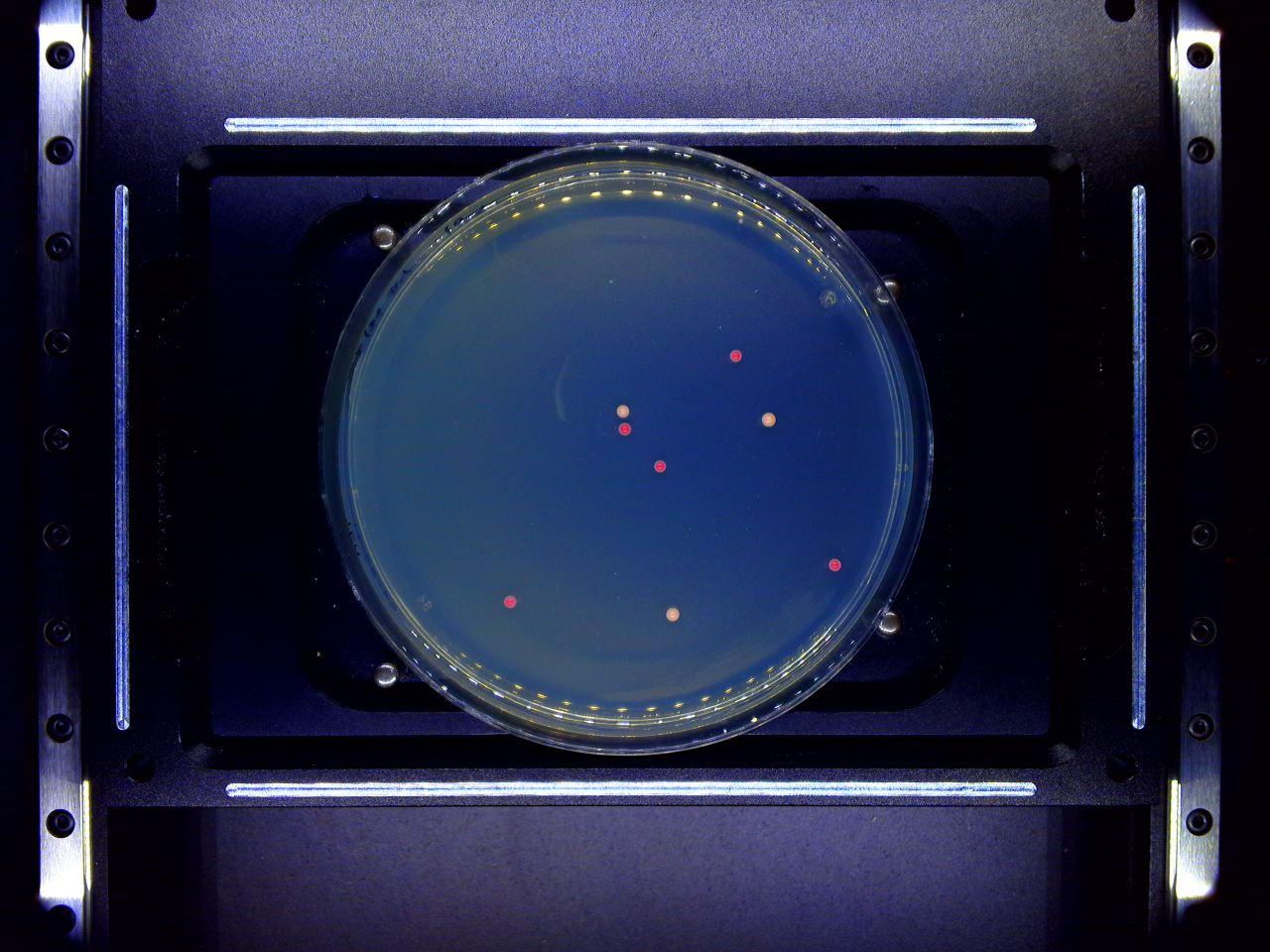

Supplement: Supplementary file 10 — Source Data Appendix S2B [file 44319_2026_782_MOESM10_ESM.zip › Source data_Appendix S2B/B_YEA_dilution 2.png]

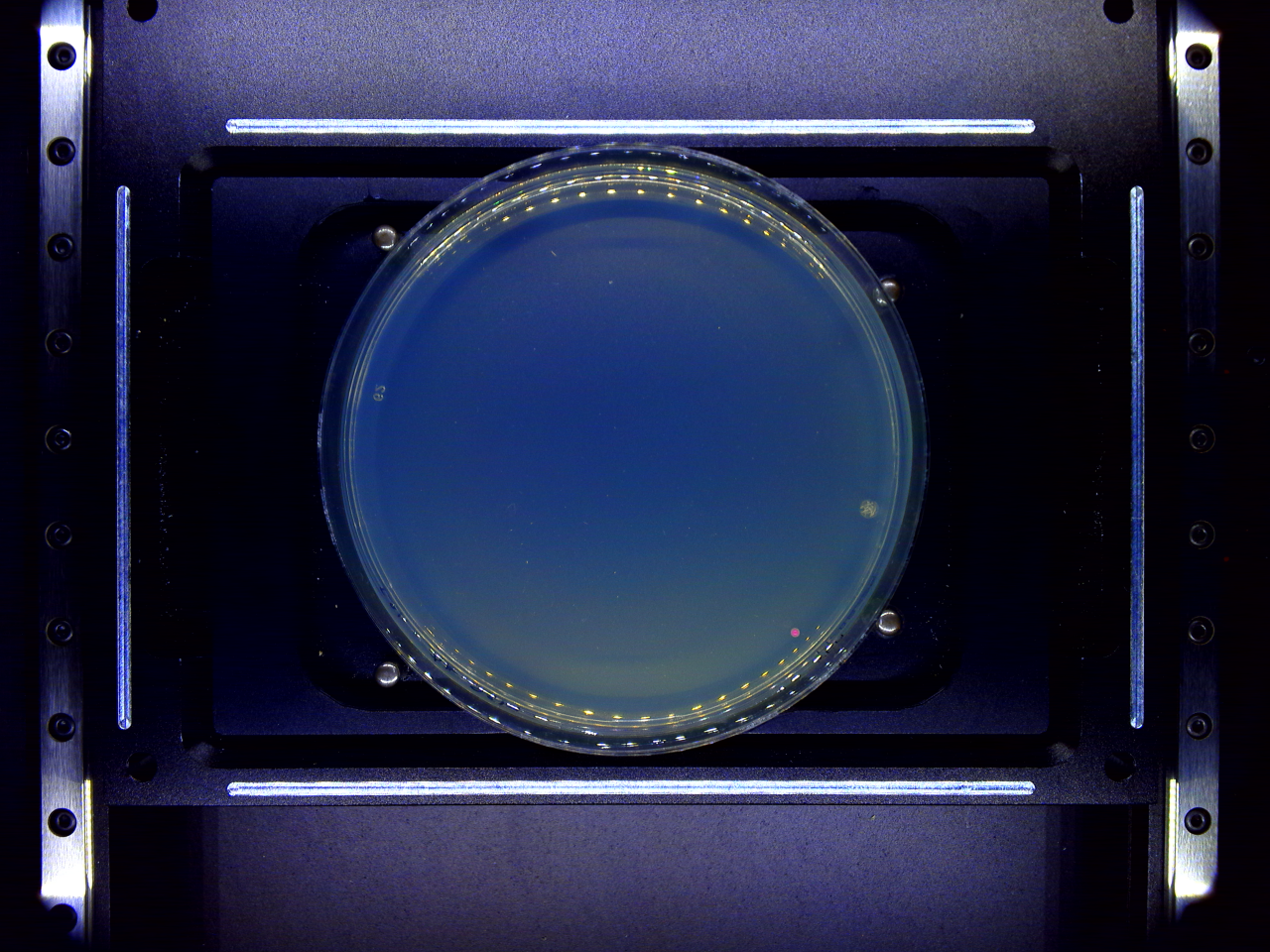

Supplement: Supplementary file 10 — Source Data Appendix S2B [file 44319_2026_782_MOESM10_ESM.zip › Source data_Appendix S2B/B_YEA_dilution 3.png]
