# Supplementary material for: RNase P/MRP subunits chaperone telomerase holoenzyme assembly in fission yeast
Source: EMBO Rep. 2026 Apr 28;27(12):3277–302. doi: 10.1038/s44319-026-00782-9 (PMC13303942; doi:10.1038/s44319-026-00782-9)
Supplement: Supplementary file 8 — Source data Fig. 6 [file 44319_2026_782_MOESM8_ESM.zip › Figure 6/README.rtf]

The source data for this figure has been uploaded to the public database https://www.ncbi.nlm.nih.gov/bioproject/ under the accession number PRJNA1250409. 
