# Supplementary material for: RNase P/MRP subunits chaperone telomerase holoenzyme assembly in fission yeast
Source: EMBO Rep. 2026 Apr 28;27(12):3277–302. doi: 10.1038/s44319-026-00782-9 (PMC13303942; doi:10.1038/s44319-026-00782-9)
Supplement: Supplementary file 9 — Source data Fig. 7 [file 44319_2026_782_MOESM9_ESM.zip › Figure 7/7A and 7B/README.rtf]

The source data for these figures has been uploaded to the public database https://www.ncbi.nlm.nih.gov/bioproject/ under the accession number PRJNA1250409. 
