# Supplementary material for: RNase P/MRP subunits chaperone telomerase holoenzyme assembly in fission yeast
Source: EMBO Rep. 2026 Apr 28;27(12):3277–302. doi: 10.1038/s44319-026-00782-9 (PMC13303942; doi:10.1038/s44319-026-00782-9)
Supplement: Supplementary file 11 — Expanded View Figures [file 44319_2026_782_MOESM11_ESM.pdf]

Expanded View Figures

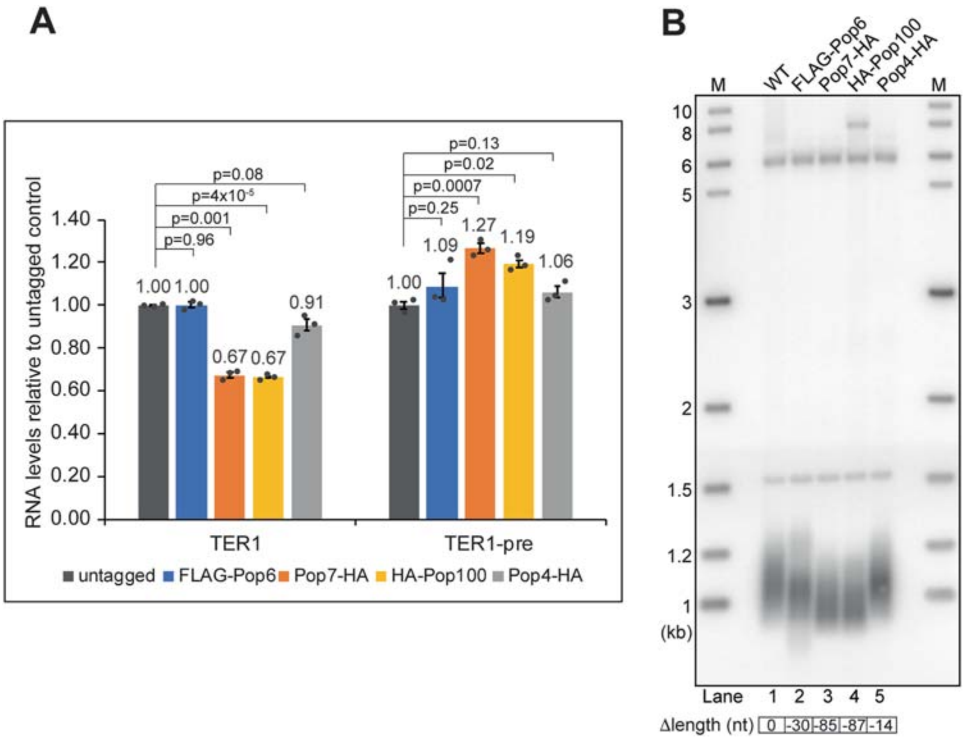

Figure EV1. Effect of tagging Pop6, Pop7, Pop100, and Pop4 on telomere maintenance.

(**A**) RT–qPCR quantification of TER1 RNA levels in strains expressing tagged Pop proteins under their endogenous promoters. Bars represent mean values (±SEM, $n = 3$) relative to the untagged control, normalized to reference genes. Statistical significance was assessed using unpaired t tests ($n = 3$). (**B**) Telomere length analysis by Southern blot in strains expressing tagged versions of Pop6, Pop7, Pop100, or Pop4. A probe against telomeric repeats was used for hybridization. Δlength indicates the difference in fragment length comparing each lane to lane 1 (WT).

 © The Author(s)

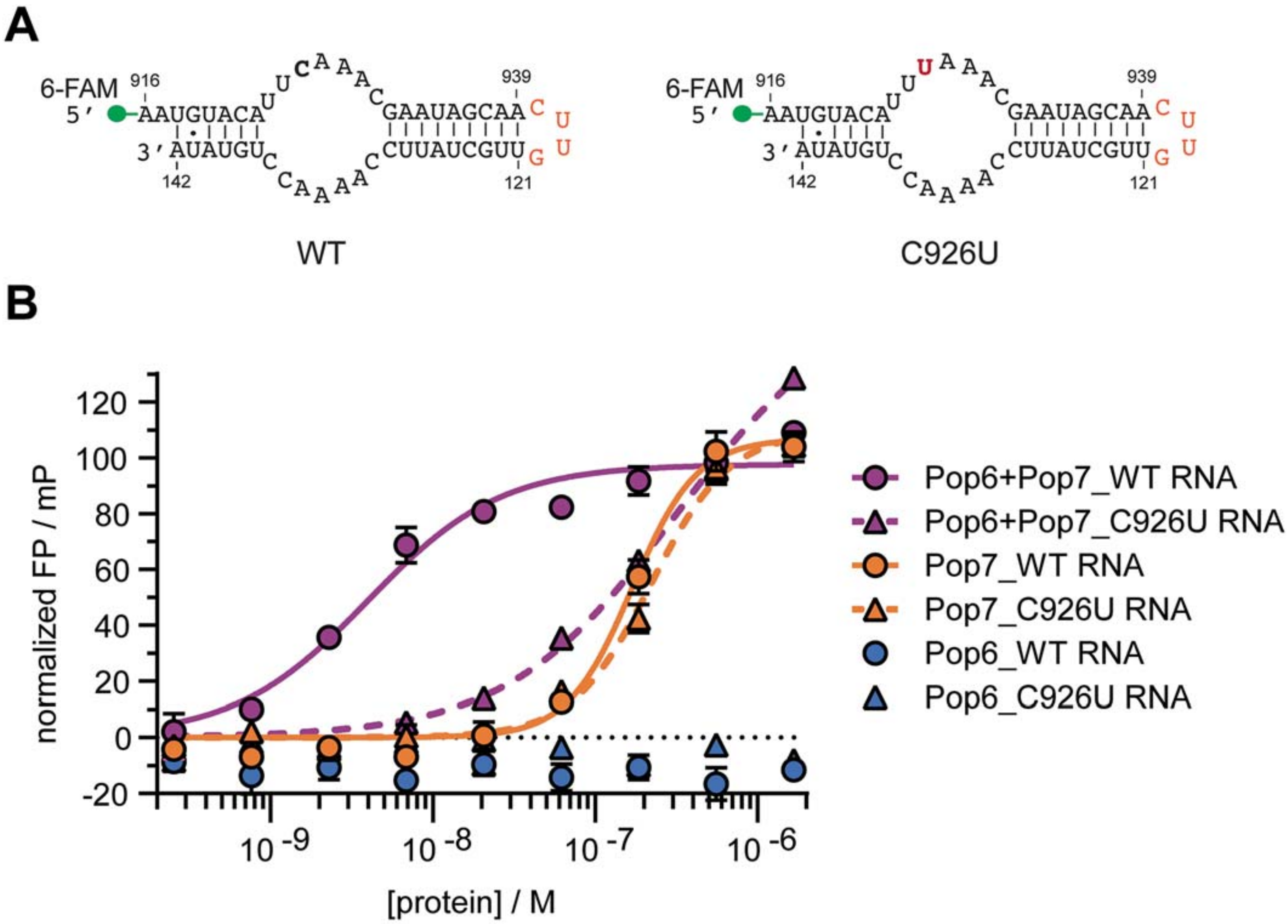

C

Protein	TER1 probe	Kd [95% CI]	Hill slope [95% CI]
Pop6+Pop7	WT	4 nM [3.0 nM, 5.6 nM]	1.06 [0.79, 1.47]
	C926U	309 nM [233 nM, 448 nM]	0.84 [0.74, 0.95]
Pop7	WT	169 nM [148 nM, 193 nM]	2.19 [1.71, 3.04]
	C926U	220 nM [192 nM, 256 nM]	1.79 [1.44, 2.31]
Pop6	WT	n.d.	n.d.
	C926U	n.d.	n.d.

Figure EV2. Fluorescence polarization measurements of Pop6, Pop7, or the complex binding to the TER1 P3-like domain in vitro.

(**A**) Schematic of 6-FAM-labeled TER1 P3-like domain probes used in the fluorescence polarization assays. The WT probe includes TER1 nts 916–939 and 121–142 connected by a CUUG linker (orange). The C926U-mutant probe contains a C926U mutation (red). (**B**) Fluorescence polarization (FP) assays measuring binding of 6-FAM-labeled RNA by various amounts of Pop6, Pop7, or a 1:1 mixture of both proteins. Mean values from triplicates including error bars representing standard deviations are shown for each measurement. (**C**) Dissociation constant (Kd) and Hill slope derived from nonlinear regression fits of the FP measurements with the upper and lower limits of 95% confidence interval (CI) indicated in the brackets.

© The Author(s)

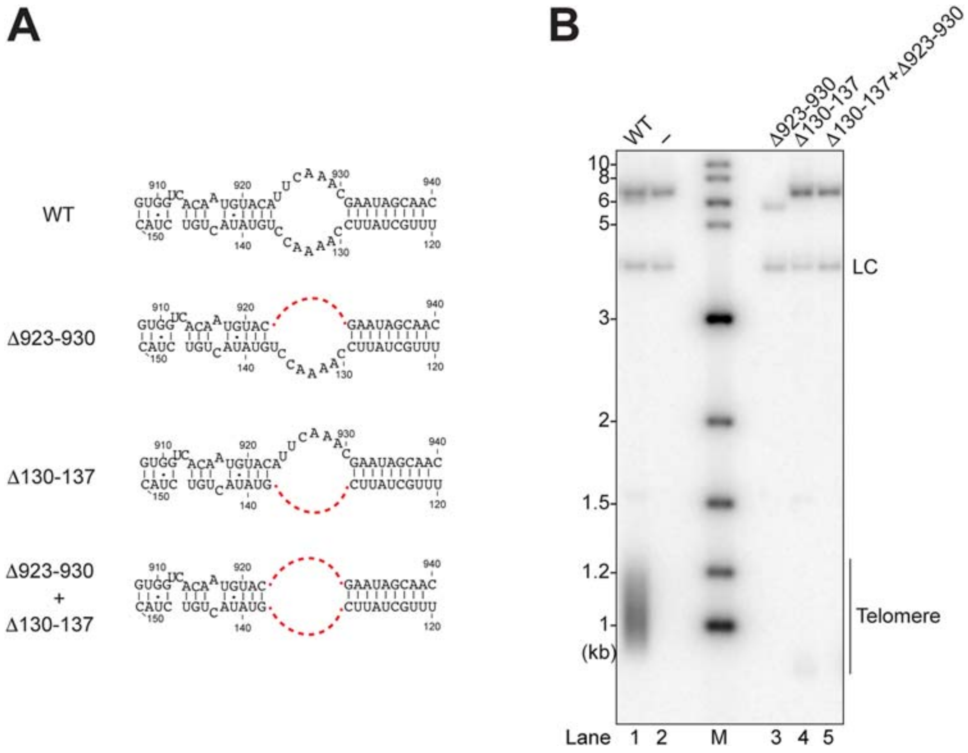

Figure EV3. Telomere length analysis of P3 loop deletion mutants.

(**A**) Schematic of P3 loop deletion mutants. Red dashed lines indicate the deleted RNA sequences. (**B**) Telomere length analysis of the deletion mutants shown in (**A**), expressed from a plasmid in *ter1Δ* cells and restreaked five times (~110 generations). Southern blot was performed using a probe against telomeric repeats and *rad16*+ as a loading control (LC). M: size ladder, with molecular weight markers indicated on the left.

 © The Author(s)

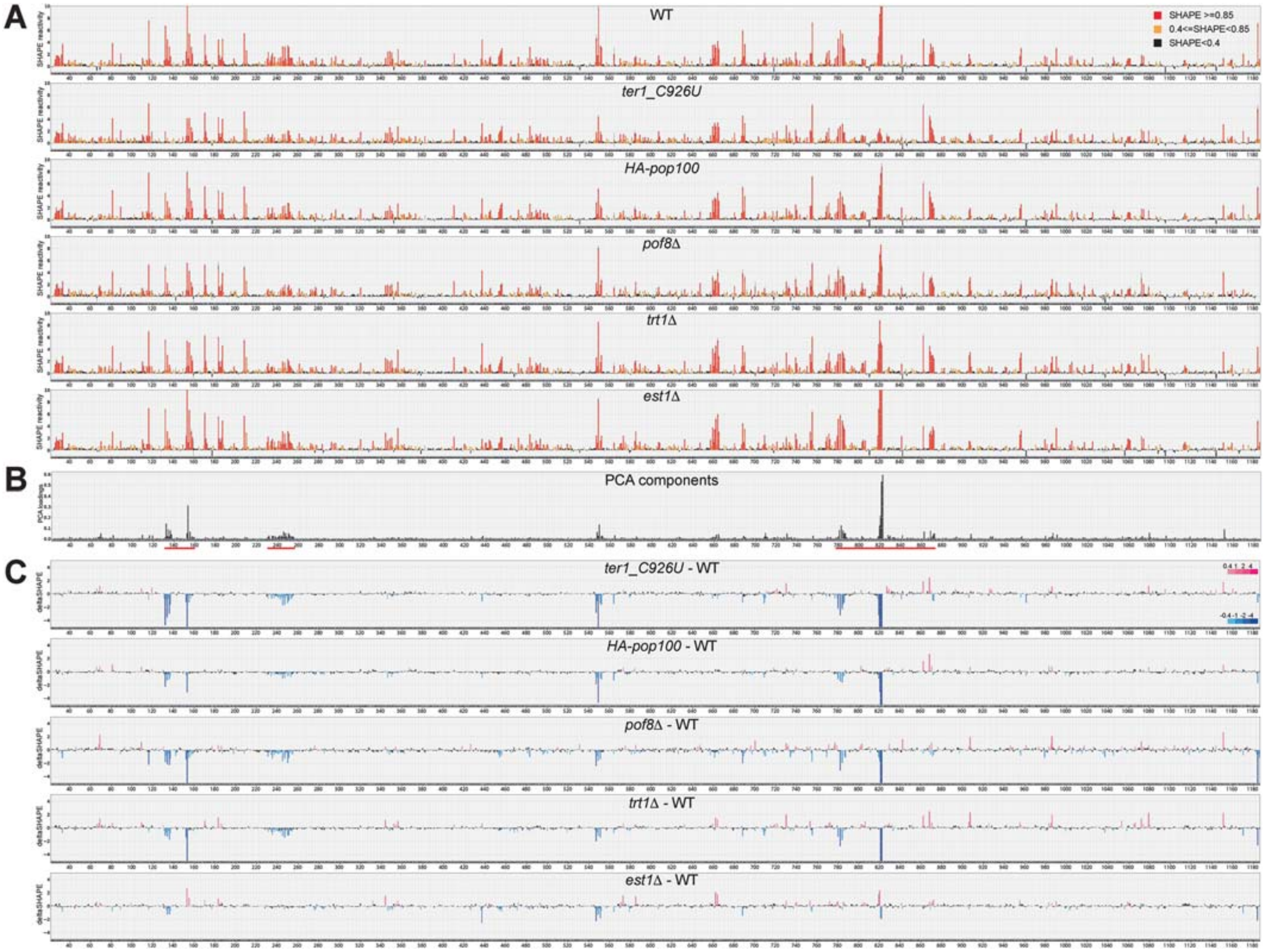

Figure EV4. In-cell SHAPE-MaP profiles of TER1 RNA.

(**A**) In-cell SHAPE-MaP analysis of TER1 in WT, *ter1_C926U*, *HA-pop100*, *pof8Δ*, *trt1Δ*, and *est1Δ* strains. Bars represent mean SHAPE reactivities (±SEM, $n = 3$), with a maximum display value of 10, across nucleotide positions 22–1186 (primer regions excluded). (**B**) PCA loadings for each nucleotide from the principal component analysis (PCA) of SHAPE data shown in (**A**). Underlined regions correspond to the P3-like and T–PK domains of TER1. (**C**) deltaSHAPE profiles comparing average SHAPE reactivities of *ter1_C926U*, *HA-pop100*, *pof8Δ*, *trt1Δ*, and *est1Δ*strains to WT (mutant minus WT). Nucleotides with deltaSHAPE > 0.4 are shown in pink, < –0.4 in blue, and between –0.4 and 0.4 in black.

© The Author(s)

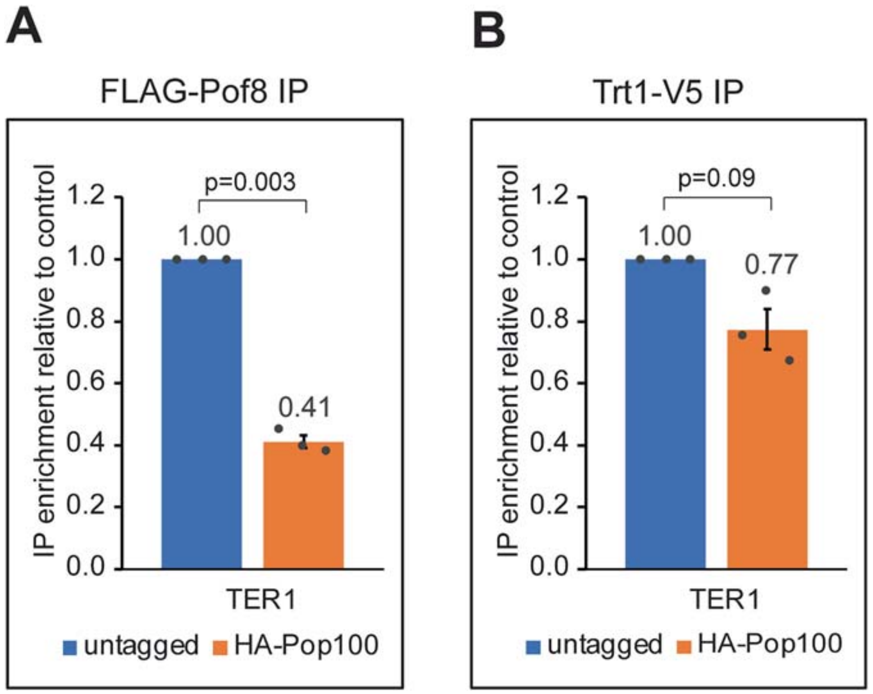

Figure EV5. Effect of HA-tagged Pop100 on Pof8 and Trt1 binding to TER1.

(**A**) RT–qPCR analysis of RNA recovered from FLAG-Pof8 IP from extracts with untagged- and HA-tagged Pop100. Bars represent mean enrichment (± SEM, $n = 3$) relative to untagged-Pop100 and normalized to input. Statistical analysis: unpaired t tests ($n = 3$). (**B**) RT–qPCR analysis of RNA recovered from Trt1-V5 IP from extracts with untagged- and HA-tagged Pop100 as in (**A**). Statistical analysis: unpaired t tests ($n = 3$).

 © The Author(s)
