# Supplementary material for: RNase P/MRP subunits chaperone telomerase holoenzyme assembly in fission yeast
Source: EMBO Rep. 2026 Apr 28;27(12):3277–302. doi: 10.1038/s44319-026-00782-9 (PMC13303942; doi:10.1038/s44319-026-00782-9)
Supplement: Supplementary file 2 — Peer Review File [file 44319_2026_782_MOESM2_ESM.pdf]

EMBO reports

RNase P/MRP subunits chaperone telomerase holoenzyme assembly in fission yeast

Lili Pan, Valentine Patterson, Martin Moeckel, Rachel Helston, Raymund Wellinger, David Zappulla, and Peter Baumann

Corresponding author(s): Peter Baumann (peter@baumannlab.org)

Review Timeline:

Submission Date:	19th Sep 25
Editorial Decision:	29th Oct 25
Revision Received:	16th Feb 26
Editorial Decision:	24th Mar 26
Revision Received:	26th Mar 26
Accepted:	8th Apr 26

Editor: Esther Schnapp

Transaction Report:

(Note: With the exception of the correction of typographical or spelling errors that could be a source of ambiguity, letters and reports are not edited. Depending on transfer agreements, referee reports obtained elsewhere may or may not be included in this compilation. Referee reports are anonymous unless the Referee chooses to sign their reports.)

1st Editorial Decision **29th Oct 2025**

Dear Prof. Baumann

Thank you for the submission of your manuscript to EMBO reports. We have now received the full set of referee reports that is pasted below.

As you will see, all referees acknowledge that the findings are interesting. They also have some suggestions for how the study could be improved and I think all suggestions are good and should be addressed. Please let me know in case you disagree and we can discuss the exact revision requirements further, also in a video chat, if you like.

I would thus like to invite you to revise your manuscript with the understanding that the referee concerns must be fully addressed and their suggestions taken on board. Please address all referee concerns in a complete point-by-point response. Acceptance of the manuscript will depend on a positive outcome of a second round of review. It is EMBO reports policy to allow a single round of major revision only and acceptance or rejection of the manuscript will therefore depend on the completeness of your responses included in the next, final version of the manuscript.

We realize that it is difficult to revise to a specific deadline. In the interest of protecting the conceptual advance provided by the work, we recommend a revision within 3 months (29th Jan 2026). Please discuss the revision progress ahead of this time with the editor if you require more time to complete the revisions.

IMPORTANT NOTE: we perform an initial quality control of all revised manuscripts before re-review. Your manuscript will FAIL this control and the handling will be DELAYED if the following APPLIES:
1) A data availability section providing access to data deposited in public databases is missing. If you have not deposited any data, please add a sentence to the data availability section that explains that.
2) Your manuscript contains statistics and error bars based on n=2. Please use scatter blots in these cases. No statistics should be calculated if n=2.

When submitting your revised manuscript, please carefully review the instructions that follow below. Failure to include requested items will delay the evaluation of your revision.

1) a .docx formatted version of the manuscript text (including legends for main figures, EV figures and tables). Please make sure that the changes are highlighted to be clearly visible.

2) individual production quality figure files as .eps, .tif, .jpg (one file per figure). See https://wol-prod-cdn.literatumonline.com/pb-assets/embo-site/EMBOPress_Figure_Guidelines_061115-1561436025777.pdf for more info on how to prepare your figures.

3) We replaced Supplementary Information with Expanded View (EV) Figures and Tables that are collapsible/expandable online. A maximum of 5 EV Figures can be typeset. EV Figures should be cited as 'Figure EV1, Figure EV2" etc... in the text and their respective legends should be included in the main text after the legends of regular figures.

- For the figures that you do NOT wish to display as Expanded View figures, they should be bundled together with their legends in a single PDF file called *Appendix*, which should start with a short Table of Content. Appendix figures should be referred to in the main text as: "Appendix Figure S1, Appendix Figure S2" etc. See detailed instructions regarding expanded view here: <https://www.embopress.org/page/journal/14693178/authorguide#expandedview>

- Additional Tables/Datasets should be labeled and referred to as Table EV1, Dataset EV1, etc. Legends have to be provided in a separate tab in case of .xls files. Alternatively, the legend can be supplied as a separate text file (README) and zipped together with the Table/Dataset file.

4) a .docx formatted letter INCLUDING the reviewers' reports and your detailed point-by-point responses to their comments. As part of the EMBO Press transparent editorial process, the point-by-point response is part of the Review Process File (RPF), which will be published alongside your paper.

5) a complete author checklist, which you can download from our author guidelines <https://www.embopress.org/page/journal/14693178/authorguide>. Please insert information in the checklist that is also reflected in the manuscript. The completed author checklist will also be part of the RPF.

6) Please note that all corresponding authors are required to supply an ORCID ID for their name upon submission of a revised manuscript (<https://orcid.org/>). Please find instructions on how to link your ORCID ID to your account in our manuscript tracking system in our Author guidelines <https://www.embopress.org/page/journal/14693178/authorguide#authorshipguidelines>

7) Before submitting your revision, primary datasets produced in this study need to be deposited in an appropriate public

database (see https://www.embopress.org/page/journal/14693178/authorguide#datadeposition). Please remember to provide a reviewer password if the datasets are not yet public. The accession numbers and database should be listed in a formal "Data Availability" section placed after Materials & Method (see also https://www.embopress.org/page/journal/14693178/authorguide#datadeposition). Please note that the Data Availability Section is restricted to new primary data that are part of this study. * Note - All links should resolve to a page where the data can be accessed. *
If your study has not produced novel datasets, please mention this fact in the Data Availability Section.

8) At EMBO Press we ask authors to provide source data for the main manuscript figures. You will receive a separate email with instructions for providing source data with your revised manuscript, including how to upload and organize the files.

9) Our journal also encourages inclusion of *data citations in the reference list* to directly cite datasets that were re-used and obtained from public databases. Data citations in the article text are distinct from normal bibliographical citations and should directly link to the database records from which the data can be accessed. In the main text, data citations are formatted as follows: "Data ref: Smith et al, 2001" or "Data ref: NCBI Sequence Read Archive PRJNA342805, 2017". In the Reference list, data citations must be labeled with "[DATASET]". A data reference must provide the database name, accession number/identifiers and a resolvable link to the landing page from which the data can be accessed at the end of the reference. Further instructions are available at https://www.embopress.org/page/journal/14693178/authorguide#referencesformat

10) Regarding data quantification (see Figure Legends: https://www.embopress.org/page/journal/14693178/authorguide#figureformat)

The following points must be specified in each figure legend:

- the name of the statistical test used to generate error bars and P values,

- the number (n) of independent experiments (please specify technical or biological replicates) underlying each data point,

- the nature of the bars and error bars (s.d., s.e.m.),

- If the data are obtained from n Program fragment delivered error ``Can't locate object method "less" via package "than" (perhaps you forgot to load "than"?) at //ejpvfs23/sites23b/embor_www/letters/embor_decision_revise_and_review.txt line 56.' 2, use scatter blots showing the individual data points.

Discussion of statistical methodology can be reported in the materials and methods section, but figure legends should contain a basic description of n, P and the test applied.

- Please also include scale bars in all microscopy images.

11) The journal requires a statement specifying whether or not authors have competing interests (defined as all potential or actual interests that could be perceived to influence the presentation or interpretation of an article). In case of competing interests, this must be specified in your disclosure statement. Further information: https://www.embopress.org/competing-interests

12) All Materials and Methods need to be described in the main text using our 'Structured Methods' format, which is required for all research articles. According to this format, the Methods section includes a Reagents and Tools Table (listing key reagents, experimental models, software and relevant equipment and including their sources and relevant identifiers) followed by a Methods and Protocols section describing the methods using a step-by-step protocol format. The aim is to facilitate adoption of the methodologies across labs. More information on how to adhere to this format as well as a downloadable template (.docx) for the Reagents and Tools Table can be found in our author guidelines: https://www.embopress.org/page/journal/14693178/authorguide#structuredmethods.

An example of a Method paper with Structured Methods can be found here: https://www.embopress.org/doi/full/10.1038/s44320-024-00037-6#sec-4

We would also welcome the submission of cover suggestions, or motifs to be used by our Graphics Illustrator in designing a cover.

As part of the EMBO publication's Transparent Editorial Process, EMBO reports publishes online a Review Process File (RPF) to accompany accepted manuscripts. This File will be published in conjunction with your paper and will include the referee reports, your point-by-point response and all pertinent correspondence relating to the manuscript.

You are able to opt out of this by letting the editorial office know (emboreports@embo.org). If you do opt out, the Review

Process File link will point to the following statement: "No Review Process File is available with this article, as the authors have chosen not to make the review process public in this case."

I look forward to seeing a revised form of your manuscript when it is ready.

Best regards,
Esther

Esther Schnapp, PhD
Senior Editor
EMBO reports

Referee #1:

The reviewed manuscript reports on the identification of the Pop6, Pop7 and Pop100 proteins as components of the telomerase RNP complex in S. pombe. These proteins are subunits of two other essential RNP complexes, RNaseP and RNaseMRP, and thus the Pop proteins are also essential for cell viability. The authors identified a conserved internal loop (P3-like) in telomerase RNA (TER1), which serves as a binding site for the Pop proteins and analyzed the protein-RNA interactions and the consequences of their disruption on the telomerase complex assembly, RNA structure and telomerase activity. The authors conclude that the Pop proteins associate early in the biogenesis of telomerase and are essential for stabilizing the proper structure of the template-pseudoknot domain and the association of the telomerase proteins. The interaction of Pop proteins with telomerase proteins was reported in S. cerevisae. However, the position of their binding site within TLC1 (telomerase RNA) and their roles in the RNP complex assembly appear different between the species. The authors performed extensive in vivo structure probing by SHAPE-MaP of the WT and TER1 point mutant in the P3-like internal loop, which abolished Pop protein binding, in the context of WT or deletions for various telomerase proteins. The results are interesting and informative even beyond the context of the Pop proteins since they reveal alternative conformations, the dependency of protein subunits upon each other for the assembly, and the involvement of the three-way junction in the catalytic core assembly. The experiments are elegant and well designed and controlled, and the results are clear and carefully interpreted. Overall, the manuscript is clearly written and flows well. My main suggestion is to make the large part of SHAPE analysis more accessible to the reader and easier to understand and appreciate by explaining in more detail various structure alignments, and clarifying better the rational and interpretations. Also some of the legends are too laconic and can benefit from adding some more details. My minor comments are listed below.

1. Lines 200-202 and the legend to Fig. 2: The sequential IP scheme is not clear - which supernatants were taken for the subsequent IP, with which Ab were used in each? Please explain better.

2. Fig. 3A: I find it difficult to distinguish the green from the blue highlight. Can you separate them better?

3. Lines 262-264: Have the authors examined the predicted structure of the precursor? Is it possible that the longer sequences affect the P3 domain structure?

4. Fig. 3D: As I understand, the bar graph above MRP1 indicates the effect of the TER1 P3 mutation on the binding of the Pop proteins to MRP1. Can the authors clarify it better in the text or legend? Do the authors think that the effect on Pop100 binding to MRP1 is significant? Any idea how to explain it?

5. Lines 270-272 and Supp. Fig. 5B: It seems that del130-137 still retained some activity. This is consistent with the lower conservation that appears for this side of the loop (Fig. 3A), and the SHAPE results (lines 348-350), suggesting that the Pop proteins bind specifically to the other side of the loop (nt 923-930).

6. Lines 310-403: This part has a lot of interesting information but could benefit from a better explanation of the rational behind each structure comparison and of the results interpretations, and from better annotation within the figure of the regions referred to in the text. Consider adding a schematic model to illustrate on the TER1 structure the regions distinguished for the binding of Pop proteins, Trt1, Est1...

7. Lines 370-372: The rational behind this experiment is not clear. Why would the mutation in TER1 affect MRP1?

8. Lines: 398-402: What about the changes in nt 1053 and 1072 in Trt1del, and 1053 in the C926U mutant?

9. Line 475: To make it easy for the reader to understand the different context, I would start the sentence with 'In S. cerevisiae'...

10. Suppl. Fig. 1 legend: The numbers in parenthesis indicate the full length, right? Please indicate in the legend.

11. Suppl. Fig. 5 legend: How many streaks/cell divisions these strains were grown for?

Referee #2:

Telomerase is one of the most prominent examples for studying RNA-protein interaction and RNA biogenesis, as the catalytic function of this ribonucleoprotein complex relies on precise RNA folding and complex assembly. Unlike well-characterized human and tetrahymena telomerase RNA (TR), which are approximately 450 nt and 160 nt in length, respectively, the size of yeast TRs are substantially larger (>1200 nt), assembling into more complicated architectures and allowing associations of additional accessory proteins. In budding yeast S. cerevisiae, RNase P/MRP components Pop1, Pop6 and Pop7 have been identified as telomerase-associated factors via recognizing a P3-like RNA domain in TR and subsequently recruiting the catalytic subunits for telomerase. In this manuscript, authors identified Pop6, Pop7 and Pop100 (orthologs of Pop1, Pop6 and Pop7) as components of the active telomerase holoenzyme in fission yeast S. pombe, with several advances over prior works. In particular, they mapped Pop6/Pop7/Pop100 binding to the P3-like stem-loop element within TR, which is adjacent to the template-pseudoknot domain, distinct from the budding yeast. Notably, a single-nucleotide mutation (C926U) that diminishes Pop6/Pop7/Pop100 binding reduced telomerase activity and led to severe telomere shortening in yeast. In addition, SHAPE analyses indicated that Pop6/Pop7/Pop100 stabilize a productive TR conformation in both P3-like element and template-pseudoknot domain over a misfolded alternative, consistent with the function of Pop6/Pop7/Pop100 as RNA-folding chaperones that also enable recruitment of other RNA biogenesis factors. Overall, this work redefines RNase P/MRP subunits from peripheral interactors of S. cerevisiae telomerase to critical RNA-folding chaperones in S. pombe that promote telomerase assembly and are essential for telomerase function in yeast. Most conclusions of this manuscript are supported. I have few recommendations to improve clarity:
Major comments
1. Identification of Pop6 Ortholog in S. pombe. Although authors showed structural similarity of identified S. pombe Pop6 to S. cerevisiae Pop6 and evidence of Pop6-Pop7 interaction, a validation using Pop6 knockout or knockdown with expectation of RNase MRP loss-of-function phenotypes will be helpful. Notably, AlphaFold utilizes multi-sequence alignment as a principle for structure prediction. Therefore, concordance between the predicted S. pombe Pop6 structure and the S. cerevisiae Pop6 crystal structure should reflect underlying sequence conservation and not be considered as independent evidence.
2. Figure 1E. Figure legend states that the RNA enrichment from tagged-Pop IPs are compared to untagged controls, but the actual values for the untagged controls are not shown. Authors should plot those controls explicitly. Also, statistical analyses should indicate exactly which groups are being compared. Consider adding connecting lines between the relevant bars with the corresponding P-values above each comparison.
3. C926U compromises telomerase function. To examine the reduction of activity on telomerase with C926U-mutated TR, which abolishes Pop6-7-100 association, authors performed telomerase activity assays after Lsm4-IP and Pof8-IP. Because Lsm4 and Pof8 are TR biogenesis factors as Pop proteins, and later results suggest a synergistic effect, I recommend telomerase activity assays of the same WT and C926U samples from the enrichment of catalytic subunit (Trt1-IP) or non-specific protein-independent enrichment of telomerase (e.g. PEG-based precipitation) to unbiasedly measure telomerase activity.
4. Pop Binding Coincides with TR Structural Changes. The in vivo SHAPE results are convincing and satisfied.
Minor comments
1. Figure 1B bottom. What are the 100kDa bands detected by the anti-HA western blot in input samples. If they are non-specific detections of the antibody, authors should label it and note in the legend.
2, Figure 3D, Figure 5 and Figure 7C. Same as figure 1E. See my major comment 2.
3. Binding specificity of Pop6-7-100 and P3-like element. Selective recognition of Pop6-7-100 on P3-like stem-loop structure is one of the major conclusions in this story. Interestingly, figure 3C demonstrates the function of Pop6 providing RNA-binding specificity for Pop7 in the heterodimer. In vitro biochemical assays will be helpful for measuring binding affinity and specificity of Pop7 alone and Pop6-Pop7 heterodimer on P3-like element and C926U-mutated P3-like element. I suggest this experiment because authors have high quality recombinant proteins as shown in figure 1C.

Referee #3:

This manuscript reports the identification of fission yeast Pop100, Pop6 and Pop7 subunits of RNAse P/MRP as bona fide components of the telomerase RNP. The authors used co-immunoprecipitation and in vitro binding assay with recombinant proteins to show that Pop6, Pop7 and Pop100 interact with each other, and with the telomerase RNA subunit TER1. Telomerase activity can be detected in vitro following the pull-down of tagged Pop6, Pop7 or Pop100. A RNAse P/MRP P3-like stem-loop was identified in the TER1 RNA, close to the core T-PK region of the RNA. A minimal P3-like RNA probe can pull-down recombinant Pop6 and Pop7 in vitro, while the point mutation C926U in the P3 loop disrupts Pop6/7/100 binding. Expression of TER1 C926U mutant in vivo results in severe telomeres shortening, reduced telomerase activity and compromised telomerase RNP assembly. Finally, in vivo SHAPE-MaP analysis revealed major structural changes in the P3-like domain and T-PK core domain of TER1 in the absence of Pop proteins (C926U TER1 mutant and HA-Pop100 hypomorphic mutant). The authors

propose that Pop6/7/100 promote correct folding of the core T-PK region of TER1, enabling the binding of Pof8, Trt1 and Est1.

The finding that RNAse P/MRP subunits Pop100, Pop6 and Pop7 are part of the active telomerase RNP in fission yeast is clearly novel and supported by well-designed experiments. SHAPE-MaP and co-immunoprecipitation results also reveal an intriguing effect of reciprocal stabilization between the Pop proteins and other telomerase subunits interacting with TER1 RNA, like Pof8 and Trt1. The observation that Pop proteins are components of the active telomerase complex in both budding and fission yeasts, and that they play similar roles in telomerase biogenesis, offers a striking example of an evolutionary conserved role for these proteins in the assembly of catalytic RNPs. While I think this work could be of interest for the readers of this journal, several questions remain regarding some of the results:

Major comments:

- line 220 : "telomere length analysis revealed a modest shortening in strains expressing HA-tagged Pop7 or Pop100 (Supplementary Figure 3B)". Telomeres length shortening seems to be more than modest in the HA-tagged Pop7 and Pop100 strains. To have a more quantitative assessment, telomeres length should be measured in the tagged strains and compared to the untagged strain. Also, how different is telomeres length in the HA-tagged Pop7 and Pop100 compared to the C926 mutant strains in Figure 4A?

- There's also some contradiction between the claim that HA-tagged Pop100 has a "modest effect on telomeres length" and later in the text claiming that the HA-Pop100 is an hypomorphic mutant. This should be clarified.

- line 263: "...Pop6 and Pop7 still associated with the precursor form, suggesting that Pop6/Pop7 may associate in a broader range of structural contexts in the precursor." What is the meaning of "broader range of structural contexts"? Did the authors look at a P3-like stem-loop in the pre-TER1 RNA?

- Did the authors tried to knockout pop6? Is it lethal?

- Figure 5: to determine the impact of the Pop proteins on the interaction between TER1 and Pof8, Trt1 or Est1, co-IP experiments have been performed with the C926U TER1 mutant. However, this mutant has a strong effect on TER1 RNA T-PK core structure, and it is not clear if the loss of protein binding is due to the absence of the Pop proteins on the TER1 RNA or to RNA misfolding (or both). Since the HA-Pop100 strain has an hypomorphic phenotype, the authors could assess the interaction between TER1 and Pof8 and/or Trt1 by co-IP in a HA-Pop100 strain. This would strengthen the claim that Pop proteins are important for telomerase RNP assembly.

- Figure 6: the SHAPE-MaP data are quite interesting and suggest a different conformation for the pseudoknot region of TER1, where the pseudoknot stem 1 (PK1) is missing (Figure 6E), compared to the canonical T-PK core structure where PK1 is present (Figure 6D). However, the authors do not compare their results with the SHAPE analysis performed on the immunoprecipitated telomerase RNP published by Hu et al. (2020). In their paper, Hu et al show that the PK1 stem of TER1 has a low reactivity profile, which better supports the conformation of Figure 6D. Is it possible that a significant fraction of TER1 is not associated with Trt1, which could explain the differences between the SHAPE results? These differences should be discussed.

Minor comments:

- Figure 1B: what is the band at ~100 KDa in the Input fractions of the Western blot with anti-HA antibody?

Response to referees' comments

Referee #1:

The reviewed manuscript reports on the identification of the Pop6, Pop7 and Pop100 proteins as components of the telomerase RNP complex in S. pombe. These proteins are subunits of two other essential RNP complexes, RNaseP and RNaseMRP, and thus the Pop proteins are also essential for cell viability. The authors identified a conserved internal loop (P3-like) in telomerase RNA (TER1), which serves as a binding site for the Pop proteins and analyzed the protein-RNA interactions and the consequences of their disruption on the telomerase complex assembly, RNA structure and telomerase activity. The authors conclude that the Pop proteins associate early in the biogenesis of telomerase and are essential for stabilizing the proper structure of the template-pseudoknot domain and the association of the telomerase proteins. The interaction of Pop proteins with telomerase proteins was reported in S. cerevisae. However, the position of their binding site within TLC1 (telomerase RNA) and their roles in the RNP complex assembly appear different between the species. The authors performed extensive in vivo structure probing by SHAPE-MaP of the WT and TER1 point mutant in the P3-like internal loop, which abolished Pop protein binding, in the context of WT or deletions for various telomerase proteins. The results are interesting and informative even beyond the context of the Pop proteins since they reveal alternative conformations, the dependency of protein subunits upon each other for the assembly, and the involvement of the three-way junction in the catalytic core assembly. The experiments are elegant and well designed and controlled, and the results are clear and carefully interpreted. Overall, the manuscript is clearly written and flows well. My main suggestion is to make the large part of SHAPE analysis more accessible to the reader and easier to understand and appreciate by explaining in more detail various structure alignments, and clarifying better the rational and interpretations. Also some of the legends are too laconic and can benefit from adding some more details. My minor comments are listed below.

We thank reviewer 1 for their comments. We agree that the information from the SHAPE analysis was presented in a dense and not very accessible form. We have now extensively revised the text and added more details regarding rationale and interpretations. In addition, we have added more details to the legends to make the information more easily accessible.

1. Lines 200-202 and the legend to Fig. 2: The sequential IP scheme is not clear - which supernatants were taken for the subsequent IP, with which Ab were used in each? Please explain better.

We have added more details to the sequential IP scheme in the main text (lines 205-214, page 9) as well as the legend. We have also modified the scheme on top of the gel images in Figure 2C, 2E and 2G to make it clearer.

The text reads: "To further assess what fraction of active telomerase is associated with each Pop protein, we performed sequential immunoprecipitations followed by telomerase activity assays. Protein extracts from cells harboring both FLAG-tagged Pop6 and cMyc-tagged Lsm4 were first subjected to IP with either anti-FLAG antibody to immunoprecipitate Pop6 or with anti-V5 antibody as a negative control. The resulting supernatants from these IPs were then subjected to a second round of immunoprecipitation with anti–cMyc antibody to immunoprecipitate the fraction of telomerase that was not associated with Pop6 and hence remained in the supernatant of the first IP. Prior work had shown that Lsm4 was associated quantitatively with active telomerase (Tang *et al.*, 2012)."

2. Fig. 3A: I find it difficult to distinguish the green from the blue highlight. Can you separate them better?

We have changed the blue highlight to magenta to increase contrast.

3. Lines 262-264: Have the authors examined the predicted structure of the precursor? Is it possible that the longer sequences affect the P3 domain structure?

We have performed structure predictions for the precursor using various parameters, the 3' extension present in the precursor folds as an independent unit, which does not interact with or affect the folding of the P3 domain, the core region or the distal arm. This information is added as a new figure (Appendix Figure S5) and in the text (lines 292-297, page 13). "As structure predictions indicated that the sequence beyond the mature 3' end folds largely independently without impacting the folding of sequences present in the mature form (Appendix Fig. S5), it must be considered that Pop6/Pop7 make additional contacts with RNA sequences that are only present in the precursor or interact with proteins that are specifically associated with the precursor form."

4. Fig. 3D: As I understand, the bar graph above MRP1 indicates the effect of the TER1 P3 mutation on the binding of the Pop proteins to MRP1. Can the authors clarify it better in the text or legend? Do the authors think that the effect on Pop100 binding to MRP1 is significant? Any idea how to explain it?

The effect on Pop100 binding to MRP1 is not statistically significant. We previously only marked the significant changes with asterisks, and statistically insignificant changes were not marked. To better clarify this, we have now added the p values for all qPCR statistical analyses in the figures.

5. Lines 270-272 and Supp. Fig. 5B: It seems that del130-137 still retained some activity. This is consistent with the lower conservation that appears for this side of the loop (Fig. 3A), and the SHAPE results (lines 348-350), suggesting that the Pop proteins bind specifically to the other side of the loop (nt 923-930).

We thank the reviewer for pointing this out. We have modified the text in lines 302-305, page 13: "Telomere length analysis revealed that deletion of the 5' arm led to severe telomere shortening, and deletion of 3' arm or both arms led to complete telomere loss over 110 generations, confirming the essential role of this RNA element in telomere maintenance (Fig. EV3B)." And indeed, our data support that nt 923-930 are the main binding site for Pop proteins and thus are more important than the nt130-137 side of the loop. We are discussing this further in the context of the SHAPE analysis (lines 384-386, page 17).

6. Lines 310-403: This part has a lot of interesting information but could benefit from a better explanation of the rational behind each structure comparison and of the results interpretations, and from better annotation within the figure of the regions referred to in the text. Consider adding a schematic model to illustrate on the TER1 structure the regions distinguished for the binding of Pop proteins, Trt1, Est1...

We appreciate the reviewer pointing out that this section was interesting but not well explained. We have revised the text in this section (now lines 343-442) to clarify the rationale behind each structure comparison and more clearly state our interpretations.

We decided not to include an additional model beyond Figure 8 to distinguish the regions where specific proteins interact with TER1, as current data does not fully resolve the precise binding

sites and we do not want to mislead readers by portraying an architecture not fully supported by the available data.

7. Lines 370-372: The rational behind this experiment is not clear. Why would the mutation in TER1 affect MRP1?

The expectation was indeed that the mutation in TER1 would not affect MRP1. We merely examined the SHAPE profile of the corresponding region in MRP1 in this context to differentiate between stochastic fluctuations in signals and specific changes in profile. This was phrased poorly and generated confusion rather than increasing confidence in our data as intended. We have modified the text to make this clearer now (lines 405-410, pages 17-18):
"To confirm that these changes in reactivity are a specific consequence of the C926U mutation in TER1, we examined the SHAPE reactivity of the P3 loop in MRP1 from the two strains as a negative control. As expected, SHAPE reactivity of the P3 loop in MRP1 was unaffected by the C926U mutation in TER1, underscoring the specificity of the structural change in TER1 (Appendix Fig. S10A, B)."

8. Lines: 398-402: What about the changes in nt 1053 and 1072 in Trt1del, and 1053 in the C926U mutant?

While changes in reactivity of isolated nucleotides can reflect biologically relevant effects, it is often more difficult to distinguish such isolated signals from experimental noise. Upon reflection, we would therefore like to refrain from emphasizing each nucleotide position where a statistically significant change was observed. Instead, we have kept this section more general and readers can draw their own conclusions based on the deltaSHAPE maps shown in Appendix Figure S11. We hope that this work will be the starting point for further characterization of this RNP by us and others in the future.

The revised paragraph (lines 437-442, page 19) reads: "In contrast to the profound structural changes observed in the P3-like and T-PK regions, another essential element in telomerase RNA, the conserved three-way junction (TWJ; nt 1035–1096), remained largely unaffected in all mutant backgrounds (Appendix Fig. S11). Only a small number of nucleotides exhibited increased SHAPE reactivity in the *trt1Δ* and *ter1_C926U* mutant backgrounds consistent with the loss of Trt1 interaction (Appendix Fig. S11C, F)."

9. Line 475: To make it easy for the reader to understand the different context, I would start the sentence with 'In S. cerevisiae'...

Thank you for the suggestion. The text has been modified accordingly.

10. Suppl. Fig. 1 legend: The numbers in parenthesis indicate the full length, right? Please indicate in the legend.

Yes, this is correct. We have now added that information to the legend of Appendix Fig. S1 (formerly Suppl. Fig. 1).

11. Suppl. Fig. 5 legend: How many streaks/cell divisions these strains were grown for?

Cells were restreaked 5 times corresponding to approximately 110 generations. We have now added that information to the figure legend of Fig. EV3 (formerly Suppl. Fig. 5).

Referee #2:

Telomerase is one of the most prominent examples for studying RNA-protein interaction and RNA biogenesis, as the catalytic function of this ribonucleoprotein complex relies on precise RNA folding and complex assembly. Unlike well-characterized human and tetrahymena telomerase RNA (TR), which are approximately 450 nt and 160 nt in length, respectively, the size of yeast TRs are substantially larger (>1200 nt), assembling into more complicated architectures and allowing associations of additional accessory proteins. In budding yeast S. cerevisiae, RNase P/MRP components Pop1, Pop6 and Pop7 have been identified as telomerase-associated factors via recognizing a P3-like RNA domain in TR and subsequently recruiting the catalytic subunits for telomerase. In this manuscript, authors identified Pop6, Pop7 and Pop100 (orthologs of Pop1, Pop6 and Pop7) as components of the active telomerase holoenzyme in fission yeast S. pombe, with several advances over prior works. In particular, they mapped Pop6/Pop7/Pop100 binding to the P3-like stem-loop element within TR, which is adjacent to the template-pseudoknot domain, distinct from the budding yeast. Notably, a single-nucleotide mutation (C926U) that diminishes Pop6/Pop7/Pop100 binding reduced telomerase activity and led to severe telomere shortening in yeast. In addition, SHAPE analyses indicated that Pop6/Pop7/Pop100 stabilize a productive TR conformation in both P3-like element and template-pseudoknot domain over a misfolded alternative, consistent with the function of Pop6/Pop7/Pop100 as RNA-folding chaperones that also enable recruitment of other RNA biogenesis factors. Overall, this work redefines RNase P/MRP subunits from peripheral interactors of S. cerevisiae telomerase to critical RNA-folding chaperones in S. pombe that promote telomerase assembly and are essential for telomerase function in yeast. Most conclusions of this manuscript are supported. I have few recommendations to improve clarity:

We thank reviewer 2 for their constructive comments and for the summary that this work redefines RNase P/MRP subunits from peripheral interactors of S. cerevisiae telomerase to critical RNA-folding chaperones in S. pombe. Below we have addressed each of the comments.

Major comments

1. Identification of Pop6 Ortholog in S. pombe. Although authors showed structural similarity of identified S. pombe Pop6 to S. cerevisiae Pop6 and evidence of Pop6-Pop7 interaction, a validation using Pop6 knockout or knockdown with expectation of RNase MRP loss-of-function phenotypes will be helpful. Notably, AlphaFold utilizes multi-sequence alignment as a principle for structure prediction. Therefore, concordance between the predicted S. pombe Pop6 structure and the S. cerevisiae Pop6 crystal structure should reflect underlying sequence conservation and not be considered as independent evidence.

The sequence conservation between the orthologs is indeed low and our initial attempt to use BLAST to identify a ScPop6 ortholog in S. pombe was not successful. HHPred which uses structural prediction and homology search produced the initial hit. While the computational evidence is insufficient to conclude that we have identified a bona fide Pop6, we also provide several lines of experimental evidence in the manuscript. Firstly, the putative Pop6 protein is shown to interact with Pop7 and Pop100 (Figure 1A-C). Secondly, the putative Pop6 protein interacts with MRP1 and RRK1 RNA, indicating that it is part of the RNase P and MRP complexes (Fig. 1D, E). Thirdly, recombinant and purified Pop6 protein interacts with recombinant and purified Pop7 protein and binds to the P3-like domain in telomerase RNA further extending the biochemical similarities between the pombe protein and well-characterized Pop6 proteins from other species (Fig. 3C, EV2). We think that in aggregate these findings establish the S. pombe protein as a bona fide Pop6

ortholog. Nevertheless, we deleted one copy of Pop6 in a diploid S. pombe strain and attempted to recover haploid pop6Δ strains. Consistent with POP6 being an essential gene in S. cerevisiae, we were not able to recover viable gene deletions. This data is now shown as new Appendix Figure S2 and in the text lines 163-170, pages 7-8.

2. Figure 1E. Figure legend states that the RNA enrichment from tagged-Pop IPs are compared to untagged controls, but the actual values for the untagged controls are not shown. Authors should plot those controls explicitly. Also, statistical analyses should indicate exactly which groups are being compared. Consider adding connecting lines between the relevant bars with the corresponding P-values above each comparison.

We present the data as fold enrichment over the background amount of RNA that co-immunoprecipitates in control reactions where the same antibody against the epitope tag is incubated with extract of a control strain in which the protein is not tagged. This approach clearly shows the extent of specific versus non-specific binding. Due to the large differences in fold enrichment between the TER1 precursor and RRK1, the fold enrichment over the respective control samples is plotted on a log2 scale (as of -ΔΔCt of qPCR), but the y-axis is labelled in fold enrichment for easier accessibility to the general reader. We find this type of presentation clearer than the alternative of plotting of Ct values or representing the data as percentage of input. We understand that the reviewer would like to see the actual Ct values for the untagged control samples. These are listed in the source data file which will be published alongside the article and has been uploaded with the revised version of the manuscript.
The actual p values for each comparison have now been added to the figure panel, and which groups are being compared has been clarified in the legend.

3. C926U compromises telomerase function. To examine the reduction of activity on telomerase with C926U-mutated TR, which abolishes Pop6-7-100 association, authors performed telomerase activity assays after Lsm4-IP and Pof8-IP. Because Lsm4 and Pof8 are TR biogenesis factors as Pop proteins, and later results suggest a synergistic effect, I recommend telomerase activity assays of the same WT and C926U samples from the enrichment of catalytic subunit (Trt1-IP) or non-specific protein-independent enrichment of telomerase (e.g. PEG-based precipitation) to unbiasedly measure telomerase activity.

We have now performed telomerase activity assays from immunoprecipitates of extracts harboring tagged Trt1-V5. Consistent with prior results, these assays showed reduction of telomerase activity, confirming the telomerase activity is reduced with the C926U mutant. The new result is now included as part of Fig. 4C.

4. Pop Binding Coincides with TR Structural Changes. The in vivo SHAPE results are convincing and satisfied.

Thank you for referring to our SHAPE experiments as convincing and satisfying.

Minor comments
1. Figure 1B bottom. What are the 100kDa bands detected by the anti-HA western blot in input samples. If they are non-specific detections of the antibody, authors should label it and note in the legend.

This is indeed a non-specific detection by the anti-FLAG antibody from the first probing and is present in all input samples; the signal then persisted on the membrane after reprobing with anti-HA. The uncropped images are included as part of the source data to confirm this information.

The band has now been marked with an asterisk and the following sentence has been added to the legend: “The asterisk labels a non-specific band recognized by the anti-FLAG antibody from the first probing that is present in all input samples.“

2, Figure 3D, Figure 5 and Figure 7C. Same as figure 1E. See my major comment 2.

The p values for each comparison have now been added to the respective figure panels, and which groups are being compared has been clarified in the legends.

We decided to keep the original format of normalizing to the respective controls and plotting fold enrichment as this focuses the reader on the magnitude of enrichment relative to the respective control sample. This appears to be a matter of preference and if the reviewer and editor wishes us to present the data in a different format after reviewing the source data, we have no objections. All three ways of presenting such co-IP qRT-PCR data are commonly found in the literature.

3. Binding specificity of Pop6-7-100 and P3-like element. Selective recognition of Pop6-7-100 on P3-like stem-loop structure is one of the major conclusions in this story. Interestingly, figure 3C demonstrates the function of Pop6 providing RNA-binding specificity for Pop7 in the heterodimer. In vitro biochemical assays will be helpful for measuring binding affinity and specificity of Pop7 alone and Pop6-Pop7 heterodimer on P3-like element and C926U-mutated P3-like element. I suggest this experiment because authors have high quality recombinant proteins as shown in figure 1C.

To determine the dissociation constants of the binding between Pop6, Pop7, and the Pop6-Pop7 heterodimer with the P3-like domain we have now performed fluorescence polarization assays. This should have indeed been straight forward as the reviewer stated: “because authors have high quality recombinant proteins “. However, after delaying delivery of the labelled RNA oligos, Dharmacon canceled the order after apparently attempting synthesis 7 times with none of the products meeting quality control. We subsequently ordered slightly shorter RNA oligos (50nts, including the P3-like domain). These arrived promptly. Consistent with our in vitro binding experiments, Pop6 alone showed no binding to either RNA substrate. Pop7 bound to wt and C926U RNA with similar Kd (169 nM and 220 nM). In contrast, the Pop6-Pop7 heterodimer bound the WT RNA tightly (Kd 4 nM), whereas binding affinity was ~80 fold lower with the single nucleotide change at position 926 (Kd 309 nM). This data has been added to the manuscript as new Figure EV2 and main text lines 274-286, page 12.

Referee #3:

This manuscript reports the identification of fission yeast Pop100, Pop6 and Pop7 subunits of RNAse P/MRP as bona fide components of the telomerase RNP. The authors used co-immunoprecipitation and in vitro binding assay with recombinant proteins to show that Pop6, Pop7 and Pop100 interact with each other, and with the telomerase RNA subunit TER1. Telomerase activity can be detected in vitro following the pull-down of tagged Pop6, Pop7 or Pop100. A RNAse P/MRP P3-like stem-loop was identified in the TER1 RNA, close to the core T-PK region of the RNA. A minimal P3-like RNA probe can pull-down recombinant Pop6 and Pop7 in vitro, while the point mutation C926U in the P3 loop disrupts Pop6/7/100 binding. Expression of TER1 C926U mutant in vivo results in severe telomeres shortening, reduced telomerase activity and compromised telomerase RNP assembly. Finally, in vivo SHAPE-MaP analysis revealed major structural changes in the P3-like domain and T-PK core domain of TER1 in the absence of Pop proteins (C926U TER1 mutant and HA-Pop100 hypomorphic mutant). The authors propose that Pop6/7/100 promote correct

folding of the core T-PK region of TER1, enabling the binding of Pof8, Trt1 and Est1.

The finding that RNAse P/MRP subunits Pop100, Pop6 and Pop7 are part of the active telomerase RNP in fission yeast is clearly novel and supported by well-designed experiments. SHAPE-MaP and co-immunoprecipitation results also reveal an intriguing effect of reciprocal stabilization between the Pop proteins and other telomerase subunits interacting with TER1 RNA, like Pof8 and Trt1. The observation that Pop proteins are components of the active telomerase complex in both budding and fission yeasts, and that they play similar roles in telomerase biogenesis, offers a striking example of an evolutionary conserved role for these proteins in the assembly of catalytic RNPs. While I think this work could be of interest for the readers of this journal, several questions remain regarding some of the results:

We thank reviewer 3 for referring to our work as clearly novel and supported by well-designed experiments and a striking example of an evolutionary conserved role for these proteins. We appreciate the concerns and have addressed them point by point below:

Major comments:

- line 220 : "telomere length analysis revealed a modest shortening in strains expressing HA-tagged Pop7 or Pop100 (Supplementary Figure 3B)". Telomeres length shortening seems to be more than modest in the HA-tagged Pop7 and Pop100 strains. To have a more quantitative assessment, telomeres length should be measured in the tagged strains and compared to the untagged strain. Also, how different is telomeres length in the HA-tagged Pop7 and Pop100 compared to the C926 mutant strains in Figure 4A?

We have now quantified the length of the telomeric smear using the migration pixel distance relative to the marker to calculate the mean length for each sample. This allows for a more direct and quantitative comparison of length difference in mutants and WT samples.
The ∆length information has been added to Figure EV1B (formerly Suppl. Figure 3B) and Figure 4A and statements in the main text line 233 and 307 have been updated accordingly.

- There's also some contradiction between the claim that HA-tagged Pop100 has a "modest effect on telomeres length" and later in the text claiming that the HA-Pop100 is an hypomorphic mutant. This should be clarified.

We have modified the text to eliminate this apparent contradiction and represent the telomere length change in a quantitative rather than interpretative manner. Telomeres are ~85nt shorter in cells expressing HA-tagged Pop100, which corresponds to approximately 30% of wt telomere length and is indeed not "modest". However, it is consistent with the hypomorphic nature of tagged Pop100 characterized later in the manuscript. The text now reads:
"Additionally, telomere length analysis revealed a reduction in telomere lengths relative to wildtype by ~85nt in strains expressing HA-tagged Pop7 or Pop100 (Fig. EV1B)."

- line 263: "...Pop6 and Pop7 still associated with the precursor form, suggesting that Pop6/Pop7 may associate in a broader range of structural contexts in the precursor." What is the meaning of "broader range of structural contexts"? Did the authors look at a P3-like stem-loop in the pre-TER1 RNA?
We meant to speculate that Pop6 and Pop7 may associate with a different part of the RNA in the context of the precursor, or perhaps even indirectly through interaction with proteins bound to the precursor. Structure predictions for mature TER1 and the precursor form have now been added as a

new figure (Appendix Figure S5) and the main text lines 292-297 on page 13 has been updated to more clearly express this possibility: “As structure predictions indicated that the sequence beyond the mature 3’ end folds largely independently without impacting the folding of sequences present in the mature form (Appendix Fig. S5), it must be considered that Pop6/Pop7 make additional contacts with RNA sequences that are only present in the precursor or interact with proteins that are specifically associated with the precursor form.”

- Did the authors tried to knockout pop6? Is it lethal?

We indeed attempted unsuccessfully to generate pop6 deletions. We were able to delete one copy of pop6 in a diploid strain. Sporulation of this strain and random spore analysis revealed no viable progeny harboring the deletion. A new Figure (Appendix Figure S2) has now been added to show this and the main text has been updated lines 163-170, pages 7-8 “. Attempts to knockout *pop6*$^{+}$ in a haploid WT cell did not yield colonies, while making a heterozygous diploid *pop6*$^{+}$/*pop6*Δ strain was successful. We then performed random spore analysis for two independent isolates. For 230 and 93 spores that formed colonies on non-selective media, zero colonies were observed on plates containing G418 to select for cells harboring the *pop6* deletion (Appendix Fig. S2). These results are consistent with *pop6*$^{+}$ being an essential gene in *S. pombe*, similar to its ortholog in *S. cerevisiae* (Chamberlain *et al*, 1998) and consistent with the essential functions of RNase P/MRP.”

- Figure 5: to determine the impact of the Pop proteins on the interaction between TER1 and Pof8, Trt1 or Est1, co-IP experiments have been performed with the C926U TER1 mutant. However, this mutant has a strong effect on TER1 RNA T-PK core structure, and it is not clear if the loss of protein binding is due to the absence of the Pop proteins on the TER1 RNA or to RNA misfolding (or both). Since the HA-Pop100 strain has an hypomorphic phenotype, the authors could assess the interaction between TER1 and Pof8 and/or Trt1 by co-IP in a HA-Pop100 strain. This would strengthen the claim that Pop proteins are important for telomerase RNP assembly.

We thank the reviewer for this suggestion, and we have now assessed the interaction between TER1 and Popf8 and Trt1 in the context of untagged or HA-tagged Pop100 background. The Pof8 - TER1 interaction is 0.41 in HA-Pop100 background compared to untagged Pop100 and 0.77 for Trt1. This is consistent with the SHAPE data and the proposed role for the Pop proteins in telomerase RNP assembly. This data has been added as a new figure (Figure EV5) and is described in the main text on lines 414-418 of page 18: “This was also supported by the reactivity pattern in the hypomorphic HA-Pop100 strain, where a similar but less pronounced pattern was observed (Fig. 6F, G, bottom panels; 6H, right). Furthermore, HA-Pop100 also reduced Pof8 and Trt1 association with TER1 in immunoprecipitations, (Fig. EV5A, B), but to a lesser extent compared to TER1 C926U mutant (Fig. 5C, D).”

- Figure 6: the SHAPE-MaP data are quite interesting and suggest a different conformation for the pseudoknot region of TER1, where the pseudoknot stem 1 (PK1) is missing (Figure 6E), compared to the canonical T-PK core structure where PK1 is present (Figure 6D). However, the authors do not compare their results with the SHAPE analysis performed on the immunoprecipitated telomerase RNP published by Hu et al. (2020). In their paper, Hu et al show that the PK1 stem of TER1 has a low reactivity profile, which better supports the conformation of Figure 6D. Is it possible that a significant fraction of TER1 is not associated with Trt1, which could explain the differences between the SHAPE results? These differences should be discussed.

This is an interesting comparison and we have added it to the discussion on the text lines 509-518 of page 22: “Our SHAPE reactivity data agrees largely with a previous study where SHAPE reactivity of

the PK region was accessed for Trt1-cMyc associated TER1 (Hu *et al.*, 2020). Interestingly, in this study, the 5' arm of the PK1 stem had low reactivity while in ours it had medium to high reactivity. This could be explained by Trt1 associating preferentially with the fraction of TER1 that adopts the pseudoknot structure shown in Fig. 6D. However, the possibility that the difference in reactivity is related to the use of different SHAPE chemicals and read-outs cannot be excluded. Further deconvolution analysis of the PK region may reveal alternative structures in vivo and identify structure-specific binding preferences for different protein subunits."

Minor comments:

- Figure 1B: what is the band at ~100 KDa in the Input fractions of the Western blot with anti-HA antibody?

The blot was first probed with anti-FLAG antibody and then reprobed with anti-HA antibody. The band at ~100 kDa is a non-specific signal from the anti-FLAG antibody of the first probing. It is present at the same intensity in both untagged and HA-tagged Pop100 samples.
This band is now labeled in Figure 1B and the figure legend. The full images of the blots are also available in the source data for verification.

Dear Peter,

Thank you for the submission of your revised manuscript. We have now received the enclosed reports from the referees that were asked to assess it and I am happy to say that we can in principle accept your ms now. Only a few editorial requests will need to be addressed before we can proceed with the official acceptance of your manuscript:

- The author credits need to be removed from the ms file. All credits need to be entered during online ms submission.

- A callout is missing for Fig. 6H, please add.

- In the Appendix file, the figure legends should always follow their figures, please correct.

- Please supply source data for Appendix figure S2B. The blots appear pixellated under image analysis.

- The source data for Fig 2G seem to be missing, please add.

EMBO press papers are accompanied online by A) a short (1-2 sentences) summary of the findings and their significance, B) 2-3 bullet points highlighting key results and C) a synopsis image that is exactly 550 pixels wide and 200-600 pixels high (the height is variable). The synopsis image should provide a sketch of the major findings, like a graphical abstract. Please note that text needs to be readable at the final size. Please send us this information along with the final manuscript.

I look forward to seeing a final version of your manuscript as soon as possible.

It should be possible to move forward all current ms files to the next V3 version and you can then replace only the files that need to be replaced.

Best regards,
Esther

Esther Schnapp, PhD
Senior Editor
EMBO reports

Referee #2:

I have reviewed the revised manuscript and the authors' point-by-point response. The revision has improved the paper, and the authors have addressed my previous major concerns with new data . In particular, they now provide additional genetic support that pop6 is essential in S. pombe, biochemical experiments examining Pop6/Pop7 binding to the P3-like RNA element, and evidence to support Pop function contributing to recruitment of Pof8 and Trt1. Together, these additions make the central conclusion much stronger.
I also think the authors have addressed Reviewer 1's main concern. The SHAPE section is clearer in the revised manuscript, with improved explanation of the rationale for the structural comparisons, a more explicit negative-control interpretation using MRP1, and a discussion reconciling their PK1 observations with prior work.

Referee #3:

The authors have properly answered the comments of the reviewers. This manuscript is now acceptable for publication in this journal.

The authors have addressed all minor editorial requests.

Prof. Peter Baumann
Johannes Gutenberg University (JGU)
Faculty of Biology
Hanns-Dieter-Hüsch-Weg 15
Mainz 55128
Germany

Dear Peter,

I am very pleased to accept your manuscript for publication in the next available issue of EMBO reports. Thank you for your contribution to our journal.

Your manuscript will be processed for publication by EMBO Press. It will be copy edited and you will receive page proofs prior to publication. Please note that you will be contacted by Springer Nature Author Services to complete licensing and payment information.

You may qualify for financial assistance for your publication charges - either via a Springer Nature fully open access agreement or an EMBO initiative. Check your eligibility: https://link.springer.com/journal/44319/how-to-publish-with-us

Should you be planning a Press Release on your article, please get in contact with embo_production@springernature.com as early as possible in order to coordinate publication and release dates.

If you have any questions, please do not hesitate to contact the Editorial Office. Thank you for your contribution to EMBO Reports.

Best regards,
Esther

Esther Schnapp, PhD
Senior Editor
EMBO reports

>>> Please note that it is EMBO Reports policy for the transcript of the editorial process (containing referee reports and your response letter) to be published as an online supplement to each paper. If you do NOT want this, you will need to inform the Editorial Office via email immediately. More information is available here: https://link.springer.com/partners/embo-press/editorial-policies#Peer%20review

EMBO Press Author Checklist

Corresponding Author Name: Peter Baumann	
Journal Submitted to: EMBO Reports	
Manuscript Number: EMBOR-2025-62784-T	

USEFUL LINKS FOR COMPLETING THIS FORM
The EMBO Journal - Author Guidelines
EMBO Reports - Author Guidelines
Molecular Systems Biology - Author Guidelines
EMBO Molecular Medicine - Author Guidelines

Reporting Checklist for Life Science Articles (updated January
This checklist is adapted from Materials Design Analysis Reporting (MDAR) Checklist for Authors. MDAR establishes a minimum set of requirements in transparent reporting in the life sciences (see Statement of Task: 10.31222/osf.io/9sm4x). Please follow the journal's guidelines in preparing your
Please note that a copy of this checklist will be published alongside your article.

Abridged guidelines for figures
1. Data
The data shown in figures should satisfy the following conditions:
- the data were obtained and processed according to the field's best practice and are presented to reflect the results of the experiments in an accurate and unbiased manner.
- ideally, figure panels should include only measurements that are directly comparable to each other and obtained with the same assay.
- plots include clearly labeled error bars for independent experiments and sample sizes. Unless justified, error bars should not be shown for technical
- if n<5, the individual data points from each experiment should be plotted. Any statistical test employed should be justified.
- Source Data should be included to report the data underlying figures according to the guidelines set out in the authorship guidelines on Data

2. Captions
Each figure caption should contain the following information, for each panel where they are relevant:
- a specification of the experimental system investigated (eg cell line, species name).
- the assay(s) and method(s) used to carry out the reported observations and measurements.
- an explicit mention of the biological and chemical entity(ies) that are being measured.
- an explicit mention of the biological and chemical entity(ies) that are altered/varied/perturbed in a controlled manner.
- the exact sample size (n) for each experimental group/condition, given as a number, not a range;
- a description of the sample collection allowing the reader to understand whether the samples represent technical or biological replicates (including how many animals, litters, cultures, etc.).
- a statement of how many times the experiment shown was independently replicated in the laboratory.
- definitions of statistical methods and measures:
  - common tests, such as t-test (please specify whether paired vs. unpaired), simple χ2 tests, Wilcoxon and Mann-Whitney tests, can be unambiguously identified by name only, but more complex techniques should be described in the methods section;
  - are tests one-sided or two-sided?
  - are there adjustments for multiple comparisons?
  - exact statistical test results, e.g., P values = x but not P values < x;
  - definition of 'center values' as median or average;
  - definition of error bars as s.d. or s.e.m.

Please complete ALL of the questions below.
Select "Not Applicable" only when the requested information is not relevant for your study.

Materials

Newly Created Materials	Information included in the manuscript?	In which section is the information available? (Reagents and Tools Table, Materials and Methods, Figures, Data Availability Section)
New materials and reagents need to be available; do any restrictions apply?	Yes	Reagents and Tools Table, Methods, Appendix Table S1, S2, S3

Antibodies	Information included in the manuscript?	In which section is the information available? (Reagents and Tools Table, Materials and Methods, Figures, Data Availability Section)
For **antibodies** provide the following information:<br>- Commercial antibodies: RRID (if possible) or supplier name, catalogue number and or/clone number<br>- Non-commercial: RRID or citation	Yes	Reagents and Tools Table

DNA and RNA sequences	Information included in the manuscript?	In which section is the information available? (Reagents and Tools Table, Materials and Methods, Figures, Data Availability Section)
**Short novel DNA or RNA including primers, probes**: provide the sequences.	Yes	Appendix Table S3

Cell materials	Information included in the manuscript?	In which section is the information available? (Reagents and Tools Table, Materials and Methods, Figures, Data Availability Section)
**Cell lines:** Provide species information, strain. Provide accession number in repository **OR** supplier name, catalog number, clone number, and/**OR** RRID.	Not Applicable	
**Primary cultures:** Provide species, strain, sex of origin, genetic modification status.	Not Applicable	
Report if the cell lines were recently **authenticated** (e.g., by STR profiling) and tested for mycoplasma contamination.	Not Applicable	

Experimental animals	Information included in the manuscript?	In which section is the information available? (Reagents and Tools Table, Materials and Methods, Figures, Data Availability Section)
**Laboratory animals or Model organisms:** Provide species, strain, sex, age, genetic modification status. Provide accession number in repository **OR** supplier name, catalog number, clone number, **OR** RRID.	Not Applicable	
**Animal observed in or captured from the field:** Provide species, sex, and age where possible.	Not Applicable	
Please detail **housing and husbandry conditions**.	Not Applicable	

Plants and microbes	Information included in the manuscript?	In which section is the information available? (Reagents and Tools Table, Materials and Methods, Figures, Data Availability Section)
**Plants:** provide species and strain, ecotype and cultivar where relevant, unique accession number if available, and source (including location for collected wild specimens).	Not Applicable	
**Microbes:** provide species and strain, unique accession number if available, and source.	Yes	Methods, Appendix Table S1

Human research participants	Information included in the manuscript?	In which section is the information available? (Reagents and Tools Table, Materials and Methods, Figures, Data Availability Section)
If collected and within the bounds of privacy constraints report on age, sex and gender or ethnicity for all study participants.	Not Applicable	

Core facilities	Information included in the manuscript?	In which section is the information available? (Reagents and Tools Table, Materials and Methods, Figures, Data Availability Section)
If your work benefited from core facilities, was their service mentioned in the acknowledgments section?	Yes	Acknowledgements

Design

Study protocol	Information included in the manuscript?	In which section is the information available? (Reagents and Tools Table, Materials and Methods, Figures, Data Availability Section)
If study protocol has been **pre-registered, provide DOI in the manuscript**. For clinical trials, provide the trial registration number **OR** cite DOI.	Not Applicable	
Report the **clinical trial registration number** (at ClinicalTrials.gov or equivalent), where applicable.	Not Applicable	

Laboratory protocol	Information included in the manuscript?	In which section is the information available? (Reagents and Tools Table, Materials and Methods, Figures, Data Availability Section)
Provide DOI OR other citation details if **external detailed step-by-step protocols** are available.	Not Applicable	

Experimental study design and statistics	Information included in the manuscript?	In which section is the information available? (Reagents and Tools Table, Materials and Methods, Figures, Data Availability Section)
Include a statement about **sample size** estimate even if no statistical methods were used.	Yes	Methods, Figure legends
Were any steps taken to minimize the effects of subjective bias when allocating animals/samples to treatment (e.g. **randomization procedure**)? If yes, have they been described?	Not Applicable	
Include a statement about **blinding** even if no blinding was done.	Not Applicable	
Describe **inclusion/exclusion criteria** if samples or animals were excluded from the analysis. Were the criteria pre-established?<br><br>If sample or data points were omitted from analysis, report if this was due to attrition or intentional exclusion and provide justification.	Not Applicable	
For every figure, are **statistical tests** justified as appropriate? Do the data meet the assumptions of the tests (e.g., normal distribution)? Describe any methods used to assess it. Is there an estimate of variation within each group of data? Is the variance similar between the groups that are being statistically compared?	Yes	Methods, Figure legends

Sample definition and in-laboratory replication	Information included in the manuscript?	In which section is the information available? (Reagents and Tools Table, Materials and Methods, Figures, Data Availability Section)
In the figure legends: state number of times the experiment was **replicated** in laboratory.	Yes	Figure legends
In the figure legends: define whether data describe **technical or biological replicates**.	Yes	Figure legends

Ethics

Ethics	Information included in the manuscript?	In which section is the information available? (Reagents and Tools Table, Materials and Methods, Figures, Data Availability Section)
Studies involving **human participants**: State details of **authority granting ethics approval** (IRB or equivalent committee(s), provide reference number for approval.	Not Applicable	
Studies involving **human participants**: Include a statement confirming that **informed consent** was obtained from all subjects and that the experiments conformed to the principles set out in the WMA Declaration of Helsinki and the Department of Health and Human Services Belmont Report.	Not Applicable	
Studies involving **human participants:** For publication of **patient photos**, include a statement confirming that consent to publish was obtained.	Not Applicable	
Studies involving experimental **animals**: State details of **authority granting ethics approval** (IRB or equivalent committee(s), provide reference number for approval. Include a statement of compliance with ethical regulations.	Not Applicable	
Studies involving **specimen and field samples:** State if relevant **permits** obtained, provide details of authority approving study; if none were required, explain why.	Not Applicable	

Dual Use Research of Concern (DURC)	Information included in the manuscript?	In which section is the information available? (Reagents and Tools Table, Materials and Methods, Figures, Data Availability Section)
Could your study fall under dual use research restrictions? Please check biosecurity documents and list of **select agents and toxins** (CDC): https://www.selectagents.gov/sat/list.htm	Not Applicable	
If you used a select agent, is the security level of the lab appropriate and reported in the manuscript?	Not Applicable	
If a study is subject to dual use research of concern regulations, is the name of the **authority granting approval and reference number** for the regulatory approval provided in the manuscript?	Not Applicable	

Reporting

The MDAR framework recommends adoption of discipline-specific guidelines, established and endorsed through community initiatives. Journals have their own policy about requiring specific guidelines and recommendations to complement MDAR.

Adherence to community standards	Information included in the manuscript?	In which section is the information available? (Reagents and Tools Table, Materials and Methods, Figures, Data Availability Section)
State if relevant guidelines or checklists (e.g., **ICMJE, MIBBI, ARRIVE, PRISMA**) have been followed or provided.	Not Applicable	
For **tumor marker prognostic studies,** we recommend that you follow the **REMARK** reporting guidelines (see link list at top right). See author guidelines, under 'Reporting Guidelines'. Please confirm you have followed these guidelines.	Not Applicable	
For **phase II and III randomized controlled trials**, please refer to the **CONSORT** flow diagram (see link list at top right) and submit the CONSORT checklist (see link list at top right) with your submission. See author guidelines, under 'Reporting Guidelines'. Please confirm you have submitted this list.	Not Applicable	

Data Availability

Data availability	Information included in the manuscript?	In which section is the information available? (Reagents and Tools Table, Materials and Methods, Figures, Data Availability Section)
Have **primary datasets** been deposited according to the journal's guidelines (see 'Data Deposition' section) and the respective accession numbers provided in the Data Availability Section?	Yes	Data Availability
Were **human clinical and genomic datasets** deposited in a public access-controlled repository in accordance to ethical obligations to the patients and to the applicable consent agreement?	Not Applicable	
Are **computational models** that are central and integral to a study available without restrictions in a machine-readable form? Were the relevant accession numbers or links provided?	Yes	Data Availability
If publicly available data were reused, provide the respective **data citations in the reference list**.	Not Applicable	
